# The colours of Rome in the walls of Cástulo (Linares, Spain)

**DOI:** 10.1038/s41598-020-69334-y

**Published:** 2020-07-29

**Authors:** José Tuñón, Alberto Sánchez, David J. Parras, Pilar Amate, Manuel Montejo, Bautista Ceprián

**Affiliations:** 1 University Research Institute for Iberian Archaeology, University of Jaén, Campus Las Lagunillas s/n, Edif. C6, 23071 Jaén, Spain; 2 Department of Physical and Analytical Chemistry, University of Jaén, Campus Las Lagunillas s/n, Edif. B3, 23071 Jaén, Spain

**Keywords:** Characterization and analytical techniques, Analytical chemistry, Structural materials, Raman spectroscopy

## Abstract

Wall paintings have become one of the most relevant, complex and challenging research subjects in Archaeometry. Minimally- or non-invasive, accurate and multidisciplinary methods are needed to successfully address the problems posed by their physical and chemical properties and by their analysis techniques. Specifically, the analytical method implemented for the study of this type of samples must enable a precise separation of the chemical information from backgrounds and scenes, allowing the identification of pigment’s components in overlapping layers, the detection of minority components and the elucidation of pigment mixtures. Thus, this paper puts forward a multidisciplinary approach towards these goals by means of the combined use of micro Energy Dispersive X-ray Fluorescence (µEDXRF) surface mapping and single-spot micro-Raman spectroscopy and µEDXRF analysis. The samples under research come from the site of Cástulo (Linares, Spain), one of the most important Roman cities in the Iberian Peninsula. It must be emphasized the uniqueness of the walls of Cástulo, their optimal conservation state and the richness and variety of the colour’s palette used in their decoration, which make them an excellent and representative example of Roman wall paintings.

## Introduction

The Roman immovable and movable heritage of Europe is outstanding not only in terms of quantity but also of quality. As a research subject, it pursues both historical reconstruction and the design of preservation and restoration strategies and diagnostic procedures. It is, thus, a major area of development for archaeometric analysis, especially as regards spectroscopy. Wall paintings are particularly remarkable in this regard. A major component in the decoration of public and private buildings, it is one of the most attractive heritage goods of museums and sites. Research on wall paintings and on their preservation is a source of archaeometric projects intended to retrieve as much information as possible with the least possible alteration or damage to such rich and invaluable decoration.

Wall paintings have typically been researched based on simple decorative patterns or on single-spot analysis of samples, sometimes amounting to just a few. In most cases, the aim was the identification of the pigments and the binders used^1–4^, a better knowledge of how the paintings were made^5–7^, and proper understanding of the processes that may damage the painted surfaces^8–11^. The results obtained are often under the influence of the specific properties of the paint layers, of the raw materials used and of the limitations of the analytical techniques used.

This paper therefore sets out to put forward and test a chemical analysis procedure for general use in research on wall paintings. The paper relies on research on a wide gamut of colours, on a significant number of samples, and on the experience gained from addressing a range of difficulties found for the use of certain techniques and posed by the properties of the pigments under research.

Methodological improvements are mainly with regard to spectroscopy analysis. While MRS and µEDXRF techniques have been used widely in Roman contexts, issues remain in the application and subsequent interpretation of the results obtained therefrom, e.g.: the single-spot analysis technique, the limited number of spots considered (which may not be representative of the whole area under research), sample heterogeneity, overlapping colours, the thickness of the colour layer, or the sample’s fluorescence or interferences caused by the solid matrix near the material studied^12^. These problems can be addressed by the systematic scan of the surfaces using µEDXRF and the joint analysis of the maps obtained in terms of the semi-quantitative elemental composition and the single-spot MRS measurements results.

Albeit in limited cases, elemental mapping technique for research on wall paintings is attested in the specialized literature. Examples are the paintings of the Roman *villae* of Mülheim-Kärlich and Wössingen (Germany) (using a synchrotron source of X-rays)^13^^,^ of the Roman town *Ulpia Traiana Sarmizegetusa* (Romania) (by scanning electron microscopy with energy dispersive X-ray spectroscopy)^14^^,^ and of the *Villa dei Quintili* (Rome), for which handheld equipment was used^15^. Moreover, XRF mapping in its different modalities has also been implemented in different types of archaeological materials such as the ink of a papyrus of Herculaneum^16^ and on the Fayum painting ‘Portrait of a woman’, of the 2nd ct. a.D., from the collection of the National Gallery of Art in Washington DC^17^. More recent paintings that have also been analysed using similar mapping procedures are the Renaissance frescoes ‘The Loggia of Cupid and Psyche’ in Villa Farnesina (Rome)^18^ or the ‘Portrait of a Woman’ by Edgar Degas^19^.

Validation of the method presented here relies on research on samples retrieved from Cástulo (Linares, Spain). This Roman town had enough political, economic and geostrategic relevance as to host major public and private buildings with decoration according to the patterns and style of such major towns as Pompeii and Rome.

The archaeological site of Cástulo lies on the right bank of River Guadalimar, in Linares (Spain) (Supplementary Fig. S1). It is one of the main settlements of the Ibero-Roman period, both for the surface covered by its walls (50 ha) and for its strategic location high in the upper Guadalquivir Valley, near the silver mines of Sierra Morena. Late in the 3rd ct. b.C., Cástulo became involved in the 2nd Punic War, as Romans and Carthaginians strived to control one of the richest areas as regards human and material resources. In 206 b.C., General Publius Cornellius Scipio eventually conquered Cástulo for Rome, and it remained within the Roman Empire until the fall following the crisis of the 3rd ct. a.D.

**Figure 1:**
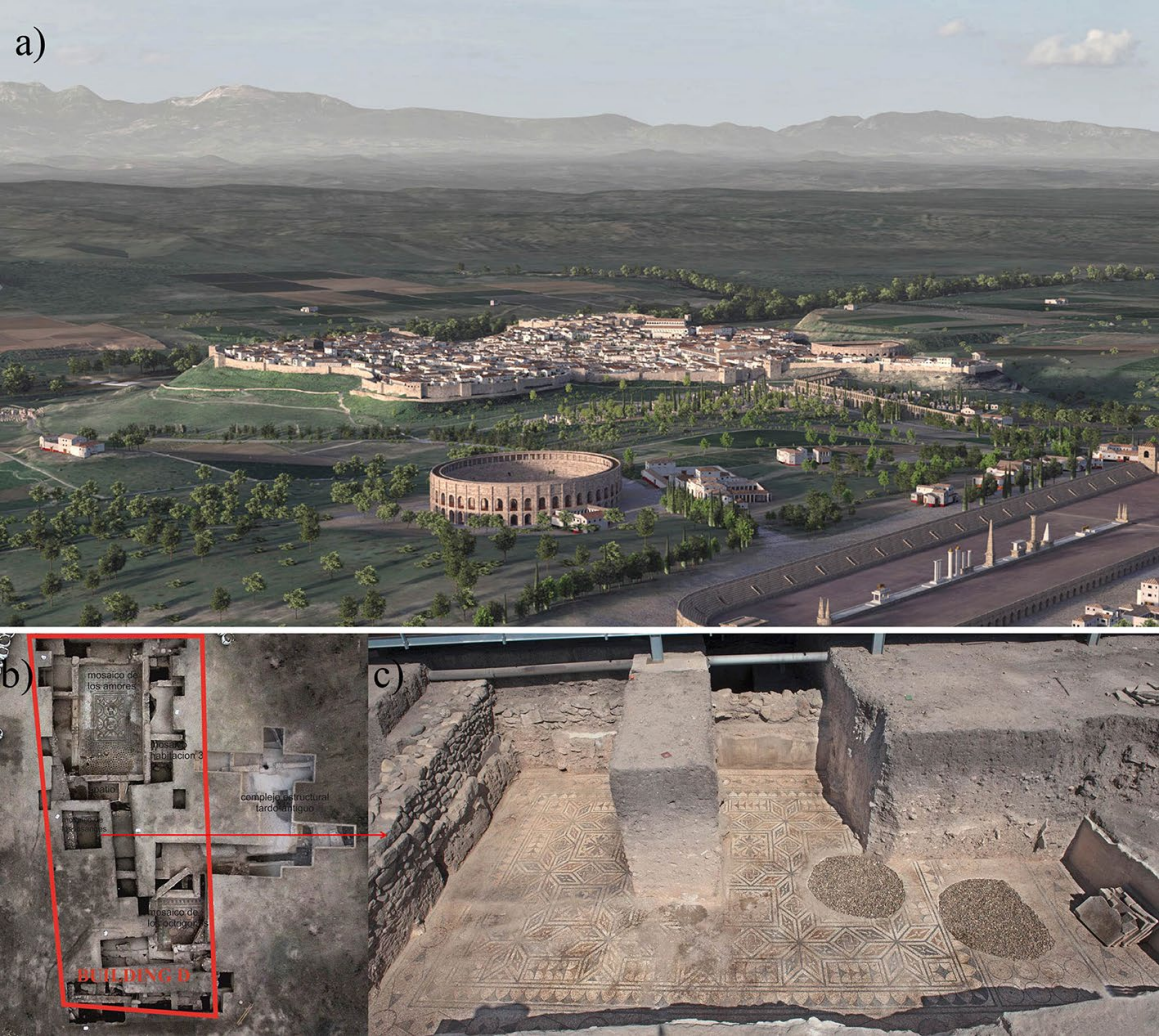
(**a**) Virtual reconstruction of the Roman city of Cástulo^27^. (**b**) Building D, area 2 of Cástulo. (**c**) Room 6 in the Building D (Photos: Francisco Arias de Haro).

As a Roman settlement, it reached a significant peak late in the 1st ct. a.D., with a theatre decorated with sculptures, therms, lavatories, a forum and an amphitheatre for gladiator games. Cástulo also built a supply network with aqueducts, channelling, tanks and fountains^20,21^ (Fig. 1a). Its richness during the High Empire contrasts with the decline during the Empire’s crisis in the 3rd ct. a.D. Still, Cástulo recovered in the 4th and 5th ct. a.D.^22^ and remained inhabited during the Muslim period and during the Castilian conquest early in the 12th ct. The site was finally abandoned in the mid-15th ct. after several attempts to repopulate it^20,21^.

The 2011 excavation of Cástulo unearthed a large building of ca. 33×12m decorated with impressive mosaics and wall paintings (Building D)^23–26^. The building dates back to the end of the 1st ct. a.D. It may have been built to honour Emperor Domitian (51–96 a.D.) and must therefore have been a public building (Fig. 1b). It must also have been part of an Imperial project, and probably exhibited *maiestas imperii* to exalt the Emperor as the Empire’s head in accordance with a policy that went as far back as August’s distinction of the Julio-Claudian dynasty^24^.

The building does not show physical wear or degradation that may have resulted in fractures, fissures or collapse after abandonment. This suggests that it may have never been used, as it was never completed^23^. A decision to demolish it was taken in accordance with Domitian’s *damnatio memoriae*, i.e. the condemnation of the memory of the last emperor of the Flavian dynasty: the conspiracy that led to his assassination in 96 a.D. was followed by the Senate’s decree to condemn his memory and destroy his works, so the building was left in ruins as a symbol of the Senate’s power.

The samples discussed here are part of the decoration of the walls of Room 6 of Building D (Fig. 1c). Unlike other studies of Cástulo, limited in scope^27^ and methodological rigour^28^^,^ this is intended as a fully comprehensive approach. Aiming at a level beyond that of case studies, it puts into practice the method described above and discusses a variety of problems and responses that can be used for research on wall paintings in general.

The samples are representative of most of the colours and chromatic gamut that were typically used for wall painting in the villas and palaces of the Roman Empire. The paper researches white, yellow, red/rose, brown, blue and green, i.e. virtually all the tones used in Roman painting and described by the three main classical authors (Theophrastus, Vitruvius and Pliny the Elder) as regards their basic colours, namely: yellow, red, white, black, blue, green and purple. Shades of these were obtained varying their composition or mixing them with other colours^3,29–31^. Of the latter list, purple is not present in the sample and is therefore not discussed in this paper.

**Figure 2:**
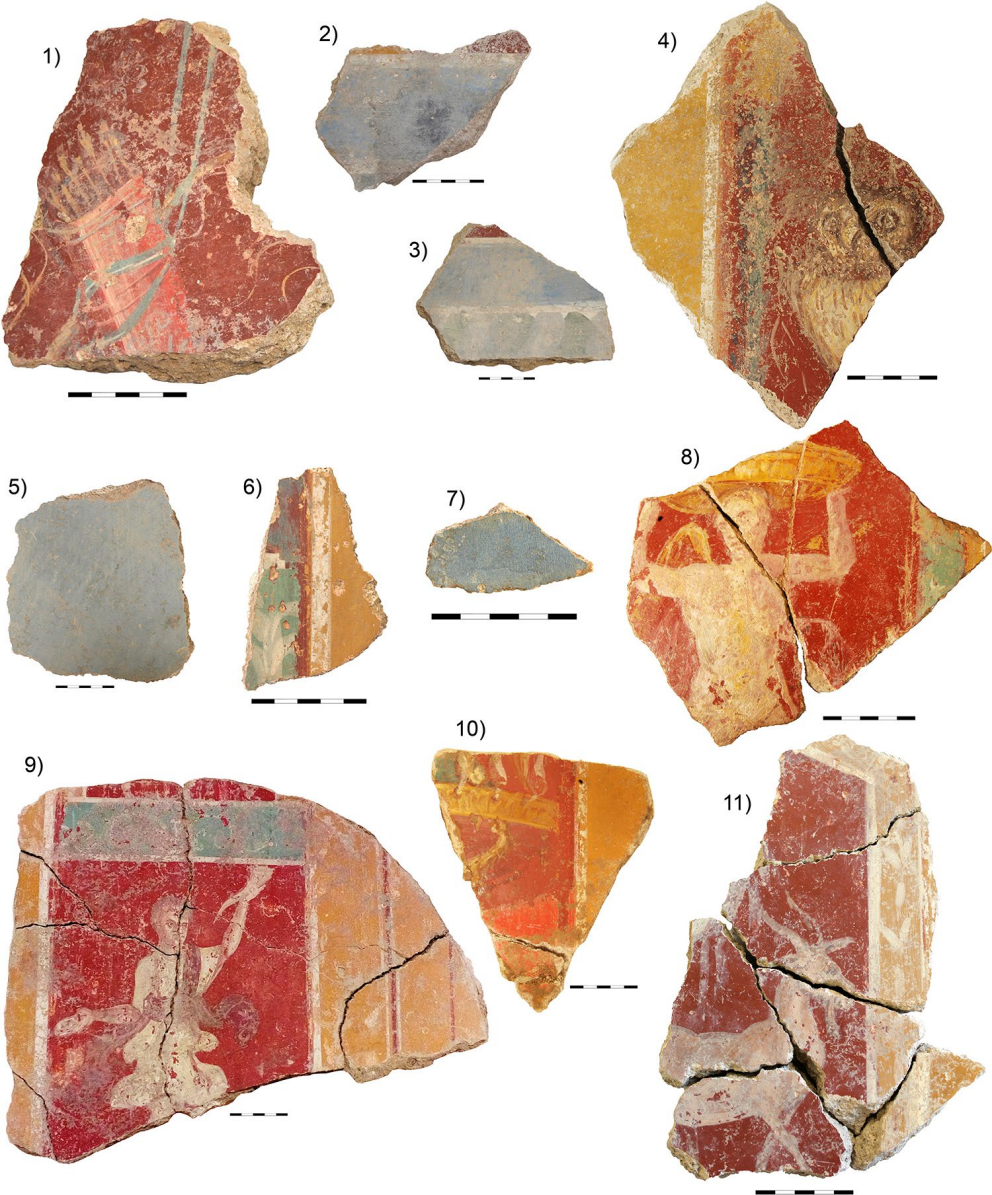
Analysed fragments of Roman wall painting from room 6.

The samples under study evidence other major points in pigment analysis: complex figurative decoration, mixed colours, overlapping colour layers of varying thickness (colour stratigraphy is especially relevant, as it may bias the results), and the use of pigments that must have had a major influence on the entire process of painting (Fig. 2).

## Results

**Figure 3:**
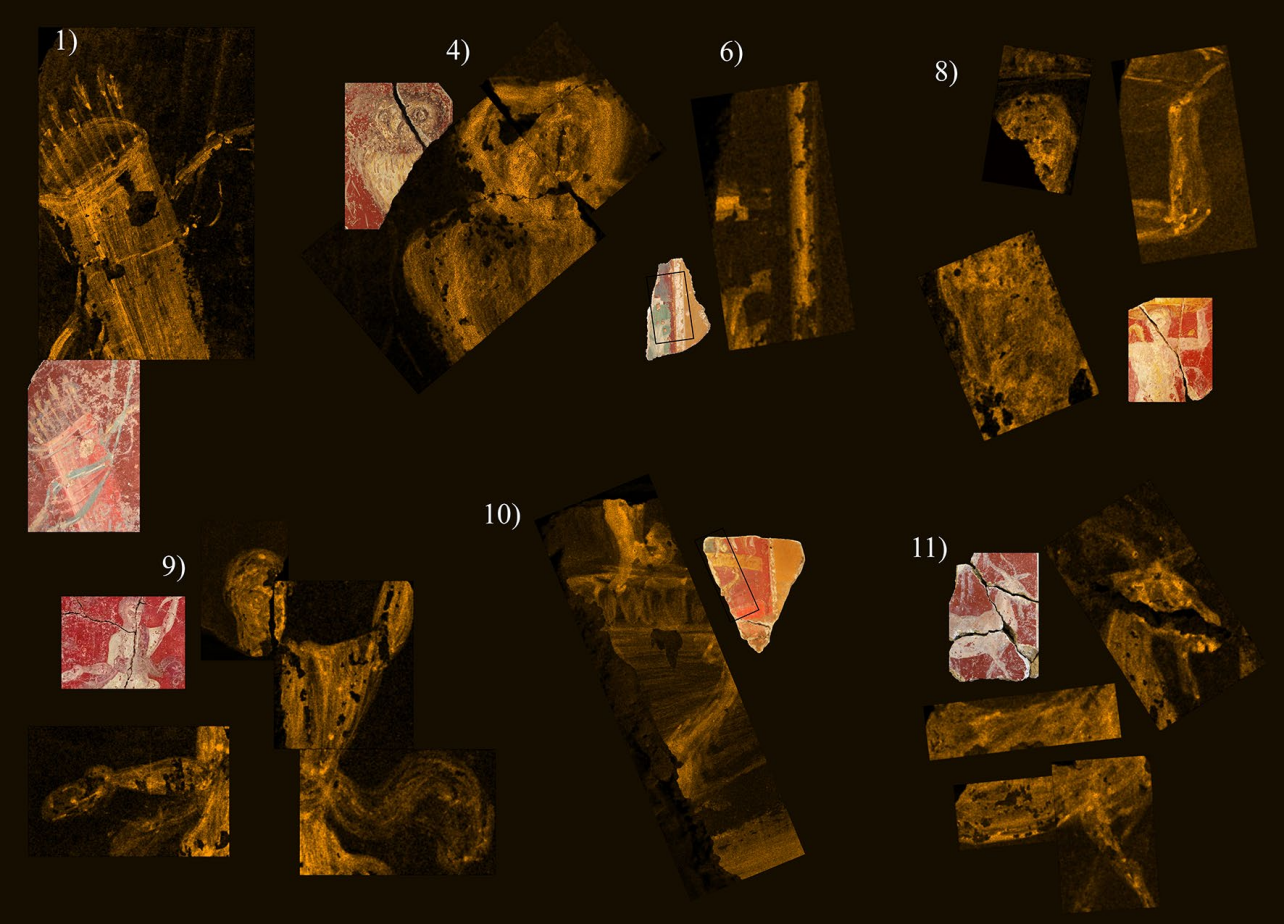
µEDXRF mappings of strontium (Sr) in fragments 1, 4, 6, 8, 9, 10 and 11. Experimental conditions in Supplementary Table S15.

A differential overview of the composition of clothes, bodies and areas decorated with white lines compared with the coloured backgrounds was obtained by µEDXRF scanning. This technique detected a relative concentration of Sr in the surface of the fragments with figurative decoration three times higher than in the red and yellow backgrounds (Fig. 3, Table 1). The same can be said of the white lines that separate surfaces of different colour in the fragments with non-figurative decoration (fragment 6). The conclusion is that the decoration was made with pigments with a common base, where the amount of Sr was significant. This may be because strontianite (SrCO_3_) was used alongside calcite (CaCO_2_) for some pigments^14,32,33^.

**Table 1:** μEDXRF single-spot analyses

Colour	Frag	a/b	nº pt	Na_2_O	s.d	MgO	s.d	Al_2_O_3_	s.d	SO_3_	s.d	K_2_O	s.d	CaO	s.d	Fe_2_O_3_	s.d	CuO	s.d	SrO	s.d	HgO	s.d	PbO_2_	s.d
Yellow	1	b	5	0.01	0.03	1.16	0.89	2.16	0.89	1.02	0.24	0.60	0.19	67.43	10.58	16.55	8.81	0.02	0.01	0.54	0.25	0.00	0.00	0.59	0.32
	2	a	1	0.00	–	0.87	–	6.10	–	0.13	–	0.89	–	52.50	–	22.95	–	0.02	–	0.17	–	0.00	–	0.08	–
	4	a	3	0.03	0.03	0.89	0.19	5.29	0.71	0.30	0.23	0.99	0.10	40.98	3.08	30.63	4.26	0.02	0.00	0.25	0.08	0.00	0.00	0.05	0.01
	4 Fe	b	4	0.32	0.22	0.85	0.39	2.53	0.93	0.30	0.04	0.46	0.37	73.43	5.12	9.13	4.79	0.03	0.01	1.00	0.72	0.00	0.00	0.38	0.03
	4 Pb	b	3	0.68	0.14	0.85	0.22	2.10	1.17	0.27	0.08	0.31	0.23	77.08	7.38	4.43	2.73	0.03	0.01	1.97	0.50	0.00	0.00	1.64	0.63
	6	a	3	0.04	0.07	1.31	0.27	6.04	0.56	0.11	0.05	1.00	0.14	42.12	5.79	26.51	6.58	0.03	0.01	0.11	0.01	0.00	0.00	0.08	0.02
	8	a	1	0.00	–	0.80	–	5.23	–	0.03	–	0.97	–	40.25	–	26.20	–	0.03	–	0.12	–	0.00	–	0.11	–
	8	b	6	0.00	0.00	1.11	0.61	2.31	0.78	0.11	0.07	0.32	0.10	63.22	13.29	23.16	12.67	0.28	0.30	0.14	0.58	0.00	0.00	0.30	0.13
	9	a	5	1.19	2.27	1.64	0.08	6.83	0.78	0.00	0.00	0.91	0.10	43.50	4.44	23.22	6.44	0.02	0.01	0.11	0.04	0.00	0.00	0.06	0.03
	10	a	3	0.00	0.00	0.61	0.34	4.14	0.34	0.48	0.10	1.04	0.13	55.03	3.00	22.52	3.00	0.02	0.01	0.16	0.02	0.00	0.00	0.09	0.01
	10	b	3	0.00	0.00	1.01	0.06	3.24	0.61	0.58	0.07	0.73	0.24	65.05	6.52	15.38	3.62	0.29	0.14	0.12	0.26	0.00	0.00	0.45	0.24
	11	a	5	0.00	0.00	1.78	0.21	5.63	2.15	0.33	0.02	0.64	0.53	47.32	7.54	27.57	3.73	0.04	0.02	0.12	0.03	0.00	0.00	0.48	0.24
	11 Fe	b	19	0.00	0.02	2.01	0.42	4.70	0.99	0.49	0.06	0.57	0.15	56.89	8.08	18.86	6.90	0.12	0.18	0.25	0.22	0.00	0.00	0.46	0.19
	11 Pb	b	2	0.00	0.00	1.88	0.11	3.80	0.17	0.47	0.12	0.78	0.33	63.66	1.94	14.11	1.36	0.03	0.00	0.64	0.07	0.00	0.00	1.05	0.07
Red	1	a	4	0.05	0.07	1.39	0.30	1.63	0.45	0.64	0.16	0.32	0.03	44.45	7.91	44.73	6.73	0.06	0.02	0.18	0.02	0.01	0.01	0.11	0.02
	1	b	6	0.00	0.00	1.54	0.27	3.63	1.33	5.27	4.08	0.60	0.17	44.86	14.45	15.04	9.67	0.11	0.09	0.29	0.18	14.43	15.19	1.20	0.71
	2	a	1	0.00	–	2.24	–	1.79	–	0.12	–	0.24	–	49.27	–	38.50	–	0.06	–	0.20	–	0.00	–	0.07	–
	3	a	2	0.10	0.13	2.16	0.52	2.76	0.33	0.19	0.04	0.30	0.04	41.17	12.66	43.69	15.12	0.04	0.00	0.15	0.07	0.00	0.00	0.07	0.00
	4	a	5	0.19	0.11	1.51	0.58	2.21	0.98	0.26	0.05	0.45	0.16	52.93	6.48	30.68	8.18	0.09	0.11	0.19	0.07	0.00	0.00	0.09	0.02
	6	a	3	0.05	0.09	2.24	0.55	3.29	0.72	0.20	0.03	0.69	0.31	45.59	11.94	27.45	3.99	0.29	0.46	0.13	0.02	0.00	0.00	0.11	0.02
	8	a	2	0.00	0.00	0.86	0.37	1.49	0.53	0.11	0.02	0.33	0.09	50.45	4.33	39.86	1.97	0.08	0.04	0.17	0.02	0.00	0.00	0.14	0.01
	9	a	6	2.11	3.26	1.82	0.37	2.23	0.50	0.00	0.00	0.26	0.05	43.23	11.58	38.17	15.23	0.05	0.03	0.11	0.04	0.07	0.15	0.08	0.02
	9	b	7	0.05	0.10	1.92	0.64	3.16	0.76	0.12	0.09	0.36	0.09	63.11	5.86	17.90	4.51	0.13	0.09	0.50	0.19	0.01	0.03	0.20	0.16
	10	a	3	0.05	0.09	0.89	0.10	1.54	0.27	0.70	0.16	0.40	0.04	42.87	4.47	45.89	4.32	0.09	0.03	0.13	0.02	0.01	0.01	0.13	0.04
	10	b	2	0.00	0.00	1.28	0.20	4.95	0.34	0.54	0.02	1.23	0.33	48.14	5.23	15.55	2.01	0.37	0.04	0.39	0.01	0.33	0.11	0.18	0.20
	11	a	7	0.00	0.00	2.14	0.19	3.46	0.46	0.49	0.07	0.44	0.14	42.43	10.51	38.74	11.22	0.22	0.35	0.13	0.03	0.00	0.01	0.14	0.15
	11	b	7	0.00	0.00	2.39	0.53	4.13	0.39	0.51	0.09	0.52	0.17	55.22	7.69	21.69	7.35	0.18	0.21	0.52	0.30	0.00	0.00	0.67	0.48
Brown	4	b	12	0.30	0.38	1.14	0.46	2.51	0.64	0.35	0.12	0.53	0.14	55.83	9.24	21.09	9.82	0.14	0.13	1.03	0.28	0.00	0.00	0.46	0.19
	9	b	3	0.00	0.00	1.74	0.40	2.66	0.74	0.18	0.07	0.26	0.06	74.99	5.16	6.29	3.26	0.05	0.04	1.19	0.35	3.09	1.58	0.18	0.03
Pink	1	b	8	0.00	0.00	1.52	0.25	4.03	1.17	8.15	5.08	0.51	0.20	45.16	16.83	14.77	7.53	0.04	0.02	0.34	0.18	11.07	9.68	1.74	0.82
	9	b	9	0.00	0.00	1.69	0.25	2.98	1.08	0.45	0.48	0.41	0.37	70.75	8.24	7.96	4.08	0.05	0.04	0.82	0.40	2.54	2.56	0.17	0.08
	10	b	3	0.00	0.00	0.98	0.16	2.68	0.85	8.16	1.65	0.54	0.17	54.41	7.06	4.34	1.06	0.05	0.01	1.05	0.11	14.30	3.89	3.93	0.61
Blue	1	b	5	0.16	0.25	1.50	0.11	3.99	0.26	0.26	0.12	1.34	0.34	46.73	5.46	3.53	1.53	3.28	2.03	0.36	0.11	11.01	6.66	1.61	0.48
	2	b	2	0.89	0.34	1.71	0.48	2.99	0.30	0.06	0.08	0.52	0.12	25.71	8.60	0.85	0.37	10.95	11.38	0.23	0.21	0.00	0.00	0.03	0.01
	3	b	2	1.80	2.10	1.79	1.16	5.09	3.37	0.12	0.08	1.22	0.50	26.22	11.91	1.29	0.88	9.86	7.33	0.17	0.02	0.00	0.00	0.06	0.01
	5	a/b	6	0.41	0.57	1.40	0.91	4.86	3.60	0.37	0.22	1.30	0.93	27.72	9.11	1.60	1.13	15.01	6.51	0.07	0.02	0.00	0.00	0.14	0.04
	6	b	2	0.88	0.28	1.88	0.21	3.33	0.06	0.16	0.02	2.07	1.28	28.42	5.19	10.12	2.08	9.47	2.88	0.13	0.07	0.00	0.00	0.08	0.01
	7	a/b	4	0.45	0.51	1.02	0.73	4.51	2.48	0.56	0.25	1.22	0.73	22.15	6.67	1.40	1.01	14.64	7.11	0.11	0.04	0.00	0.00	0.14	0.03
Green	1	b	4	0.00	0.00	2.85	0.28	4.50	0.64	0.06	0.11	4.47	0.35	41.70	3.96	16.66	6.59	0.07	0.03	0.19	0.10	3.18	3.72	0.74	0.84
	1 (b.n.)	b	1	1.28	–	2.19	–	5.75	–	0.09	–	3.72	–	30.65	–	12.01	–	7.17	–	0.12	–	0.00	–	0.04	–
	2	b	1	0.78	–	2.21	–	4.32	–	0.15	–	1.93	–	62.88	–	3.74	–	0.34	–	0.09	–	0.00	–	0.05	–
	3	b	3	0.00	0.00	3.01	1.49	4.73	1.48	0.14	0.03	5.38	3.85	40.28	27.39	11.11	7.37	0.26	0.15	0.14	0.01	0.00	0.00	0.06	0.01
	4	b	10	0.31	0.38	1.78	0.61	3.58	1.03	0.20	0.05	3.01	2.00	40.03	13.94	18.45	6.97	0.09	0.14	0.19	0.06	0.00	0.00	0.07	0.04
	4 (b.n.)	b	1	0.66	–	0.96	–	2.71	–	0.22	–	3.18	–	43.59	–	16.68	–	4.05	–	0.19	–	0.00	–	0.09	–
	6	b	3	0.00	0.00	2.26	1.06	4.59	1.04	0.12	0.13	3.82	0.99	38.98	2.77	20.11	3.14	0.03	0.01	0.10	0.02	0.00	0.00	0.10	0.01
	6 (b.n.)	b	1	0.00	–	3.33	–	3.35	–	0.26	–	3.72	–	24.03	–	19.59	–	1.00	–	0.11	–	0.00	–	0.12	–
	8	b	1	0.00	–	2.21	–	3.38	–	0.02	–	4.07	–	39.19	–	23.59	–	1.78	–	0.13	–	0.00	–	0.14	–
	8 (b.n.)	b	2	0.35	0.06	0.71	0.08	1.71	0.10	0.01	0.01	1.07	0.19	25.19	1.90	15.09	1.07	11.96	1.20	0.06	0.00	0.00	0.00	0.09	0.00
	9	b	5	3.71	1.77	1.94	0.43	5.04	1.86	0.04	0.09	2.68	0.41	34.95	7.84	13.93	4.20	0.23	0.11	0.11	0.03	0.01	0.01	0.13	0.18
	9 (b.n.)	b	6	2.00	0.86	2.19	0.30	3.09	0.61	0.00	0.00	2.42	0.44	30.93	3.38	16.50	9.29	6.38	3.69	0.16	0.03	0.00	0.01	0.05	0.01
	10	b	4	0.00	0.00	2.02	0.39	4.71	1.50	0.44	0.06	3.22	0.71	41.70	5.58	18.55	6.41	1.06	0.88	0.17	0.05	0.01	0.01	0.09	0.01
	10 (b.n.)	b	3	0.73	0.56	0.94	0.13	2.96	0.53	0.30	0.05	1.83	0.81	25.06	5.06	10.31	9.15	14.20	6.17	0.14	0.06	0.01	0.01	0.06	0.01
White	1	b	4	0.00	0.00	1.36	0.38	2.62	0.86	1.50	0.47	0.61	0.46	67.34	6.03	7.38	4.98	0.03	0.03	0.68	0.30	7.16	4.77	2.16	1.01
	2	b	2	0.48	0.42	0.88	0.10	1.30	0.25	0.26	0.04	0.22	0.06	85.07	6.13	4.94	5.03	0.26	0.03	0.95	0.05	0.00	0.00	0.06	0.01
	2 (b.n.)	b	3	0.76	0.76	1.17	0.41	3.46	2.50	0.82	0.13	0.61	0.38	61.95	6.10	2.65	2.12	6.40	1.74	0.59	0.17	0.00	0.00	0.03	0.02
	3	b	2	0.00	0.00	1.65	0.46	3.11	0.21	0.26	0.02	0.24	0.01	81.87	0.63	1.94	0.99	0.10	0.10	0.69	0.71	0.00	0.00	0.11	0.04
	3 (b.n.)	b	2	0.09	0.13	1.84	0.72	2.63	0.21	0.84	0.01	0.36	0.10	67.51	17.16	2.84	2.96	4.69	4.18	0.26	0.33	0.00	0.00	0.03	0.01
	4	b	5	0.60	0.32	0.74	0.22	1.81	0.33	0.32	0.06	0.36	0.11	80.79	1.51	4.62	1.51	0.06	0.05	1.05	0.32	0.00	0.00	0.22	0.14
	4 (b.n.)	b	3	0.73	0.31	0.91	0.42	1.40	0.15	0.84	0.06	0.41	0.02	55.80	5.50	2.52	1.17	10.59	4.50	0.58	0.04	0.00	0.00	0.12	0.14
	6	b	5	0.02	0.04	1.24	0.45	2.17	0.51	0.26	0.03	0.32	0.09	78.54	5.61	5.47	4.32	0.04	0.02	1.01	0.46	0.00	0.00	0.10	0.03
	6 (b.n.)	b	1	0.04	–	0.74	–	2.34	–	0.23	–	0.64	–	59.85	–	1.38	–	8.54	–	1.37	–	0.00	–	0.13	–
	8	b	5	0.00	0.00	1.10	0.36	3.93	1.75	0.18	0.08	0.55	0.27	70.41	12.14	9.74	6.23	0.14	0.12	0.72	0.30	0.00	0.00	0.19	0.09
	8 (b.n.)	b	2	0.07	0.10	0.32	0.21	1.76	0.40	0.25	0.01	0.50	0.06	51.13	14.06	6.30	6.56	13.98	11.53	0.96	0.25	0.00	0.00	0.20	0.08
	9	b	9	0.68	1.27	1.69	0.43	3.56	2.15	0.18	0.08	0.54	0.38	71.37	10.11	6.86	3.81	0.10	0.13	0.75	0.28	0.04	0.06	0.08	0.04
	9 (b.n.)	b	2	0.59	0.58	1.48	0.61	4.67	1.38	0.12	0.10	0.63	0.16	35.48	16.84	1.81	0.34	13.93	7.69	0.72	0.13	0.00	0.00	0.07	0.01
	10	b	5	0.01	0.03	0.77	0.17	2.07	0.81	0.62	0.06	0.53	0.21	76.52	10.70	6.71	3.76	0.13	0.10	0.76	0.17	0.01	0.02	0.11	0.05
	11	b	9	0.00	0.00	1.90	0.37	4.95	1.71	0.42	0.11	0.72	0.45	62.29	5.95	13.08	2.21	0.11	0.21	0.39	0.06	0.01	0.01	0.26	0.20
	11 (b.n.)	b	6	0.20	0.28	1.43	0.22	3.63	0.75	0.39	0.05	0.47	0.04	51.21	10.21	13.38	7.34	5.45	3.33	0.29	0.07	0.00	0.01	0.27	0.18

Average values. a: on background; b: on motives; nº pt: number of single-spot analyses in different positions per fragment; s.d.: standard deviation; b.n.: blue nodule.

MRS failed to detect the presence of Sr. This fact can find a two-fold explanation: (1) the position of the symmetric carbonate stretching vibration in group II carbonates is controlled by the presence of calcium, even when significant amounts of strontium or barium occur in the crystalline structure^34^^,^ (2) the detected relative amounts of strontium are very low (0.5 wt % on average) as compared with calcium (57.31 wt % on average). Thus, this is a case in point as regards the relevance and the descriptive potential of surface composition scanning (µEDXRF): the occurrence and specific distribution of strontium would have not been recorded otherwise, and its role in the decoration would have passed unnoticed for the single-spot method.

On the other hand, in some previous studies^35,36^, the presence of Sr was connected with the use of gypsum or celestine in the pictorial and/or preparation layers. In the case of our samples, the lacking evidence of these in any of the Raman spectra in the samples under research and the low relative concentrations of sulphur attested by µEDXRF (0.35 wt % on average) suggest that the use of these species should be discarded. As a matter of fact, relevant levels of S were detected only in fragments 1 and 10, but even in these cases, the values are most probably due to the cinnabar used as a pigment for the pink layer under the white motifs researched.

An alternative hypothesis that should be dis/proved in subsequent research based on a larger number of samples bears relation to the protective and luminescent properties of Sr^37,38^: as it occurred only in the samples of figures and in white lines, it may have been used intentionally to give the main scenes a brighter shade and, therefore, higher relevance.

### The colours of the palette

**Figure 4:**
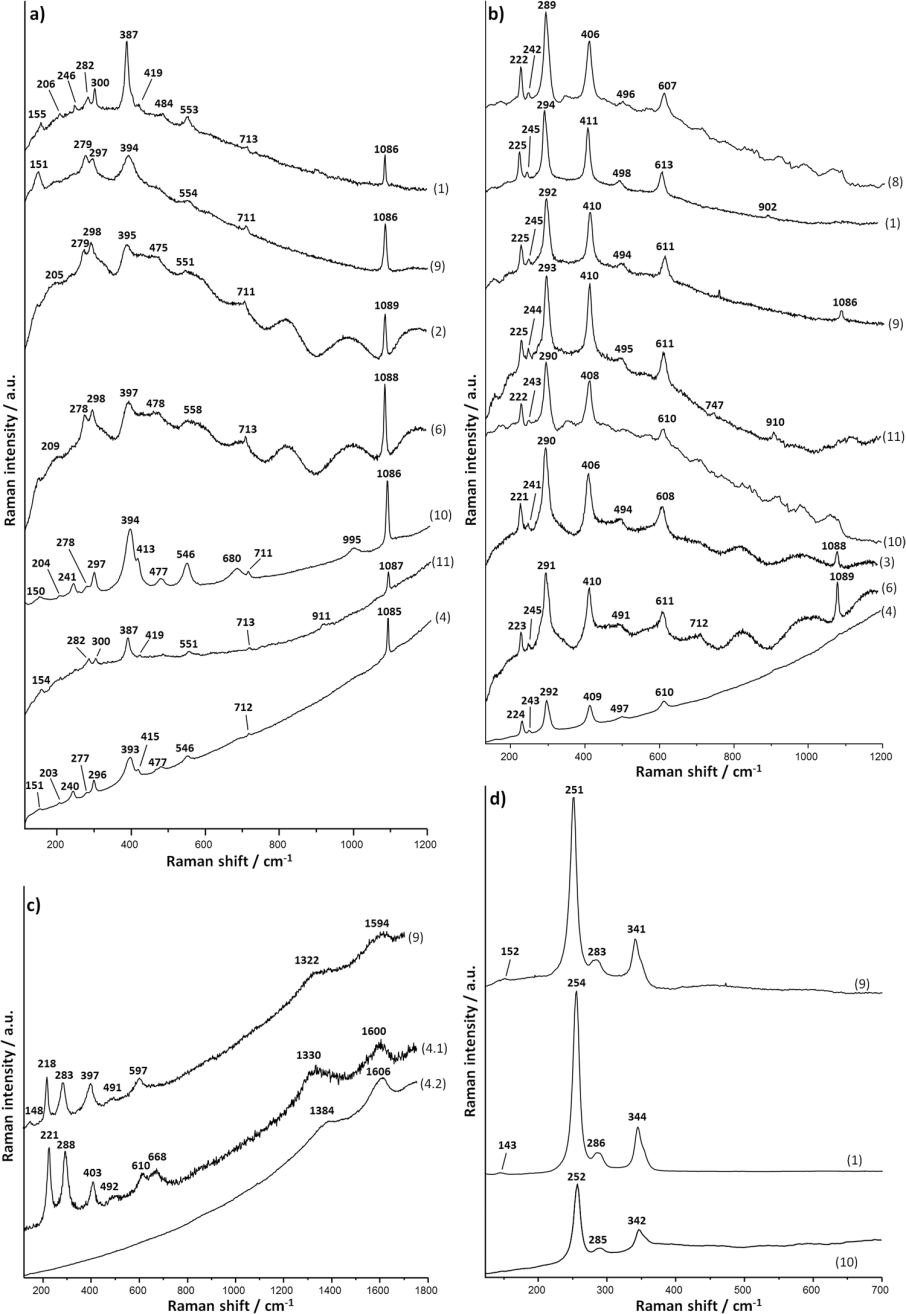
MRS results obtained in fragments 1 to 11. (**a**) Raman spectra of yellow decoration in fragments 1, 2, 4, 6, 9, 10 and 11 (Goethite). (**b**) Raman spectra of red decoration in fragments 1, 3, 4, 6, 8, 9, 10 and 11 (Hematite). (**c**) Raman spectra of brown decoration in fragments 4 and 9 (Hematite and amorphous carbon). (**d**) Raman spectra of pink decoration in fragments 1, 9 and 10 (Cinnabar). Experimental conditions in Supplementary Table S13.

**Figure 5:**
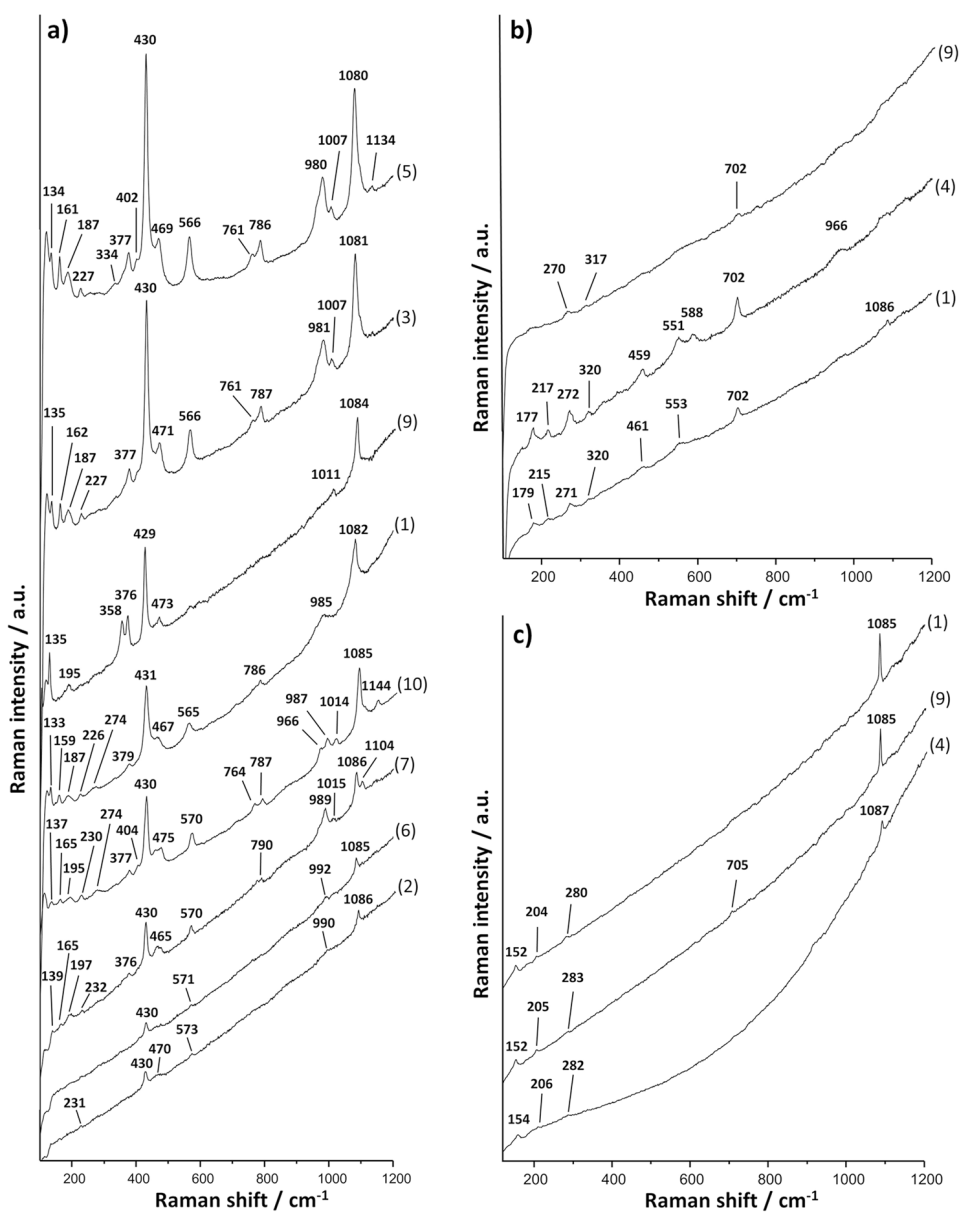
MRS results obtained in fragments 1 to 11. (**a**) Raman spectra of blue decoration in fragments 1, 2, 3, 5, 6, 7, an of blue nodules in green fragments 9 and 10 (Egyptian blue). (**b**) Raman spectra of green decoration in fragments 1, 4 and 9 (Celadonite/glauconite). (**c**) Raman spectra of white decoration in fragments 1, 4 and 9 (Calcite). Experimental conditions in Supplementary Table S13.

Supplementary Table S1 shows the Raman bands of the spectra that were considered significant and that led to the diagnosis of the mineral components of the pigments used. Figures 4 and 5 show the most significant Raman spectral profiles and the average concentration values of elements identified by μEDXRF (Table 1) and that may be of relevance for the disclosure of the mineral composition of the decorated areas (Supplementary Tables S2–S12).

#### Yellow

Yellow was recorded in 8 fragments (1, 2, 4, 6, 8, 9, 10 and 11). Two types were found: Fe-yellow and Pb-yellow.

The former occurred in the backgrounds of the main scenes and in various parts of figurative motifs: the arrows in the quiver (fragment 1), the feathers and the head of the Little owl (fragment 4), and the object that the human figure carries on its head (fragment 8). The Raman spectra obtained are characteristic of goethite^39^ (α-FeO(OH), even if a band characteristic of calcite (Fig. 4a^,^Supplementary Table S1) was also obtained around 1085 cm^−1^. Calcite may have been detected for being a component of the pigment, or because the pigment was applied directly on a wall with some content in calcite. The concentrations of Fe in the yellow parts of the fragments under research ranged between 9.13 wt% in the Little owl (fragment 4) and 30.63 wt% of the yellow background of the same fragment (Table 1).

**Figure 6:**
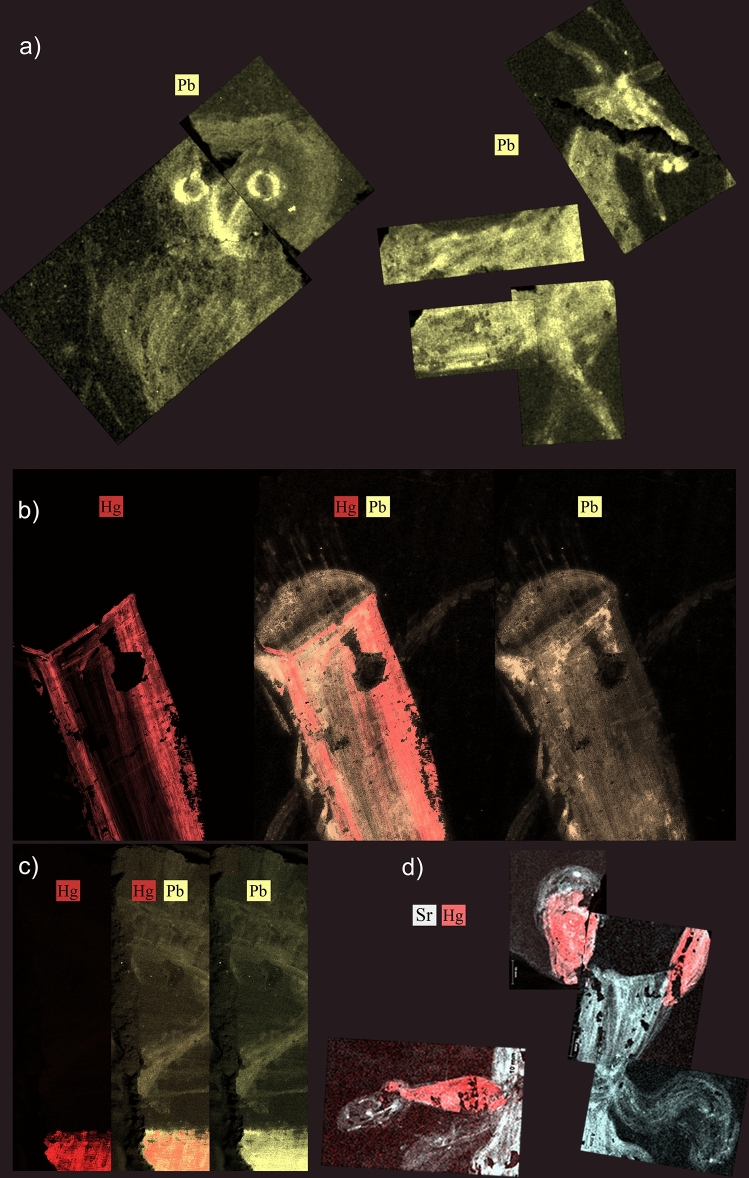
μEDXRF mappings. (**a**) Lead (Pb) in fragment 4 (Little owl) and fragment 11 (goat). (**b**) Mercury (Hg) and lead (Pb) in fragment 1 (quiver). (**c**) Mercury (Hg) and lead (Pb) in fragment 10. (**d**) Strontium (Sr) and lead (Hg) in fragment 9 (Lar). Experimental conditions in Supplementary Table S15.

The results obtained from µEDXRF analysis, including the maps of surface composition, identified specific areas where a second type of yellow pigment with a significant amount of Pb (*ca*. 1 wt%) was used specifically in the beak and the eyes of the Little owl (fragment 4) and in the body of the goat (fragment 11) (Fig. 6a^,^Table 1). This colour effect is hardly noticeable both for the surface decay (Raman spectra of Pb-based compounds could not be obtained) and for the small size of the figures. Still, the distribution maps of Pb clearly showed that, at least as regards the Little owl, the intention was to reproduce the typical shade of yellow of the beak and the sclerotic of the eye.

According to classical sources like Theophrastus, Vitruvius and Pliny the Elder^40–42^, the yellow pigment may have been obtained heating lead white or *cerussa* ((PbCO_3_)_2_·Pb(OH)_2_) after submersion of lead in vinegar. The resulting lead white then transformed into various types of lead oxides in shades of yellow, fawn and red according to the time and temperature at which it was heated^43–45^.

#### Red

Red was recorded in most samples (fragments 1, 2, 3, 4, 6, 8, 9, 10 and 11). In general, a uniform shade of red was used as background of the decoration panels, and a range of reds occurred in figurative details (samples 1, 8, 9, 10 and 11) like the flesh, the cloth, and physical details of animals.

The Raman spectra obtained evidenced that the pigment was made with hematite^39^ (α-Fe_2_O_3_) (Fig. 4b) (Supplementary Table S1). The µEDXRF results attested high or very high concentrations of Fe between 15.04 wt% in fragment 1 and 45.89 wt% in fragment 10 (Table 1). This evidenced the robust and sturdy background layer of red too.

#### Brown

Brown was recorded in the delineation of the head and the body of the Little owl (fragment 4) and in the head of the Lar (fragment 9). The Raman spectra obtained there (Fig. 4c) suggest that the colour shade resulted from hematite red darkened with amorphous carbon (G band,1550 to 1620 cm^−1^, and D band 1301-1340 cm^−1^)^46^ (Supplementary Table S1). The µEDXRF data confirmed that Fe prevails in these areas (Table 1), so other possible sources for this shade were discarded. This combination of hematite and amorphous carbon has been found not only in the decoration of Roman contexts^47^ but also, for example, in Etruscan tombs^48^ and in Egyptian tombs of the 2nd ct. a.D.^49^.

Amorphous carbon was not identified in any of the remaining areas and motifs where hematite was present and detected using similar (and even higher) laser powers (Supplementary Table S13). Therefore, the thermal degradation of the samples is an unlikely reason for the occurrence of carbon.

The interpretation of the vibrational band at 668 cm^-1^ (Fig. 4c, Raman spectrum 4.1) alongside the typical spectral bands of hematite is controversial in the literature. It has been put down to the occurrence of magnetite^50^ and, therefore, to a darkened red pigment^51–53^, but it has also been attributed to a disorder-activated mode in the hematite crystalline structure^54,55^. The occurrence of this element in only one sample and the attestation of amorphous carbon in the rest of the brown samples suggests that magnetite was not used as a darkening agent.

#### Pink

Various tones of pink are noticeable in the quiver (fragment 1), in the face and in the arms of the Lar (fragment 9), and also on a smooth, shapeless surface of fragment 10.

MRS analysis of all these areas revealed the characteristic spectrum of cinnabar^56^ (HgS), known as *minium* by the Romans (Fig. 4d, Supplementary Table S1). µEDXRF analysis confirmed the above based on the relative high concentrations of Hg, ranging from 2.54 to 14.30 wt% according to the thickness of the layer (Table 1). µEDXRF scanning of elemental composition contributed further details: Hg and Pb were detected in fragments 1 and 10 (Fig. 6b and 6c), the former showing lower relative concentrations of Pb (1.74 wt %) than the latter (3.93 wt%) (Table 1).

The most plausible explanation for this combination is the mix of specific proportions of cinnabar and a lead-based pigment (as described above) in order to obtain a specific colour shade, as in the quiver (Fragment 1): while Pb and Hg were recorded over virtually all the surface, only Pb was recorded in the left and the upper parts. This supports the hypothesis of the use of two pigments of different shades, each made of different base elements.

Cinnabar was frequently mixed with hematite or with lead-base compounds in ancient times both to save on the then highly exclusive cinnabar and to obtain specific shades. These combinations were described by Pliny the Elder^42^^,^ but are present as early as in the Neolithic^57,58^. Its use is, however, particularly remarkable in Roman times in such relevant sites as *Domus Aurea*, Pompeii, and Herculaneum^59–62^. Cinnabar was used in Cástulo both alone and mixed with other minerals or pigments, as is natural considering both the high standing of the building and the proximity to the mines of Almadén (in the neighbouring province of Ciudad Real).

Also, Pb was not detected in the decoration of the Lar (fragment 9), and cinnabar was identified only in the face and in the arm, probably intended to highlight the colour of the flesh (Fig. 6d). Again, Pliny the Elder wrote in his work *Natural History*: “… I am quite at a loss for the origin of this usage; but it is a well-known fact, that at the present day even, minimum is in great esteem with the nations of Æthiopia, their nobles being in the habit of staining the body all over with it, and this being the colour appropriated to the statues of their gods”^42^.

#### Blue

Egyptian blue (CaCuSi_4_O_10_) was the main pigment for this colour. Egyptian blue, also known as cuprorivaite, is a synthetic compound: a calcium-copper tetrasilicate, obtained by heating a mixture composed of calcite, siliceous sand, copper compounds, and natron or plant ash, to a temperature ranging between 850 and 950°C^1,56^.

**Figure 7:**
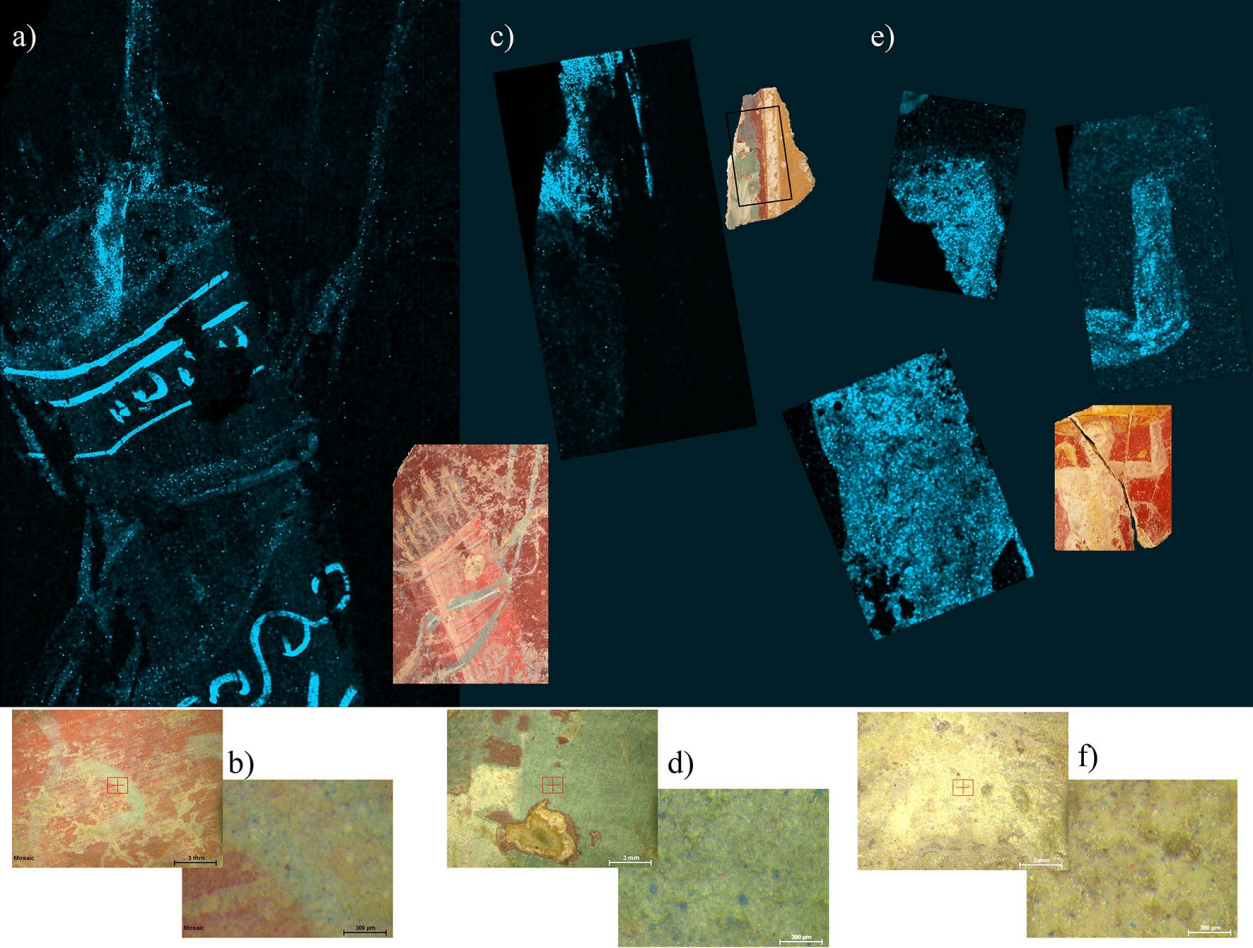
(**a**) μEDXRF mapping of copper (Cu) in fragment 1 (quiver). (**b**) Details of blue decoration on fragment 1. (**c**) μEDXRF mapping of copper (Cu) in fragment 6. (**d**) Details of green decoration with blue nodules on fragment 6. (**e**) μEDXRF mapping of copper (Cu) in fragment 8. (**f**) Details of white decoration with blue nodules on fragment 8. Experimental conditions in Supplementary Table S15.

Egyptian blue was detected by MRS analysis in the pigments of the decorations of 6 fragments (1, 2, 3, 5, 6 and 7) (Fig. 5a, Supplementary Table S1). µEDXRF analysis confirmed a differential occurrence of Si, Ca and Cu in the blue-coloured areas (Table 1). The results of the quiver (fragment 1) are worth special note: blue was detected by µEDXRF surface scanning, even if it is barely visible. The image obtained showed a differential concentration of Cu, making decoration by straight and wavy lines more visible on the surface of the quiver (Fig. 7a,b).

#### Green

Eight samples with shades of green were analysed (fragments 1, 2, 3, 4, 6, 8, 9 and 10). A common source for green in Roman times was the so-called green earth (*Creta viridis* or *appianum* in Vitruvius and Pliny)^41, 42^, which covers two phyllosilicates of the mica family: celadonite K(MgFe^2+^)(Fe^3+^,Al)Si_4_O_10_(OH)_2_ and glauconite (K,Na)(Fe^3+^,Al,Mg)_2_(Si,Al)_4_O_10_(OH)_2_. Difficult to identify, both have complex chemical structures and their composition, crystalline structure, morphology, and origin may vary widely: pure celadonite occurs in amygdules or fractures of metamorphic rocks, whereas glauconite occurs as small green lumps (green sand) in shallow marine sedimentary rocks^59,63,64^.

Raman spectroscopy has been used repeatedly to set criteria for the separation of both minerals in research on the source of green colour in Roman paintings. Celadonite has been proved to display two bands at ∼174 cm^−1^ and ∼202 cm^−1^, whereas glauconite displays only one band: between 188 and 200 cm^−1^ (perhaps an unresolved multiplet)^29,63–65^. Also, the medium-high intensity band around 265 cm^−1^ has a higher wavenumber in celadonite (272–279 cm^−1^) than in glauconite (263 to 266 cm^−1^). Another difference is that the band in the 380–400 cm^−1^ region of celadonite (394–400 cm^−1^) is displaced compared with the one of glauconite (384 to 389 cm^−1^).

Two criteria can be used as regards the elemental composition: Na is a relevant component only in glauconite, (impurities may cause traces or low amounts to occur in celadonite)^65^^,^ and the proportion Si:Al:Mg is different too: Si > Al > Mg signals the occurrence of glauconite, whereas Si > Mg > Al signals the occurrence of celadonite^64^.

Despite intrinsic difficulties for their differentiation, the Raman spectra obtained in the green-coloured areas of fragments 1 and 4 established the occurrence of celadonite (Fig. 5b, Supplementary Table S1). This was confirmed further by µEDXRF’s attestation of low amounts of Na in the decorations (Table 1). By contrast, the Raman bands and the relative content of Na (Table 1) of fragment 9 pointed at glauconite as the source of green colour (Fig. 5b^,^Supplementary Table S1).

Although the Raman spectra of the green areas of fragments 2, 6, 8 and 10 did not give significant results, the low or non-existent concentrations of Na may be taken as evidence of the occurrence of celadonite (Table 1).

Alternatively, according to the proportion Si:Al:Mg alone, glauconite occurs in all the samples. This conclusion, however, runs against the evidence of samples with low or not any Na contents, and is wrong for the samples where Raman analysis enabled the detection of celadonite.

Regarding fragments 1, 4, 6, 8, 9 and 10, microscopic inspection of green areas revealed the occurrence of deep blue nodules, specifically Egyptian blue, according to MRS analysis (Supplementary Table S1). This artificial pigment was a common resource to obtain green earth of a special hue^1,29,65^. The occurrence of Egyptian blue in these 6 fragments (maybe intended for a different shade from that of green earth), but not in the other 2 (fragments 2 and 3) was clearly evidenced by surface scanning of Cu (Fig. 7c,d, Table 1).

All in all, two types of green tones can be separated: one obtained exclusively from green earth (fragments 2 and 3), and one obtained from green earth mixed with Egyptian blue (fragments 1, 4, 6, 8, 9 and 10).

#### White

White was recorded in decorative bands on red or yellow background, in vegetable motifs and in some other figurative motifs of fragments 1, 2, 3, 4, 6, 8, 9, 10 and 11. The Raman spectra obtained evidenced calcite in all these fragments^66^ (Fig. 5c, Supplementary Table S1). This was supported by µEDXRF attestation of high relative amounts of Ca in all the samples (Table 1).

Despite the seeming homogeneity of all the samples, fragments 2, 3, 4, 6, 8, 9 and 11 diverged as regards the composition of white areas. Microscopic inspection and µEDXRF single-spot and surface analysis allowed visualization and analysis of blue nodules high in Cu (Table 1). Egyptian blue mixed with calcite for a brighter shade of white, as is especially noticeable in the figure of fragment 11 (Fig. 7e,f), thus became well-reasoned conclusion.

Joint use of spectroscopic methods (MRS, µEDXRF) for research on the compounds used for the pigments of the decorations in the Roman wall paintings of Cástulo proved highly effective and may be relevant for other research projects. The possibility to work on small fragments thanks to the abandonment conditions of the site solved the awkwardness and difficulty of on-site colour analysis.

Notably, µEDXRF surface analysis improves the quality and significance of the results expected using exclusively MRS and µEDXRF single-spot techniques. This paper actually is, in a way, a protocol for research on decorations where the first step can be µEDXRF surface analysis, at least in the areas where overlapping, thick, mixed or degraded colours make research particularly difficult. Accurate identification of the raw materials used and of their concentrations still needs MRS and µEDXRF single-spot analysis, except that at such a later stage where the arrangement of colours has already been established and misguided interpretations have been preempted. µEDXRF surface analysis for Sr, Hg, Pb and Cu in the wall paintings of Cástulo proved particularly efficient for proper understanding of the procedure described. It has also allowed the detection of pigment mixtures in the search for proper tonalities namely, Egyptian blue and Green earths, calcite and Egyptian blue and cinnabar and lead.

The method described can be used not just for research on Roman heritage. Some of the minerals detected in the pigments discussed here had already been found in the decoration of caves, walls, floors and ceramic before Roman times^59,67,68^, namely hematite, goethite, cinnabar, calcite, and Egyptian blue. Others that are not discussed here can be added too, e.g. manganese oxides, Naples yellow, lapis lazuli, arsenic-based pigments (realgar, orpiment), gypsum… used in both pre- and post-Roman times^59^.

Finally, it should be noted that these results highlight the unique archaeological, historic and artistic value of the paintings of Cástulo and underline their relevance in their chronological frame, in the Iberian Peninsula and in the western Mediterranean basin.

## Methods

### Sample description

Eleven fragments of wall decorations of Room 6 were sampled for MRS and µEDXRF analysis (Fig. 2, Supplementary Figs S2–S12, Supplementary Table S14). The samples can be classified as follows:

- Fragments with figurative decoration. The following human figures were recorded: a Lar or household goddess (fragment 9), and an unidentified character carrying an object on its head (fragment 8). The following animal figures were recorded: a Little owl (*Athene noctua*), which is allegorical of goddess Minerva (fragment 4) and a goat, possibly the goat Amalthea who nursed infant Jupiter (fragment 11). Finally, fragment 1 depicted a quiver, one of the symbols of Cupid. Cupid is not identified in room 6, but he is depicted in the so-called ‘Los Amores’ Mosaic of neighbouring room 1. In these samples, red and yellow cover large areas or panels where the former overlaps the latter in part and both are delineated in white. The red panels background the figurative decoration with white, yellow, red, pink, brown, green and blue.
- Fragments with non-figurative decoration of surfaces and bands (fragments 2, 3, 5, 6, 7 and 10). The first group here consists of samples 5 and 7 (blue), with a single-coloured layer applied directly on to the wall. The remaining fragments (2, 3, 6 and 10) show overlying layers of paint, where yellow is partly covered by a layer of red and white lines separate yellow from red. The remaining colours were applied on these backgrounds.

Additional samples were needed for complete reconstruction of the scenes, but the data collected this far allowed to classify the paintings as of the third or fourth Pompeian style^69^^,^ according to the palette used and the mythological figures depicted.

### Micro Raman spectroscopy (MRS)

Three Raman spectrometers were used for the analysis of the samples: two laboratory equipments and one portable for samples bigger than the microscope measurement chamber available in the laboratory equipments. All the equipment and the experimental parameters are described in Tuñón et al.^68^ and Sánchez et al.^70^

The laboratory devices used were:

- A Renishaw ‘in via’ Reflex Spectrometer coupled with a confocal Leica DM LM microscope (CICT, University of Jaén), equipped with an argon ion laser (514.5 nm, 25 mW) and a diode laser (785 nm, 300 mW), and a Peltier-cooled CCD detector, calibrated to the 520.5 cm^−1^ line of silicon. The maximum laser output power was eventually reduced using neutral density filters. The spectra were acquired using the 50 × objective, in the 100 to 2000 cm^-1^ region, with spectral resolution of ca. 2 cm^−1^ (1,800 lines/mm grating) and ca. 1 cm^−1^ (1,200 lines/mm grating). Acquisition time was 10 s per accumulation, and the maximum number of accumulations was 10. A laser spot diameter of slightly more than 1 μm in diameter was achieved for both lasers (Supplementary Table S13).
- A Renishaw ‘in via’ Qontor Spectrometer coupled with a confocal Leica DM LM microscope (SCAI, University of Málaga), equipped with a solid state laser (473 nm, 25 mW), a Nd:YAG laser (532 nm, 50 mW) and a diode laser (785 nm, 300 mW), and a Peltier-cooled CCD detector, calibrated to the 520.5 cm^−1^ line of silicon. The maximum laser output power was eventually reduced using neutral density filters. The spectra were acquired using the 50 × objective, in the 100 to 2000 cm^−1^ region, with spectral resolution of ca. 1 cm^−1^ (2,400 lines/mm grating) and ca. 1 cm^−1^ (1,200 lines/mm grating). Acquisition time was set between 1 and 10 s per accumulation, and the maximum number of accumulations was 50. The laser spot diameter for each laser was 0.77 μm (473 nm), 0.86 μm (532 nm) and 1.27 μm (785 nm) (Supplementary Table S13).

The portable equipment used was a BWS445-785S innoRam™ Raman spectrometer (B&WTEK, Inc., Newark, USA) with a 785 nm excitation laser (maximum power of 300 mW) and a 4.5 cm^−1^ spectral resolution. The Raman microprobe can be mounted on a tripod with a motorized XYZ axis (MICROBEAM S.A, Barcelona, Spain) or on a microscope sampling stage (B&WTEK, Inc., Newark, USA). The measurements were carried out with a 50× objective. Experimental conditions were: exposure time ranged from 40 to 5,000 ms, maximum 100 acquisitions and a spectral range between 60 and 3,000 cm^−1^ (Supplementary Table S13).

### Micro energy dispersive X-ray fluorescence (µEDXRF)

This technique was applied according to the method described in Sánchez et al.^70^. An energy dispersive X-ray microfluorescence spectrometer (M4 Tornado, Bruker Nano) was used for this paper (CICT, University of Jaén). This spectrometer was equipped with a microfocus X-ray tube with and Rh anode, a polycapillary lens for X-ray focussing, and a 30-mm^2^ energy dispersive detector (SDD), calibrated to Kα_1_ line of Zr standard (15.775 keV). The sample chamber (200 × 160 × 120 mm) incorporated an XYZ motorized stage (330 mm × 170 mm) for sample positioning. A high resolution microscope (10×, 100×) was used to position the sample at the desired distance from the polycapillary. To increase the sensitivity of the low Z elements, the sample chamber can be brought under vacuum. For the analysis of the samples, a spot size of 25 μm was chosen at an operating X-ray tube voltage of 50 kV (30 W) and a current of 600 μA.

The concentration values were obtained with Bruker’s ESPRIT software (version 1.5) which included standardless quantification option (MQuant) based on a Fundamental Parameter method allowing to quantify a large variety of samples. Semiquantitative analysis was performed by calculation of a theoretical spectrum with the Sherman equation with correction of matrix effects. This calculated spectrum was compared with the measured one and then the quantification result was iteratively improved^71–73^. Reference samples (different from those commonly used for the standard calibration and maintenance of the instrument) were not used in this work due to the heterogeneity of the sample’s composition.

Two measurement methods were applied for µEDXRF analysis of wall paintings:

- Single-spot analysis. At least, three measurements of 120 s were carried out for each colour tonality. High resolution microscopy coupled to the µEDXRF equipment allowed further delineation of the areas so as to avoid sectors with discontinuous or fragmented decoration.
- Mapping analysis. Main mapping experimental parameters were adjusted for each mapping: step size (50 µm) and dwell time (2–5 ms/pixel). Map dimensions were changed according to the size of each analysed figure (Supplementary Table S15).

## Supplementary information

Supplementary information.

## Data Availability

The datasets generated during and/or analysed during the current study are available from the corresponding author on reasonable request.
