# Supplementary material for: The colours of Rome in the walls of Cástulo (Linares, Spain)
Source: Sci Rep. 2020 Jul 29;10:12739. doi: 10.1038/s41598-020-69334-y (PMC7391688; doi:10.1038/s41598-020-69334-y)
Supplement: Supplementary file 1 — Supplementary information. [file 41598_2020_69334_MOESM1_ESM.docx]

**Supplementary Information**

The colours of Rome in the walls of Cástulo (Linares, Spain).

José Tuñón1+, Alberto Sánchez1+, *, David J. Parras1+, Pilar Amate1+, Manuel Montejo2+, Bautista Ceprián1+

1University of Jaén, University Research Institute for Iberian Archaeology, Campus Las Lagunillas s/n, Edif. C6, Jaén, 23071, Spain

2University of Jaén, Department of Physical and Analytical Chemistry, Campus Las Lagunillas s/n, Edif. B3, Jaén, 23071, Spain

*Corresponding author: [vizcaino@ujaen.es](mailto:vizcaino@ujaen.es)

**Supplementary Tables**

Supplementary Table S1. Raman bands identified on the 11 wall painting fragments. Blue nod.: blue nodule.

| **Colour** | **Fragment** | **Raman bands (cm^-1^)** | **Compound** |
| --- | --- | --- | --- |
| Yellow | 1 | 155, 206, 246, 282, 300, 387, 419, 484, 553, 713*, 1086* | Goethite ^[1, 2]^, calcite* ^[3, 4]^ |
|  | 2 | 205, 279, 298, 395, 475, 551, 711*, 1089* |  |
|  | 4 | 151, 203, 240, 277, 296, 393, 415, 477, 546, 712*, 1085* |  |
|  | 6 | 209, 278, 298, 397, 478, 558, 713*, 1088* |  |
|  | 8 | 279, 298, 395, 463, 544 |  |
|  | 9 | 151, 279*, 297, 394, 554, 711*, 1086* |  |
|  | 10 | 150, 204, 241, 278*, 297, 394, 413, 477, 546, 680, 711*, 995, 1086* |  |
|  | 11 | 154, 282, 300, 387, 419, 551, 713*, 911, 1087* |  |
| Red | 1 | 225, 245, 294, 411, 498, 613, 902 | Hematite ^[2, 5]^, calcite* ^[3, 4]^ |
|  | 2 | 292, 409, 609 |  |
|  | 3 | 221, 241, 290, 406, 494, 608, 1088* |  |
|  | 4 | 224, 243, 292, 409, 497, 610 |  |
|  | 6 | 223, 245, 291, 410, 491, 611, 712*, 1089* |  |
|  | 8 | 222, 242, 289, 406, 496, 607 |  |
|  | 9 | 225, 245, 292, 410, 494, 611, 1086* |  |
|  | 10 | 222, 243, 290, 408, 610 |  |
|  | 11 | 225, 244, 293, 410, 495, 611, 747, 910 |  |
| Brown | 4 | 221, 288, 403, 492, 610, 668, 1330*, 1600* | Hematite ^[2, 5]^,  amorphous carbon* ^[4, 6, 7]^ |
|  | 9 | 148, 218, 283, 397, 491, 597, 1322*, 1594* |  |
| Pink | 1 | 143, 254, 286, 344 | Cinnabar ^[8, 9]^ |
|  | 9 | 152, 251, 283, 341 |  |
|  | 10 | 252, 285, 342 |  |
| Blue | 1 | 133, 159, 187, 226, 274, 379, 431, 467, 565, 786, 985, 1082 | Egyptian blue ^[10, 11]^ |
|  | 2 | 231, 430, 470, 573, 990, 1086 |  |
|  | 3 | 135, 162, 187, 227, 377, 430, 471, 566, 761, 787, 981, 1007, 1081 |  |
|  | 5 | 134, 161, 187, 227, 334, 377, 402, 430, 469, 566, 761, 786, 980, 1007, 1080, 1134 |  |
|  | 6 | 430, 571, 992, 1085 |  |
|  | 7 | 139, 165, 197, 232, 376, 430, 465, 570, 790, 989, 1015, 1086, 1104 |  |
| Green | 1 | 179, 215, 271, 320, 461, 553, 702, 1086 | Green earth (celadonite) ^[12, 13, 14, 15]^ |
|  | 2 | No Raman data | - |
|  | 3 | No Raman data | - |
|  | 4 | 177, 217, 272, 320, 459, 551, 588, 702, 966 | Green earth (celadonite) ^[12, 13, 14, 15]^ |
|  | 6 | No Raman data | - |
|  | 8 | No Raman data | - |
|  | 8 (blue nod.) | 376, 429, 475, 570, 989, 1011, 1085 | Egyptian blue ^[10, 11]^ |
|  | 9 | 270, 317, 702 | Green earth (glauconite/celadonite) ^[12, 13, 14, 15]^ |
|  | 9 (blue nod.) | 135, 195, 358, 376, 429, 473, 1011, 1084 | Egyptian blue ^[10, 11]^ |
|  | 10 | No Raman data | - |
|  | 10 (blue nod.) | 137, 165, 195, 230, 274, 377, 404, 430, 475, 570, 764, 787, 966, 987, 1014, 1085, 1144 | Egyptian blue ^[10, 11]^ |
| White | 1 | 152, 204, 280, 1085 | Calcite ^[3, 4]^ |
|  | 2 | 1085 |  |
|  | 3 | 1085 |  |
|  | 4 | 154, 206, 282, 1087 |  |
|  | 6 | 1085 |  |
|  | 8 | 1087 |  |
|  | 9 | 152, 205, 283, 705, 1085 |  |
|  | 10 | 1085 |  |
|  | 11 | 1085 |  |

Supplementary Table S2. μEDXRF single-spot analyses on fragment 1.

| **Fragment 1** | **Na2O** | **MgO** | **Al2O3** | **SiO2** | **P2O5** | **SO3** | **-Cl** | **K2O** | **CaO** | **TiO2** | **V2O5** | **Cr2O3** | **MnO** | **Fe2O3** | **CoO** | **CuO** | **ZnO** | **As2O3** | **Rb2O** | **SrO** | **Y2O3** | **ZrO2** | **BaO** | **HgO** | **PbO2** |
| --- | --- | --- | --- | --- | --- | --- | --- | --- | --- | --- | --- | --- | --- | --- | --- | --- | --- | --- | --- | --- | --- | --- | --- | --- | --- |
| Point 1 | 0,00 | 2,75 | 3,62 | 24,77 | 0,20 | 0,00 | 0,05 | 4,62 | 44,34 | 0,34 | 0,00 | 0,01 | 0,13 | 18,74 | 0,06 | 0,02 | 0,01 | 0,02 | 0,01 | 0,13 | 0,00 | 0,00 | 0,07 | 0,00 | 0,07 |
| Point 2 | 0,00 | 1,38 | 2,27 | 7,07 | 0,80 | 0,47 | 0,03 | 0,37 | 37,55 | 0,11 | 0,00 | 0,02 | 0,18 | 49,18 | 0,14 | 0,05 | 0,01 | 0,01 | 0,00 | 0,21 | 0,00 | 0,00 | 0,02 | 0,02 | 0,12 |
| Point 3 | 0,00 | 2,84 | 5,02 | 26,06 | 0,29 | 0,03 | 0,04 | 3,99 | 36,16 | 0,14 | 0,00 | 0,01 | 0,05 | 24,91 | 0,08 | 0,07 | 0,01 | 0,01 | 0,00 | 0,14 | 0,00 | 0,00 | 0,08 | 0,01 | 0,06 |
| Point 4 | 1,28 | 2,19 | 5,75 | 36,09 | 0,23 | 0,09 | 0,09 | 3,72 | 30,65 | 0,25 | 0,00 | 0,01 | 0,05 | 12,01 | 0,05 | 7,17 | 0,02 | 0,02 | 0,00 | 0,12 | 0,00 | 0,00 | 0,17 | 0,00 | 0,04 |
| Point 5 | 0,00 | 1,26 | 2,59 | 8,73 | 0,55 | 0,78 | 0,00 | 0,72 | 61,47 | 0,08 | 0,00 | 0,01 | 0,03 | 23,02 | 0,09 | 0,03 | 0,01 | 0,06 | 0,00 | 0,25 | 0,00 | 0,00 | 0,02 | 0,00 | 0,30 |
| Point 6 | 0,00 | 1,46 | 3,48 | 11,57 | 0,54 | 0,76 | 0,00 | 0,85 | 54,05 | 0,11 | 0,00 | 0,01 | 0,04 | 25,93 | 0,11 | 0,03 | 0,01 | 0,08 | 0,00 | 0,28 | 0,00 | 0,00 | 0,01 | 0,17 | 0,50 |
| Point 7 | 0,00 | 1,70 | 4,75 | 15,61 | 0,13 | 8,69 | 0,00 | 0,83 | 28,01 | 0,16 | 0,00 | 0,02 | 0,01 | 4,49 | 0,03 | 0,08 | 0,04 | 0,11 | 0,00 | 0,10 | 0,03 | 0,02 | 0,03 | 34,42 | 0,73 |
| Point 8 | 0,00 | 1,86 | 6,43 | 20,41 | 0,00 | 13,01 | 0,00 | 0,96 | 25,17 | 0,17 | 0,04 | 0,01 | 0,04 | 6,09 | 0,04 | 0,02 | 0,06 | 0,07 | 0,00 | 0,15 | 0,03 | 0,00 | 0,09 | 24,37 | 0,98 |
| Point 9 | 0,00 | 1,57 | 3,75 | 21,58 | 1,03 | 0,35 | 0,00 | 1,41 | 48,46 | 0,15 | 0,00 | 0,01 | 0,02 | 2,11 | 0,01 | 1,47 | 0,02 | 0,13 | 0,00 | 0,22 | 0,02 | 0,01 | 0,06 | 16,76 | 0,88 |
| Point 10 | 0,24 | 1,31 | 3,77 | 23,49 | 1,53 | 0,14 | 0,05 | 1,88 | 44,05 | 0,18 | 0,00 | 0,01 | 0,02 | 2,17 | 0,01 | 1,91 | 0,02 | 0,15 | 0,00 | 0,37 | 0,02 | 0,01 | 0,02 | 16,86 | 1,80 |
| Point 11 | 0,00 | 1,62 | 4,72 | 13,30 | 0,20 | 8,24 | 0,00 | 0,45 | 37,21 | 0,08 | 0,01 | 0,02 | 0,02 | 27,41 | 0,11 | 0,05 | 0,04 | 0,05 | 0,02 | 0,12 | 0,00 | 0,00 | 0,02 | 5,26 | 1,01 |
| Point 12 | 0,00 | 1,57 | 4,27 | 12,28 | 0,11 | 9,72 | 0,00 | 0,69 | 31,51 | 0,16 | 0,03 | 0,02 | 0,03 | 6,81 | 0,03 | 0,04 | 0,07 | 0,12 | 0,01 | 0,12 | 0,03 | 0,00 | 0,05 | 31,64 | 0,71 |
| Point 13 | 0,00 | 1,87 | 4,09 | 11,46 | 0,00 | 15,94 | 0,00 | 0,56 | 27,62 | 0,10 | 0,04 | 0,03 | 0,03 | 9,94 | 0,07 | 0,03 | 0,08 | 0,02 | 0,00 | 0,24 | 0,03 | 0,00 | 0,08 | 26,72 | 1,07 |
| Point 14 | 0,00 | 3,23 | 4,93 | 21,94 | 0,35 | 0,22 | 0,00 | 4,82 | 41,55 | 0,19 | 0,01 | 0,01 | 1,04 | 12,93 | 0,05 | 0,09 | 0,02 | 0,18 | 0,00 | 0,14 | 0,01 | 0,00 | 0,11 | 7,17 | 1,02 |
| Point 15 | 0,00 | 1,35 | 3,09 | 9,54 | 0,65 | 1,37 | 0,00 | 1,29 | 64,18 | 0,09 | 0,00 | 0,00 | 0,03 | 5,65 | 0,04 | 0,01 | 0,02 | 0,09 | 0,00 | 0,41 | 0,01 | 0,01 | 0,03 | 11,10 | 1,02 |
| Point 16 | 0,00 | 1,88 | 3,50 | 10,67 | 0,35 | 1,93 | 0,00 | 0,43 | 60,41 | 0,41 | 0,00 | 0,01 | 0,22 | 14,19 | 0,07 | 0,05 | 0,02 | 0,07 | 0,00 | 0,51 | 0,00 | 0,00 | 0,00 | 2,73 | 2,54 |
| Point 17 | 0,00 | 1,56 | 3,33 | 10,25 | 0,27 | 3,57 | 0,00 | 0,48 | 58,47 | 0,61 | 0,00 | 0,01 | 0,04 | 10,24 | 0,06 | 0,06 | 0,04 | 0,05 | 0,01 | 0,53 | 0,02 | 0,00 | 0,01 | 8,37 | 2,04 |
| Point 18 | 0,00 | 1,31 | 3,35 | 9,77 | 0,33 | 3,66 | 0,00 | 0,41 | 57,77 | 0,19 | 0,00 | 0,01 | 0,03 | 16,17 | 0,09 | 0,02 | 0,03 | 0,12 | 0,04 | 0,54 | 0,01 | 0,00 | 0,01 | 4,37 | 1,78 |
| Point 19 | 0,00 | 2,58 | 4,42 | 25,41 | 0,14 | 0,00 | 0,01 | 4,46 | 44,75 | 0,15 | 0,00 | 0,01 | 0,03 | 10,04 | 0,04 | 0,09 | 0,02 | 0,05 | 0,00 | 0,34 | 0,01 | 0,03 | 0,05 | 5,53 | 1,80 |
| Point 20 | 0,00 | 1,49 | 3,95 | 28,65 | 0,21 | 0,41 | 0,00 | 0,96 | 49,51 | 0,11 | 0,00 | 0,02 | 0,06 | 5,84 | 0,03 | 3,41 | 0,01 | 0,11 | 0,00 | 0,41 | 0,00 | 0,00 | 0,01 | 2,77 | 2,01 |
| Point 21 | 0,00 | 1,56 | 4,15 | 23,97 | 1,16 | 0,17 | 0,01 | 1,24 | 52,87 | 0,12 | 0,00 | 0,00 | 0,03 | 3,93 | 0,04 | 2,98 | 0,02 | 0,19 | 0,00 | 0,52 | 0,01 | 0,00 | 0,03 | 5,04 | 1,98 |
| Point 22 | 0,56 | 1,57 | 4,35 | 26,48 | 0,86 | 0,23 | 0,04 | 1,23 | 38,75 | 0,12 | 0,00 | 0,03 | 0,02 | 3,61 | 0,02 | 6,64 | 0,01 | 0,14 | 0,00 | 0,30 | 0,02 | 0,02 | 0,01 | 13,60 | 1,40 |
| Point 23 | 0,00 | 1,52 | 3,29 | 10,05 | 0,47 | 2,37 | 0,00 | 0,40 | 70,35 | 0,10 | 0,00 | 0,01 | 0,10 | 4,74 | 0,02 | 0,03 | 0,02 | 0,16 | 0,01 | 0,63 | 0,00 | 0,00 | 0,03 | 2,47 | 3,22 |
| Point 24 | 0,00 | 1,29 | 3,25 | 8,56 | 0,00 | 10,83 | 0,00 | 0,40 | 41,58 | 0,07 | 0,01 | 0,02 | 0,04 | 19,67 | 0,11 | 0,07 | 0,05 | 0,00 | 0,00 | 0,40 | 0,03 | 0,00 | 0,04 | 10,89 | 2,68 |
| Point 25 | 0,00 | 1,25 | 4,35 | 13,25 | 0,24 | 8,95 | 0,00 | 0,60 | 37,68 | 0,83 | 0,02 | 0,03 | 0,04 | 17,81 | 0,05 | 0,03 | 0,07 | 0,21 | 0,00 | 0,37 | 0,02 | 0,01 | 0,06 | 12,32 | 1,81 |
| Point 26 | 0,00 | 1,44 | 2,75 | 8,41 | 0,48 | 2,22 | 0,00 | 0,33 | 63,88 | 0,10 | 0,00 | 0,01 | 0,10 | 16,29 | 0,08 | 0,03 | 0,02 | 0,07 | 0,00 | 0,26 | 0,00 | 0,00 | 0,00 | 2,13 | 1,39 |
| Point 27 | 0,00 | 1,07 | 1,94 | 7,97 | 0,67 | 1,20 | 0,00 | 0,37 | 80,70 | 0,09 | 0,00 | 0,00 | 0,04 | 3,83 | 0,02 | 0,02 | 0,01 | 0,14 | 0,00 | 0,81 | 0,00 | 0,00 | 0,02 | 0,08 | 1,02 |
| Point 28 | 0,14 | 1,27 | 1,41 | 4,57 | 0,40 | 0,57 | 0,04 | 0,30 | 52,28 | 0,11 | 0,00 | 0,02 | 0,05 | 38,41 | 0,09 | 0,04 | 0,02 | 0,01 | 0,00 | 0,19 | 0,00 | 0,00 | 0,02 | 0,01 | 0,08 |
| Point 29 | 0,00 | 0,98 | 2,33 | 7,73 | 0,81 | 1,81 | 0,00 | 0,45 | 72,09 | 0,08 | 0,00 | 0,00 | 0,01 | 7,29 | 0,06 | 0,01 | 0,02 | 0,17 | 0,00 | 1,08 | 0,00 | 0,00 | 0,02 | 3,34 | 1,71 |
| Point 30 | 0,00 | 1,35 | 4,97 | 13,64 | 0,12 | 8,14 | 0,00 | 0,73 | 36,82 | 0,16 | 0,00 | 0,03 | 0,02 | 18,98 | 0,08 | 0,06 | 0,07 | 0,13 | 0,00 | 0,44 | 0,03 | 0,00 | 0,04 | 12,15 | 2,04 |
| Point 31 | 0,00 | 1,21 | 1,56 | 4,55 | 0,56 | 0,89 | 0,00 | 0,28 | 72,67 | 0,04 | 0,00 | 0,00 | 0,02 | 2,37 | 0,02 | 0,06 | 0,02 | 0,16 | 0,00 | 0,73 | 0,02 | 0,00 | 0,02 | 11,45 | 3,35 |
| Point 32 | 0,00 | 1,91 | 1,43 | 8,86 | 0,62 | 0,75 | 0,00 | 0,38 | 53,37 | 0,92 | 0,00 | 0,01 | 0,04 | 30,22 | 0,08 | 0,11 | 0,01 | 0,06 | 0,13 | 0,18 | 0,00 | 0,46 | 0,03 | 0,00 | 0,42 |
| Point 33 | 0,00 | 1,02 | 1,47 | 9,73 | 0,55 | 1,08 | 0,00 | 0,57 | 66,08 | 0,07 | 0,00 | 0,00 | 0,11 | 17,60 | 0,09 | 0,02 | 0,01 | 0,10 | 0,00 | 0,69 | 0,00 | 0,00 | 0,00 | 0,00 | 0,82 |
| Point 34 | 0,00 | 1,10 | 1,59 | 5,37 | 0,52 | 0,85 | 0,00 | 0,30 | 37,73 | 0,12 | 0,00 | 0,02 | 0,05 | 51,76 | 0,15 | 0,09 | 0,01 | 0,03 | 0,02 | 0,16 | 0,00 | 0,00 | 0,01 | 0,00 | 0,12 |
| Point 35 | 0,00 | 1,12 | 3,00 | 10,92 | 0,90 | 0,76 | 0,00 | 0,51 | 60,96 | 0,11 | 0,00 | 0,00 | 0,08 | 19,47 | 0,07 | 0,28 | 0,01 | 0,15 | 0,00 | 0,36 | 0,00 | 0,00 | 0,02 | 0,00 | 1,28 |
| Point 36 | 0,07 | 1,01 | 1,31 | 6,63 | 0,63 | 1,28 | 0,00 | 0,50 | 74,86 | 0,14 | 0,00 | 0,00 | 0,09 | 12,38 | 0,05 | 0,02 | 0,01 | 0,05 | 0,00 | 0,65 | 0,00 | 0,00 | 0,02 | 0,00 | 0,29 |
| Point 37 | 0,04 | 1,80 | 1,23 | 4,82 | 0,60 | 0,65 | 0,01 | 0,31 | 50,25 | 0,11 | 0,00 | 0,02 | 0,11 | 39,55 | 0,10 | 0,04 | 0,05 | 0,02 | 0,00 | 0,16 | 0,00 | 0,00 | 0,01 | 0,01 | 0,12 |

Supplementary Table S3. μEDXRF single-spot analyses on fragment 2.

| **Fragment 2** | **Na2O** | **MgO** | **Al2O3** | **SiO2** | **P2O5** | **SO3** | **-Cl** | **K2O** | **CaO** | **TiO2** | **V2O5** | **Cr2O3** | **MnO** | **Fe2O3** | **CoO** | **CuO** | **ZnO** | **Rb2O** | **SrO** | **BaO** | **PbO2** |
| --- | --- | --- | --- | --- | --- | --- | --- | --- | --- | --- | --- | --- | --- | --- | --- | --- | --- | --- | --- | --- | --- |
| Point 1 | 0,00 | 0,87 | 6,10 | 15,09 | 0,39 | 0,13 | 0,00 | 0,89 | 52,50 | 0,40 | 0,02 | 0,01 | 0,23 | 22,95 | 0,05 | 0,02 | 0,04 | 0,00 | 0,17 | 0,06 | 0,08 |
| Point 2 | 0,00 | 2,24 | 1,79 | 6,92 | 0,27 | 0,12 | 0,01 | 0,24 | 49,27 | 0,10 | 0,00 | 0,02 | 0,06 | 38,50 | 0,08 | 0,06 | 0,01 | 0,00 | 0,20 | 0,03 | 0,07 |
| Point 3 | 0,18 | 0,81 | 1,48 | 6,28 | 0,18 | 0,23 | 0,00 | 0,17 | 80,73 | 0,09 | 0,00 | 0,00 | 0,02 | 8,50 | 0,08 | 0,24 | 0,01 | 0,01 | 0,91 | 0,03 | 0,07 |
| Point 4 | 1,13 | 1,37 | 2,78 | 54,25 | 0,24 | 0,00 | 0,01 | 0,43 | 19,63 | 0,07 | 0,00 | 0,00 | 0,02 | 0,59 | 0,00 | 18,99 | 0,05 | 0,00 | 0,37 | 0,01 | 0,04 |
| Point 5 | 0,65 | 2,05 | 3,20 | 57,19 | 0,05 | 0,11 | 0,02 | 0,60 | 31,79 | 0,15 | 0,02 | 0,00 | 0,03 | 1,11 | 0,00 | 2,90 | 0,01 | 0,00 | 0,08 | 0,01 | 0,02 |
| Point 6 | 0,77 | 0,95 | 1,12 | 4,18 | 0,18 | 0,28 | 0,00 | 0,26 | 89,40 | 0,09 | 0,00 | 0,01 | 0,04 | 1,38 | 0,01 | 0,28 | 0,01 | 0,00 | 0,98 | 0,02 | 0,05 |
| Point 7 | 0,78 | 2,21 | 4,32 | 21,75 | 1,34 | 0,15 | 0,00 | 1,93 | 62,88 | 0,22 | 0,00 | 0,01 | 0,05 | 3,74 | 0,00 | 0,34 | 0,01 | 0,12 | 0,09 | 0,02 | 0,05 |
| Point 8 | 0,20 | 1,35 | 2,44 | 15,23 | 0,62 | 0,97 | 0,04 | 0,47 | 68,40 | 0,06 | 0,05 | 0,00 | 0,07 | 5,01 | 0,04 | 4,44 | 0,02 | 0,00 | 0,44 | 0,09 | 0,05 |
| Point 9 | 0,46 | 0,70 | 1,64 | 24,11 | 0,41 | 0,71 | 0,03 | 0,31 | 61,07 | 0,03 | 0,00 | 0,00 | 0,02 | 2,03 | 0,02 | 7,78 | 0,02 | 0,00 | 0,55 | 0,06 | 0,03 |
| Point 10 | 1,63 | 1,45 | 6,31 | 22,48 | 0,95 | 0,78 | 0,10 | 1,04 | 56,33 | 0,16 | 0,00 | 0,01 | 0,02 | 0,92 | 0,01 | 6,97 | 0,01 | 0,05 | 0,78 | 0,00 | 0,02 |

Supplementary Table S4. μEDXRF single-spot analyses on fragment 3.

| **Fragment 3** | **Na2O** | **MgO** | **Al2O3** | **SiO2** | **P2O5** | **SO3** | **-Cl** | **K2O** | **CaO** | **TiO2** | **V2O5** | **Cr2O3** | **MnO** | **Fe2O3** | **CoO** | **CuO** | **ZnO** | **Rb2O** | **SrO** | **BaO** | **PbO2** |
| --- | --- | --- | --- | --- | --- | --- | --- | --- | --- | --- | --- | --- | --- | --- | --- | --- | --- | --- | --- | --- | --- |
| Point 1 | 0,19 | 1,79 | 2,53 | 7,45 | 0,40 | 0,22 | 0,02 | 0,27 | 32,22 | 0,10 | 0,00 | 0,02 | 0,03 | 54,38 | 0,14 | 0,04 | 0,01 | 0,00 | 0,10 | 0,02 | 0,07 |
| Point 2 | 0,00 | 2,52 | 2,99 | 9,73 | 0,52 | 0,16 | 0,02 | 0,32 | 50,12 | 0,13 | 0,00 | 0,02 | 0,04 | 33,00 | 0,06 | 0,04 | 0,01 | 0,00 | 0,20 | 0,02 | 0,07 |
| Point 3 | 0,00 | 2,01 | 3,82 | 30,42 | 1,47 | 0,12 | 0,02 | 3,69 | 50,10 | 0,27 | 0,00 | 0,02 | 0,04 | 7,50 | 0,01 | 0,29 | 0,01 | 0,01 | 0,15 | 0,01 | 0,05 |
| Point 4 | 0,00 | 2,31 | 3,94 | 21,36 | 0,92 | 0,18 | 0,00 | 2,67 | 61,41 | 0,23 | 0,00 | 0,01 | 0,06 | 6,24 | 0,01 | 0,39 | 0,01 | 0,02 | 0,13 | 0,04 | 0,06 |
| Point 5 | 0,00 | 4,72 | 6,44 | 48,85 | 0,20 | 0,12 | 0,00 | 9,79 | 9,33 | 0,28 | 0,00 | 0,02 | 0,07 | 19,58 | 0,05 | 0,09 | 0,00 | 0,01 | 0,13 | 0,27 | 0,06 |
| Point 6 | 0,00 | 1,32 | 2,96 | 9,23 | 0,39 | 0,24 | 0,00 | 0,25 | 81,42 | 0,10 | 0,00 | 0,00 | 0,05 | 2,64 | 0,04 | 0,03 | 0,01 | 0,04 | 1,19 | 0,03 | 0,08 |
| Point 7 | 0,00 | 1,97 | 3,25 | 9,63 | 0,35 | 0,27 | 0,00 | 0,23 | 82,31 | 0,09 | 0,00 | 0,00 | 0,11 | 1,24 | 0,00 | 0,17 | 0,02 | 0,00 | 0,18 | 0,03 | 0,14 |
| Point 8 | 3,28 | 0,97 | 2,71 | 58,04 | 0,00 | 0,06 | 0,16 | 0,86 | 17,80 | 0,08 | 0,02 | 0,00 | 0,03 | 0,66 | 0,00 | 15,04 | 0,04 | 0,01 | 0,15 | 0,03 | 0,05 |
| Point 9 | 0,31 | 2,61 | 7,47 | 45,18 | 0,76 | 0,17 | 0,07 | 1,57 | 34,64 | 0,29 | 0,00 | 0,01 | 0,06 | 1,91 | 0,00 | 4,68 | 0,02 | 0,00 | 0,18 | 0,01 | 0,06 |
| Point 10 | 0,18 | 1,33 | 2,78 | 25,28 | 0,50 | 0,83 | 0,00 | 0,43 | 55,37 | 0,08 | 0,00 | 0,00 | 0,06 | 4,93 | 0,04 | 7,64 | 0,01 | 0,00 | 0,49 | 0,01 | 0,04 |
| Point 11 | 0,00 | 2,35 | 2,48 | 10,88 | 0,64 | 0,80 | 0,05 | 0,29 | 79,64 | 0,08 | 0,01 | 0,00 | 0,19 | 0,74 | 0,00 | 1,73 | 0,02 | 0,03 | 0,02 | 0,00 | 0,02 |

Supplementary Table S5. μEDXRF single-spot analyses on fragment 4.

| **Fragment 4** | **Na2O** | **MgO** | **Al2O3** | **SiO2** | **P2O5** | **SO3** | **K2O** | **CaO** | **TiO2** | **V2O5** | **Cr2O3** | **MnO** | **Fe2O3** | **CoO** | **CuO** | **ZnO** | **As2O3** | **Rb2O** | **ZrO2** | **BaO** | **SrO** | **PbO2** |
| --- | --- | --- | --- | --- | --- | --- | --- | --- | --- | --- | --- | --- | --- | --- | --- | --- | --- | --- | --- | --- | --- | --- |
| Point 1 | 0,00 | 1,01 | 6,02 | 17,84 | 2,51 | 0,17 | 1,09 | 43,11 | 0,35 | 0,01 | 0,00 | 0,27 | 27,12 | 0,07 | 0,02 | 0,08 | 0,03 | 0,00 | 0,00 | 0,04 | 0,20 | 0,05 |
| Point 2 | 0,88 | 1,05 | 2,07 | 6,79 | 2,84 | 0,27 | 0,37 | 78,36 | 0,13 | 0,00 | 0,00 | 0,10 | 5,79 | 0,04 | 0,13 | 0,02 | 0,00 | 0,03 | 0,04 | 0,03 | 0,76 | 0,28 |
| Point 3 | 0,19 | 0,64 | 1,51 | 7,84 | 3,22 | 0,28 | 0,45 | 54,41 | 0,23 | 0,01 | 0,01 | 0,12 | 30,51 | 0,08 | 0,05 | 0,04 | 0,03 | 0,00 | 0,00 | 0,03 | 0,27 | 0,10 |
| Point 4 | 0,46 | 1,69 | 2,13 | 10,97 | 2,40 | 0,26 | 0,76 | 52,00 | 0,11 | 0,00 | 0,01 | 0,07 | 28,59 | 0,09 | 0,07 | 0,02 | 0,02 | 0,00 | 0,00 | 0,04 | 0,23 | 0,09 |
| Point 5 | 0,12 | 1,35 | 3,07 | 18,11 | 2,51 | 0,18 | 2,85 | 45,74 | 0,17 | 0,00 | 0,01 | 0,07 | 25,17 | 0,11 | 0,08 | 0,01 | 0,02 | 0,00 | 0,00 | 0,04 | 0,31 | 0,07 |
| Point 6 | 0,23 | 2,41 | 3,31 | 26,22 | 2,23 | 0,19 | 5,66 | 33,70 | 0,15 | 0,01 | 0,01 | 0,14 | 24,86 | 0,09 | 0,48 | 0,02 | 0,02 | 0,00 | 0,00 | 0,04 | 0,16 | 0,07 |
| Point 7 | 0,12 | 1,68 | 3,62 | 21,19 | 2,92 | 0,18 | 3,84 | 46,05 | 0,22 | 0,00 | 0,01 | 0,08 | 19,34 | 0,08 | 0,03 | 0,02 | 0,02 | 0,00 | 0,00 | 0,31 | 0,21 | 0,08 |
| Point 8 | 0,00 | 1,88 | 1,82 | 11,07 | 2,12 | 0,22 | 0,86 | 60,96 | 0,12 | 0,01 | 0,00 | 0,20 | 20,22 | 0,05 | 0,04 | 0,04 | 0,02 | 0,00 | 0,00 | 0,05 | 0,23 | 0,09 |
| Point 9 | 0,33 | 1,80 | 2,01 | 7,26 | 2,62 | 0,22 | 0,45 | 49,84 | 0,16 | 0,00 | 0,02 | 0,07 | 34,69 | 0,07 | 0,04 | 0,04 | 0,03 | 0,11 | 0,00 | 0,02 | 0,14 | 0,08 |
| Point 10 | 0,25 | 1,32 | 3,93 | 13,57 | 2,27 | 0,20 | 0,69 | 51,55 | 0,27 | 0,01 | 0,01 | 0,34 | 25,04 | 0,07 | 0,03 | 0,06 | 0,03 | 0,00 | 0,00 | 0,02 | 0,27 | 0,09 |
| Point 11 | 0,53 | 0,54 | 1,35 | 3,94 | 1,60 | 0,40 | 0,24 | 86,97 | 0,12 | 0,00 | 0,01 | 0,06 | 2,75 | 0,03 | 0,09 | 0,02 | 0,01 | 0,00 | 0,00 | 0,01 | 1,26 | 0,06 |
| Point 12 | 0,05 | 1,00 | 5,25 | 17,21 | 1,82 | 0,56 | 0,89 | 42,38 | 0,39 | 0,02 | 0,01 | 0,44 | 29,40 | 0,08 | 0,02 | 0,05 | 0,03 | 0,00 | 0,00 | 0,05 | 0,34 | 0,04 |
| Point 13 | 0,18 | 0,87 | 1,68 | 7,51 | 2,68 | 0,38 | 0,54 | 78,70 | 0,09 | 0,00 | 0,00 | 0,09 | 6,29 | 0,05 | 0,02 | 0,03 | 0,03 | 0,00 | 0,00 | 0,03 | 0,77 | 0,07 |
| Point 14 | 0,05 | 0,67 | 4,60 | 17,17 | 2,01 | 0,17 | 0,99 | 37,45 | 0,40 | 0,03 | 0,01 | 0,54 | 35,37 | 0,11 | 0,02 | 0,06 | 0,03 | 0,01 | 0,00 | 0,05 | 0,20 | 0,06 |
| Point 15 | 0,50 | 2,61 | 4,52 | 28,19 | 2,07 | 0,14 | 4,38 | 41,08 | 0,34 | 0,02 | 0,02 | 0,09 | 15,63 | 0,06 | 0,02 | 0,02 | 0,02 | 0,02 | 0,00 | 0,05 | 0,15 | 0,05 |
| Point 16 | 0,15 | 2,67 | 4,13 | 37,77 | 1,53 | 0,16 | 6,43 | 28,07 | 0,13 | 0,01 | 0,01 | 0,06 | 18,57 | 0,06 | 0,01 | 0,01 | 0,02 | 0,03 | 0,00 | 0,02 | 0,12 | 0,04 |
| Point 17 | 0,66 | 0,96 | 2,71 | 25,15 | 2,04 | 0,22 | 3,18 | 43,59 | 0,24 | 0,00 | 0,01 | 0,10 | 16,68 | 0,08 | 4,05 | 0,02 | 0,01 | 0,00 | 0,00 | 0,04 | 0,19 | 0,09 |
| Point 18 | 1,28 | 1,46 | 4,28 | 15,60 | 4,34 | 0,29 | 2,04 | 51,86 | 0,30 | 0,00 | 0,00 | 3,10 | 14,68 | 0,07 | 0,11 | 0,03 | 0,04 | 0,00 | 0,00 | 0,16 | 0,20 | 0,15 |
| Point 19 | 0,19 | 0,80 | 3,88 | 56,61 | 2,04 | 0,18 | 1,34 | 21,59 | 0,27 | 0,00 | 0,01 | 0,03 | 12,76 | 0,05 | 0,04 | 0,01 | 0,01 | 0,00 | 0,00 | 0,01 | 0,17 | 0,04 |
| Point 20 | 0,07 | 2,19 | 1,82 | 8,14 | 2,31 | 0,28 | 0,26 | 63,08 | 0,14 | 0,00 | 0,01 | 0,10 | 21,13 | 0,03 | 0,03 | 0,01 | 0,02 | 0,04 | 0,04 | 0,05 | 0,18 | 0,07 |
| Point 21 | 0,00 | 1,28 | 5,06 | 65,65 | 1,34 | 0,21 | 1,95 | 19,20 | 0,38 | 0,00 | 0,01 | 0,06 | 4,66 | 0,02 | 0,02 | 0,02 | 0,01 | 0,01 | 0,00 | 0,03 | 0,08 | 0,03 |
| Point 22 | 0,43 | 0,80 | 5,69 | 21,52 | 2,52 | 0,58 | 3,62 | 46,93 | 0,42 | 0,04 | 0,02 | 0,10 | 16,78 | 0,05 | 0,03 | 0,03 | 0,03 | 0,05 | 0,06 | 0,10 | 0,12 | 0,09 |
| Point 23 | 0,00 | 0,81 | 1,97 | 6,43 | 0,42 | 0,31 | 0,20 | 72,68 | 0,08 | 0,00 | 0,00 | 0,06 | 16,00 | 0,07 | 0,02 | 0,02 | 0,07 | 0,02 | 0,00 | 0,03 | 0,43 | 0,36 |
| Point 24 | 0,10 | 1,61 | 1,79 | 6,45 | 0,49 | 0,32 | 0,40 | 45,78 | 0,23 | 0,00 | 0,02 | 0,10 | 42,01 | 0,09 | 0,29 | 0,03 | 0,03 | 0,02 | 0,00 | 0,03 | 0,11 | 0,11 |
| Point 25 | 0,14 | 1,06 | 3,42 | 14,02 | 2,31 | 0,40 | 0,57 | 56,32 | 0,25 | 0,00 | 0,01 | 0,10 | 19,91 | 0,06 | 0,06 | 0,03 | 0,08 | 0,00 | 0,00 | 0,03 | 0,85 | 0,38 |
| Point 26 | 0,03 | 0,61 | 2,67 | 17,55 | 2,12 | 0,43 | 0,82 | 57,43 | 0,24 | 0,00 | 0,00 | 0,09 | 15,98 | 0,05 | 0,09 | 0,03 | 0,09 | 0,00 | 0,00 | 0,03 | 1,28 | 0,47 |
| Point 27 | 0,40 | 0,36 | 1,59 | 6,28 | 2,45 | 0,28 | 0,32 | 80,25 | 0,12 | 0,00 | 0,00 | 0,10 | 5,24 | 0,02 | 0,04 | 0,02 | 0,07 | 0,00 | 0,00 | 0,01 | 2,01 | 0,42 |
| Point 28 | 0,45 | 0,57 | 2,18 | 6,93 | 2,53 | 0,27 | 0,30 | 81,04 | 0,20 | 0,00 | 0,01 | 0,08 | 3,45 | 0,03 | 0,04 | 0,01 | 0,05 | 0,00 | 0,00 | 0,03 | 1,49 | 0,33 |
| Point 29 | 0,44 | 0,96 | 2,45 | 14,85 | 2,38 | 0,27 | 0,51 | 60,84 | 0,26 | 0,00 | 0,00 | 0,09 | 15,22 | 0,05 | 0,48 | 0,02 | 0,07 | 0,00 | 0,00 | 0,02 | 0,81 | 0,29 |
| Point 30 | 0,36 | 0,50 | 1,96 | 9,31 | 2,35 | 0,21 | 0,58 | 33,18 | 0,27 | 0,02 | 0,02 | 0,08 | 49,46 | 0,10 | 0,07 | 0,09 | 0,14 | 0,00 | 0,00 | 0,06 | 0,74 | 0,51 |
| Point 31 | 0,49 | 0,91 | 3,63 | 13,15 | 2,71 | 0,25 | 1,01 | 67,84 | 0,22 | 0,00 | 0,00 | 0,08 | 8,64 | 0,04 | 0,03 | 0,01 | 0,06 | 0,00 | 0,00 | 0,01 | 0,54 | 0,36 |
| Point 32 | 0,97 | 0,66 | 1,79 | 7,91 | 2,59 | 0,29 | 0,36 | 78,90 | 0,11 | 0,00 | 0,00 | 0,06 | 4,82 | 0,04 | 0,03 | 0,01 | 0,05 | 0,01 | 0,00 | 0,03 | 0,99 | 0,36 |
| Point 33 | 0,06 | 0,86 | 1,61 | 9,53 | 2,73 | 0,37 | 0,39 | 69,59 | 0,15 | 0,00 | 0,01 | 0,12 | 11,76 | 0,03 | 0,03 | 0,02 | 0,11 | 0,00 | 0,00 | 0,03 | 1,60 | 0,99 |
| Point 34 | 0,22 | 1,96 | 3,40 | 12,69 | 3,04 | 0,28 | 0,51 | 56,86 | 0,19 | 0,00 | 0,01 | 0,40 | 18,72 | 0,07 | 0,07 | 0,03 | 0,08 | 0,00 | 0,00 | 0,03 | 0,99 | 0,47 |
| Point 35 | 1,39 | 1,31 | 1,93 | 9,92 | 3,90 | 0,47 | 0,32 | 61,32 | 0,30 | 0,00 | 0,00 | 0,14 | 17,76 | 0,05 | 0,11 | 0,02 | 0,07 | 0,00 | 0,00 | 0,03 | 0,75 | 0,22 |
| Point 36 | 0,00 | 1,29 | 1,68 | 9,33 | 2,63 | 0,22 | 0,36 | 54,15 | 0,22 | 0,00 | 0,01 | 0,25 | 27,83 | 0,06 | 0,07 | 0,03 | 0,09 | 0,00 | 0,00 | 0,01 | 1,30 | 0,48 |
| Point 37 | 0,77 | 0,78 | 1,57 | 5,42 | 3,68 | 0,35 | 0,24 | 79,00 | 0,08 | 0,00 | 0,00 | 0,05 | 4,97 | 0,04 | 0,04 | 0,02 | 0,12 | 0,00 | 0,00 | 0,03 | 1,55 | 1,28 |
| Point 38 | 0,30 | 1,10 | 2,63 | 10,56 | 3,75 | 0,61 | 0,66 | 63,77 | 0,34 | 0,00 | 0,00 | 0,20 | 14,63 | 0,04 | 0,08 | 0,02 | 0,05 | 0,00 | 0,07 | 0,02 | 0,77 | 0,40 |
| Point 39 | 0,40 | 1,30 | 2,94 | 10,89 | 2,42 | 0,34 | 0,32 | 72,96 | 0,11 | 0,00 | 0,00 | 0,06 | 6,65 | 0,04 | 0,03 | 0,01 | 0,07 | 0,01 | 0,00 | 0,02 | 1,02 | 0,39 |
| Point 40 | 0,00 | 1,89 | 3,09 | 12,46 | 1,85 | 0,29 | 0,48 | 57,70 | 0,19 | 0,00 | 0,00 | 0,12 | 19,99 | 0,05 | 0,10 | 0,03 | 0,09 | 0,00 | 0,00 | 0,04 | 1,26 | 0,37 |
| Point 41 | 0,52 | 1,09 | 3,44 | 11,91 | 2,94 | 0,26 | 0,56 | 68,93 | 0,17 | 0,00 | 0,00 | 0,03 | 6,85 | 0,03 | 0,02 | 0,01 | 0,10 | 0,00 | 0,00 | 0,02 | 1,84 | 1,27 |
| Point 42 | 0,34 | 1,42 | 3,05 | 13,94 | 3,22 | 0,35 | 0,65 | 52,99 | 0,25 | 0,00 | 0,02 | 0,38 | 21,53 | 0,08 | 0,12 | 0,03 | 0,10 | 0,00 | 0,00 | 0,05 | 0,96 | 0,50 |
| Point 43 | 0,32 | 0,69 | 2,17 | 24,48 | 3,15 | 0,29 | 0,50 | 45,82 | 0,27 | 0,00 | 0,00 | 0,11 | 20,23 | 0,06 | 0,34 | 0,04 | 0,07 | 0,00 | 0,00 | 0,02 | 1,07 | 0,39 |
| Point 44 | 0,43 | 3,04 | 4,30 | 36,69 | 2,62 | 0,26 | 9,44 | 20,69 | 0,21 | 0,01 | 0,02 | 0,13 | 21,10 | 0,06 | 0,03 | 0,01 | 0,01 | 0,00 | 0,00 | 0,08 | 0,64 | 0,24 |
| Point 45 | 0,74 | 0,67 | 1,28 | 4,61 | 2,37 | 0,20 | 0,12 | 83,32 | 0,07 | 0,00 | 0,00 | 0,03 | 1,47 | 0,02 | 0,03 | 0,01 | 0,15 | 0,00 | 0,00 | 0,02 | 2,53 | 2,36 |
| Point 46 | 0,64 | 0,69 | 1,47 | 20,40 | 3,25 | 0,86 | 0,41 | 60,70 | 0,06 | 0,00 | 0,00 | 0,05 | 3,86 | 0,03 | 6,78 | 0,02 | 0,02 | 0,00 | 0,00 | 0,04 | 0,62 | 0,04 |
| Point 47 | 0,48 | 0,64 | 1,23 | 25,13 | 2,90 | 0,88 | 0,42 | 49,85 | 0,20 | 0,00 | 0,00 | 0,03 | 1,92 | 0,03 | 15,55 | 0,03 | 0,03 | 0,00 | 0,00 | 0,00 | 0,54 | 0,05 |
| Point 48 | 1,07 | 1,39 | 1,51 | 22,44 | 3,35 | 0,77 | 0,39 | 56,81 | 0,06 | 0,00 | 0,04 | 0,02 | 1,77 | 0,01 | 9,45 | 0,02 | 0,00 | 0,01 | 0,00 | 0,00 | 0,58 | 0,28 |

Supplementary Table S6. μEDXRF single-spot analyses on fragment 5.

| **Fragment 5** | **Na2O** | **MgO** | **Al2O3** | **SiO2** | **P2O5** | **SO3** | **K2O** | **CaO** | **TiO2** | **V2O5** | **MnO** | **Fe2O3** | **CoO** | **CuO** | **ZnO** | **As2O3** | **Rb2O** | **SrO** | **ZrO2** | **BaO** | **PbO2** |
| --- | --- | --- | --- | --- | --- | --- | --- | --- | --- | --- | --- | --- | --- | --- | --- | --- | --- | --- | --- | --- | --- |
| Point 1 | 0,00 | 2,14 | 7,90 | 36,85 | 0,83 | 0,56 | 1,83 | 31,94 | 0,21 | 0,00 | 0,08 | 1,84 | 0,01 | 15,38 | 0,04 | 0,02 | 0,00 | 0,08 | 0,09 | 0,02 | 0,19 |
| Point 2 | 0,34 | 1,36 | 4,55 | 52,39 | 0,34 | 0,35 | 1,21 | 20,82 | 0,14 | 0,00 | 0,04 | 0,96 | 0,00 | 17,20 | 0,04 | 0,01 | 0,01 | 0,07 | 0,00 | 0,00 | 0,18 |
| Point 3 | 1,43 | 0,05 | 0,72 | 60,48 | 0,00 | 0,05 | 0,13 | 15,66 | 0,03 | 0,01 | 0,01 | 0,29 | 0,01 | 20,88 | 0,04 | 0,01 | 0,04 | 0,06 | 0,01 | 0,01 | 0,10 |
| Point 4 | 0,00 | 1,15 | 2,94 | 42,26 | 0,57 | 0,41 | 1,25 | 40,53 | 0,16 | 0,03 | 0,55 | 2,15 | 0,01 | 7,55 | 0,03 | 0,06 | 0,04 | 0,07 | 0,00 | 0,06 | 0,15 |
| Point 5 | 0,68 | 1,01 | 2,72 | 46,67 | 0,11 | 0,23 | 0,61 | 24,33 | 0,04 | 0,18 | 0,02 | 0,90 | 0,00 | 22,14 | 0,04 | 0,03 | 0,02 | 0,10 | 0,01 | 0,03 | 0,13 |
| Point 6 | 0,00 | 2,66 | 10,35 | 38,69 | 0,79 | 0,64 | 2,79 | 33,02 | 0,38 | 0,01 | 0,10 | 3,44 | 0,01 | 6,90 | 0,03 | 0,03 | 0,01 | 0,06 | 0,00 | 0,04 | 0,08 |

Supplementary Table S7. μEDXRF single-spot analyses on fragment 6.

| **Fragment 6** | **Na2O** | **MgO** | **Al2O3** | **SiO2** | **P2O5** | **SO3** | **-Cl** | **K2O** | **CaO** | **TiO2** | **MnO** | **Fe2O3** | **CoO** | **CuO** | **ZnO** | **As2O3** | **SrO** | **BaO** | **PbO2** |
| --- | --- | --- | --- | --- | --- | --- | --- | --- | --- | --- | --- | --- | --- | --- | --- | --- | --- | --- | --- |
| Point 1 | 0,12 | 1,17 | 5,45 | 19,91 | 0,20 | 0,15 | 0,04 | 1,08 | 39,02 | 0,31 | 0,33 | 31,75 | 0,10 | 0,04 | 0,05 | 0,04 | 0,10 | 0,04 | 0,08 |
| Point 2 | 0,02 | 1,40 | 1,42 | 5,96 | 0,03 | 0,24 | 0,00 | 0,27 | 79,75 | 0,32 | 0,07 | 9,54 | 0,06 | 0,05 | 0,02 | 0,02 | 0,69 | 0,05 | 0,10 |
| Point 3 | 0,00 | 1,90 | 2,79 | 13,49 | 0,15 | 0,27 | 0,02 | 0,45 | 69,25 | 0,15 | 0,07 | 10,76 | 0,07 | 0,02 | 0,02 | 0,02 | 0,48 | 0,03 | 0,06 |
| Point 4 | 1,07 | 2,03 | 3,37 | 46,65 | 0,00 | 0,17 | 0,16 | 1,16 | 24,75 | 0,12 | 0,03 | 8,65 | 0,04 | 11,51 | 0,02 | 0,01 | 0,18 | 0,02 | 0,07 |
| Point 5 | 0,00 | 3,33 | 3,35 | 44,00 | 0,00 | 0,26 | 0,07 | 3,72 | 24,03 | 0,16 | 0,14 | 19,59 | 0,07 | 1,00 | 0,01 | 0,03 | 0,11 | 0,00 | 0,12 |
| Point 6 | 0,15 | 2,54 | 4,12 | 35,44 | 0,13 | 0,17 | 0,03 | 1,03 | 31,82 | 0,12 | 0,05 | 23,16 | 0,08 | 0,82 | 0,01 | 0,03 | 0,15 | 0,00 | 0,14 |
| Point 7 | 0,00 | 2,57 | 2,85 | 11,69 | 0,31 | 0,23 | 0,05 | 0,42 | 53,08 | 0,12 | 0,14 | 28,13 | 0,08 | 0,04 | 0,02 | 0,02 | 0,11 | 0,04 | 0,10 |
| Point 8 | 0,04 | 0,74 | 2,34 | 24,27 | 0,00 | 0,23 | 0,07 | 0,64 | 59,85 | 0,29 | 0,02 | 1,38 | 0,01 | 8,54 | 0,01 | 0,02 | 1,37 | 0,02 | 0,13 |
| Point 9 | 0,00 | 0,73 | 2,01 | 8,97 | 0,00 | 0,25 | 0,02 | 0,28 | 83,97 | 0,09 | 0,04 | 1,69 | 0,03 | 0,06 | 0,01 | 0,02 | 1,65 | 0,03 | 0,15 |
| Point 10 | 0,00 | 3,01 | 3,70 | 29,12 | 0,00 | 0,00 | 0,03 | 3,75 | 41,95 | 0,28 | 0,08 | 17,73 | 0,07 | 0,02 | 0,01 | 0,01 | 0,08 | 0,05 | 0,09 |
| Point 11 | 0,68 | 1,73 | 3,29 | 39,42 | 0,00 | 0,14 | 0,13 | 2,97 | 32,09 | 0,18 | 0,07 | 11,59 | 0,05 | 7,43 | 0,02 | 0,02 | 0,08 | 0,02 | 0,08 |
| Point 12 | 0,09 | 0,95 | 2,31 | 14,12 | 0,00 | 0,23 | 0,01 | 0,38 | 78,25 | 0,11 | 0,04 | 2,29 | 0,03 | 0,01 | 0,01 | 0,01 | 1,02 | 0,02 | 0,09 |
| Point 13 | 0,00 | 2,72 | 4,34 | 31,61 | 0,02 | 0,11 | 0,05 | 4,85 | 36,47 | 0,28 | 0,21 | 18,94 | 0,07 | 0,04 | 0,01 | 0,02 | 0,12 | 0,04 | 0,11 |
| Point 14 | 0,00 | 1,05 | 5,73 | 26,32 | 0,30 | 0,25 | 0,11 | 2,87 | 38,51 | 0,59 | 0,19 | 23,67 | 0,08 | 0,03 | 0,02 | 0,02 | 0,11 | 0,04 | 0,10 |
| Point 15 | 0,00 | 1,61 | 2,91 | 10,59 | 0,18 | 0,20 | 0,03 | 0,63 | 51,86 | 0,21 | 0,25 | 31,06 | 0,11 | 0,02 | 0,05 | 0,03 | 0,13 | 0,03 | 0,10 |
| Point 16 | 0,00 | 1,62 | 6,56 | 21,26 | 0,19 | 0,06 | 0,05 | 1,08 | 48,80 | 0,42 | 0,48 | 19,12 | 0,05 | 0,03 | 0,03 | 0,02 | 0,11 | 0,04 | 0,06 |
| Point 17 | 0,00 | 1,24 | 2,34 | 9,67 | 0,04 | 0,30 | 0,00 | 0,24 | 81,48 | 0,09 | 0,09 | 3,05 | 0,02 | 0,06 | 0,01 | 0,01 | 1,21 | 0,05 | 0,08 |
| Point 18 | 0,00 | 1,13 | 6,11 | 23,54 | 0,15 | 0,12 | 0,04 | 0,84 | 38,55 | 0,27 | 0,20 | 28,65 | 0,09 | 0,03 | 0,05 | 0,03 | 0,12 | 0,01 | 0,09 |

Supplementary Table S8. μEDXRF single-spot analyses on fragment 7.

| **Fragment 7** | **Na2O** | **MgO** | **Al2O3** | **SiO2** | **P2O5** | **SO3** | **-Cl** | **K2O** | **CaO** | **TiO2** | **MnO** | **Fe2O3** | **CoO** | **CuO** | **ZnO** | **As2O3** | **SrO** | **BaO** | **PbO2** |
| --- | --- | --- | --- | --- | --- | --- | --- | --- | --- | --- | --- | --- | --- | --- | --- | --- | --- | --- | --- |
| Point 1 | 0,72 | 0,34 | 1,65 | 52,45 | 0,00 | 0,48 | 0,17 | 0,36 | 20,32 | 0,04 | 0,02 | 0,37 | 0,00 | 22,77 | 0,05 | 0,01 | 0,13 | 0,02 | 0,10 |
| Point 2 | 0,00 | 1,45 | 5,83 | 55,21 | 0,18 | 0,65 | 0,10 | 1,11 | 19,59 | 0,18 | 0,07 | 1,55 | 0,01 | 13,75 | 0,04 | 0,00 | 0,06 | 0,04 | 0,18 |
| Point 3 | 1,04 | 0,45 | 3,36 | 58,82 | 0,00 | 0,26 | 0,14 | 1,27 | 16,81 | 0,11 | 0,04 | 0,95 | 0,00 | 16,43 | 0,04 | 0,03 | 0,10 | 0,01 | 0,13 |
| Point 4 | 0,05 | 1,82 | 7,20 | 46,48 | 0,22 | 0,86 | 0,19 | 2,15 | 31,89 | 0,37 | 0,10 | 2,74 | 0,00 | 5,61 | 0,03 | 0,01 | 0,14 | 0,01 | 0,13 |

Supplementary Table S9. μEDXRF single-spot analyses on fragment 8.

| **Fragment 8** | **Na2O** | **MgO** | **Al2O3** | **SiO2** | **P2O5** | **SO3** | **-Cl** | **K2O** | **CaO** | **TiO2** | **Cr2O3** | **MnO** | **Fe2O3** | **CoO** | **CuO** | **ZnO** | **As2O3** | **SrO** | **BaO** | **PbO2** |
| --- | --- | --- | --- | --- | --- | --- | --- | --- | --- | --- | --- | --- | --- | --- | --- | --- | --- | --- | --- | --- |
| Point 1 | 0,00 | 1,60 | 2,84 | 10,90 | 0,07 | 0,19 | 0,02 | 0,31 | 55,70 | 0,17 | 0,01 | 0,24 | 27,36 | 0,07 | 0,10 | 0,03 | 0,08 | 0,18 | 0,04 | 0,09 |
| Point 2 | 0,00 | 1,85 | 3,40 | 10,05 | 0,00 | 0,10 | 0,00 | 0,37 | 63,76 | 0,11 | 0,01 | 0,10 | 19,22 | 0,07 | 0,05 | 0,02 | 0,09 | 0,53 | 0,02 | 0,25 |
| Point 3 | 0,00 | 0,60 | 1,86 | 7,51 | 0,00 | 0,12 | 0,19 | 0,39 | 47,38 | 0,14 | 0,02 | 0,08 | 41,25 | 0,08 | 0,05 | 0,02 | 0,02 | 0,15 | 0,02 | 0,13 |
| Point 4 | 0,00 | 1,10 | 2,56 | 8,35 | 0,02 | 0,01 | 0,01 | 0,32 | 54,13 | 0,15 | 0,01 | 0,07 | 32,54 | 0,09 | 0,11 | 0,02 | 0,07 | 0,14 | 0,02 | 0,31 |
| Point 5 | 0,00 | 1,13 | 3,12 | 10,97 | 0,05 | 0,17 | 0,00 | 0,38 | 66,32 | 0,14 | 0,00 | 0,04 | 16,43 | 0,07 | 0,28 | 0,01 | 0,04 | 0,58 | 0,03 | 0,22 |
| Point 6 | 0,00 | 0,47 | 2,04 | 17,61 | 0,00 | 0,24 | 0,03 | 0,45 | 61,07 | 0,11 | 0,01 | 0,04 | 10,94 | 0,08 | 5,82 | 0,02 | 0,04 | 0,78 | 0,01 | 0,25 |
| Point 7 | 0,01 | 0,62 | 1,24 | 5,72 | 0,00 | 0,17 | 0,04 | 0,16 | 80,64 | 0,08 | 0,01 | 0,16 | 8,70 | 0,06 | 0,62 | 0,01 | 0,07 | 1,14 | 0,06 | 0,49 |
| Point 8 | 0,00 | 0,20 | 1,78 | 6,62 | 0,00 | 0,12 | 0,04 | 0,47 | 77,37 | 0,23 | 0,00 | 0,15 | 10,38 | 0,06 | 0,70 | 0,02 | 0,05 | 1,49 | 0,03 | 0,29 |
| Point 9 | 0,14 | 0,17 | 1,47 | 30,90 | 0,00 | 0,26 | 0,06 | 0,54 | 41,18 | 0,09 | 0,00 | 0,03 | 1,66 | 0,02 | 22,13 | 0,05 | 0,02 | 1,14 | 0,00 | 0,14 |
| Point 10 | 0,00 | 7,58 | 1,66 | 4,21 | 0,05 | 0,14 | 0,35 | 0,10 | 31,52 | 0,01 | 0,01 | 0,64 | 52,69 | 0,18 | 0,17 | 0,01 | 0,00 | 0,48 | 0,04 | 0,17 |
| Point 11 | 0,00 | 0,76 | 3,59 | 11,62 | 0,09 | 0,30 | 0,07 | 0,59 | 71,65 | 0,20 | 0,00 | 0,08 | 9,78 | 0,05 | 0,25 | 0,01 | 0,03 | 0,71 | 0,04 | 0,17 |
| Point 12 | 0,00 | 0,50 | 2,06 | 7,97 | 0,02 | 0,17 | 0,04 | 0,40 | 74,96 | 0,18 | 0,00 | 0,14 | 11,81 | 0,05 | 0,30 | 0,03 | 0,06 | 1,01 | 0,03 | 0,27 |
| Point 13 | 0,00 | 0,82 | 3,46 | 10,34 | 0,00 | 0,13 | 0,00 | 0,41 | 80,15 | 0,18 | 0,00 | 0,04 | 2,86 | 0,04 | 0,04 | 0,02 | 0,04 | 1,11 | 0,04 | 0,33 |
| Point 14 | 0,00 | 1,27 | 2,06 | 6,70 | 0,04 | 0,07 | 0,04 | 0,29 | 47,74 | 0,11 | 0,02 | 0,14 | 40,74 | 0,12 | 0,08 | 0,02 | 0,03 | 0,17 | 0,03 | 0,35 |
| Point 15 | 0,00 | 1,67 | 6,98 | 21,56 | 0,29 | 0,11 | 0,02 | 1,00 | 51,91 | 0,24 | 0,00 | 0,09 | 15,42 | 0,08 | 0,10 | 0,02 | 0,03 | 0,31 | 0,02 | 0,16 |
| Point 16 | 0,00 | 2,21 | 3,38 | 24,91 | 0,00 | 0,02 | 0,06 | 4,07 | 39,19 | 0,24 | 0,01 | 0,07 | 23,59 | 0,11 | 1,78 | 0,01 | 0,03 | 0,13 | 0,06 | 0,14 |
| Point 17 | 0,30 | 0,65 | 1,64 | 44,88 | 0,00 | 0,00 | 0,24 | 0,93 | 23,85 | 0,05 | 0,01 | 0,04 | 14,33 | 0,06 | 12,81 | 0,03 | 0,00 | 0,06 | 0,04 | 0,09 |
| Point 18 | 0,00 | 1,12 | 2,51 | 8,27 | 0,03 | 0,21 | 0,00 | 0,37 | 82,00 | 0,11 | 0,01 | 0,06 | 4,19 | 0,05 | 0,02 | 0,02 | 0,02 | 0,88 | 0,06 | 0,08 |
| Point 19 | 0,00 | 0,80 | 5,23 | 25,05 | 0,32 | 0,03 | 0,02 | 0,97 | 40,25 | 0,35 | 0,03 | 0,33 | 26,20 | 0,07 | 0,03 | 0,05 | 0,02 | 0,12 | 0,03 | 0,11 |
| Point 20 | 0,39 | 0,76 | 1,78 | 41,82 | 0,00 | 0,02 | 0,17 | 1,20 | 26,53 | 0,06 | 0,01 | 0,04 | 15,84 | 0,06 | 11,11 | 0,03 | 0,01 | 0,06 | 0,03 | 0,09 |
| Point 21 | 0,00 | 1,12 | 1,11 | 4,45 | 0,07 | 0,09 | 0,14 | 0,26 | 53,51 | 0,12 | 0,02 | 0,06 | 38,46 | 0,09 | 0,10 | 0,02 | 0,03 | 0,18 | 0,03 | 0,14 |

Supplementary Table S10. μEDXRF single-spot analyses on fragment 9.

| **Fragment 9** | **Na2O** | **MgO** | **Al2O3** | **SiO2** | **P2O5** | **SO3** | **K2O** | **CaO** | **TiO2** | **Cr2O3** | **MnO** | **Fe2O3** | **CoO** | **CuO** | **ZnO** | **BaO** | **Rb2O** | **SrO** | **As2O3** | **HgO** | **PbO2** |
| --- | --- | --- | --- | --- | --- | --- | --- | --- | --- | --- | --- | --- | --- | --- | --- | --- | --- | --- | --- | --- | --- |
| Point 1 | 0,00 | 1,50 | 3,19 | 9,71 | 0,39 | 0,30 | 0,29 | 68,44 | 0,08 | 0,00 | 0,05 | 14,58 | 0,09 | 0,03 | 0,01 | 0,03 | 0,00 | 0,66 | 0,01 | 0,07 | 0,56 |
| Point 2 | 0,00 | 1,88 | 4,04 | 11,98 | 0,30 | 0,26 | 0,42 | 63,68 | 0,12 | 0,00 | 0,08 | 16,07 | 0,07 | 0,02 | 0,01 | 0,02 | 0,01 | 0,35 | 0,03 | 0,61 | 0,05 |
| Point 3 | 0,00 | 2,14 | 5,31 | 30,23 | 0,90 | 1,71 | 1,38 | 52,42 | 0,27 | 0,01 | 0,05 | 4,82 | 0,02 | 0,04 | 0,04 | 0,04 | 0,00 | 0,36 | 0,01 | 0,07 | 0,17 |
| Point 4 | 0,00 | 1,52 | 3,07 | 9,81 | 0,25 | 0,37 | 0,27 | 71,48 | 0,08 | 0,01 | 0,04 | 10,88 | 0,06 | 0,03 | 0,01 | 0,04 | 0,00 | 0,71 | 0,00 | 1,03 | 0,34 |
| Point 5(33) | 0,05 | 2,32 | 3,02 | 9,74 | 0,61 | 0,00 | 0,28 | 47,77 | 0,11 | 0,02 | 0,01 | 35,16 | 0,08 | 0,03 | 0,10 | 0,06 | 0,07 | 0,05 | 0,01 | 0,38 | 0,12 |
| Point 6 | 0,00 | 1,77 | 2,54 | 9,90 | 0,26 | 0,19 | 0,40 | 76,75 | 0,08 | 0,00 | 0,09 | 6,81 | 0,06 | 0,03 | 0,01 | 0,04 | 0,00 | 0,69 | 0,03 | 0,19 | 0,19 |
| Point 7 | 1,00 | 1,05 | 3,69 | 47,99 | 0,20 | 0,05 | 0,52 | 23,57 | 0,19 | 0,03 | 0,02 | 1,57 | 0,01 | 19,36 | 0,03 | 0,00 | 0,00 | 0,62 | 0,01 | 0,00 | 0,07 |
| Point 8 | 0,05 | 2,52 | 3,94 | 13,37 | 0,49 | 0,12 | 0,49 | 66,85 | 0,16 | 0,01 | 0,27 | 10,57 | 0,05 | 0,15 | 0,01 | 0,06 | 0,00 | 0,77 | 0,02 | 0,00 | 0,10 |
| Point 9 | 0,00 | 1,67 | 3,13 | 9,25 | 0,49 | 0,12 | 0,35 | 67,37 | 0,09 | 0,00 | 0,43 | 16,03 | 0,07 | 0,11 | 0,02 | 0,04 | 0,00 | 0,62 | 0,03 | 0,01 | 0,18 |
| Point 10 | 0,00 | 1,23 | 1,99 | 7,10 | 0,34 | 0,27 | 0,25 | 79,97 | 0,09 | 0,00 | 0,08 | 6,97 | 0,06 | 0,34 | 0,02 | 0,05 | 0,01 | 1,02 | 0,03 | 0,06 | 0,10 |
| Point 11 | 0,00 | 2,15 | 5,76 | 22,71 | 0,54 | 0,17 | 0,67 | 54,83 | 0,17 | 0,01 | 0,13 | 12,26 | 0,07 | 0,03 | 0,01 | 0,04 | 0,00 | 0,34 | 0,02 | 0,04 | 0,05 |
| Point 12 | 0,00 | 2,20 | 3,51 | 10,78 | 0,39 | 0,11 | 0,33 | 69,04 | 0,11 | 0,00 | 0,25 | 10,00 | 0,04 | 0,09 | 0,01 | 0,05 | 0,00 | 0,87 | 0,04 | 1,96 | 0,21 |
| Point 13 | 0,00 | 1,47 | 2,15 | 7,29 | 0,36 | 0,19 | 0,22 | 77,93 | 0,08 | 0,00 | 0,08 | 3,88 | 0,03 | 0,02 | 0,01 | 0,04 | 0,00 | 1,15 | 0,04 | 4,89 | 0,18 |
| Point 14 | 0,00 | 2,64 | 3,97 | 14,62 | 0,61 | 0,00 | 0,47 | 64,28 | 0,13 | 0,01 | 0,04 | 12,29 | 0,02 | 0,60 | 0,01 | 0,06 | 0,04 | 0,08 | 0,00 | 0,00 | 0,13 |
| Point 15 | 0,00 | 1,56 | 2,33 | 7,82 | 0,36 | 0,24 | 0,24 | 78,01 | 0,08 | 0,00 | 0,08 | 5,00 | 0,03 | 0,03 | 0,01 | 0,05 | 0,00 | 1,56 | 0,05 | 2,41 | 0,15 |
| Point 16 | 0,00 | 1,51 | 2,14 | 6,70 | 0,24 | 0,50 | 0,25 | 73,24 | 0,10 | 0,00 | 0,05 | 5,87 | 0,03 | 0,13 | 0,02 | 0,04 | 0,00 | 0,96 | 0,05 | 8,04 | 0,12 |
| Point 17 | 0,00 | 1,88 | 3,15 | 10,51 | 0,29 | 0,41 | 0,34 | 71,39 | 0,10 | 0,00 | 0,04 | 8,45 | 0,05 | 0,07 | 0,02 | 0,03 | 0,01 | 0,64 | 0,03 | 2,47 | 0,12 |
| Point 18 | 0,00 | 1,95 | 2,66 | 14,90 | 0,37 | 0,00 | 0,38 | 54,70 | 0,10 | 0,00 | 0,28 | 23,79 | 0,09 | 0,31 | 0,01 | 0,04 | 0,01 | 0,25 | 0,03 | 0,01 | 0,10 |
| Point 19 | 0,00 | 1,53 | 2,75 | 10,20 | 0,42 | 0,08 | 0,32 | 64,39 | 0,07 | 0,00 | 0,18 | 19,14 | 0,10 | 0,17 | 0,01 | 0,02 | 0,00 | 0,44 | 0,02 | 0,00 | 0,14 |
| Point 20 | 0,00 | 2,44 | 8,01 | 24,49 | 0,42 | 0,09 | 1,27 | 59,24 | 0,25 | 0,00 | 0,10 | 2,69 | 0,01 | 0,15 | 0,01 | 0,03 | 0,00 | 0,72 | 0,01 | 0,00 | 0,07 |
| Point 21 | 0,18 | 1,91 | 5,64 | 31,88 | 0,40 | 0,19 | 0,74 | 47,38 | 0,16 | 0,00 | 0,05 | 2,05 | 0,01 | 8,49 | 0,02 | 0,00 | 0,00 | 0,81 | 0,02 | 0,00 | 0,06 |
| Point 22 | 0,26 | 3,04 | 4,34 | 16,01 | 0,37 | 0,10 | 0,47 | 54,81 | 0,09 | 0,01 | 0,26 | 19,39 | 0,09 | 0,10 | 0,01 | 0,06 | 0,00 | 0,38 | 0,03 | 0,00 | 0,18 |
| Point 23 | 0,16 | 1,74 | 3,12 | 10,31 | 0,33 | 0,21 | 0,45 | 70,02 | 0,10 | 0,00 | 0,10 | 12,78 | 0,07 | 0,02 | 0,01 | 0,02 | 0,05 | 0,39 | 0,03 | 0,02 | 0,08 |
| Point 24 | 0,00 | 1,42 | 2,28 | 11,61 | 0,28 | 0,19 | 0,25 | 71,04 | 0,06 | 0,01 | 0,09 | 11,39 | 0,06 | 0,03 | 0,01 | 0,04 | 0,00 | 0,56 | 0,03 | 0,47 | 0,17 |
| Point 25 | 0,07 | 1,24 | 2,14 | 7,73 | 0,36 | 0,14 | 0,25 | 65,20 | 0,08 | 0,00 | 0,15 | 21,80 | 0,10 | 0,07 | 0,11 | 0,04 | 0,00 | 0,39 | 0,04 | 0,00 | 0,11 |
| Point 26 | 0,00 | 1,85 | 2,34 | 7,67 | 0,29 | 0,20 | 0,32 | 77,52 | 0,06 | 0,00 | 0,09 | 5,24 | 0,03 | 0,07 | 0,01 | 0,05 | 0,00 | 1,12 | 0,03 | 2,90 | 0,20 |
| Point 27 | 0,00 | 2,19 | 2,45 | 9,03 | 0,43 | 0,00 | 0,29 | 45,19 | 0,12 | 0,02 | 0,10 | 39,75 | 0,11 | 0,06 | 0,01 | 0,03 | 0,01 | 0,13 | 0,01 | 0,02 | 0,07 |
| Point 28 | 0,75 | 1,69 | 6,34 | 20,51 | 2,02 | 0,00 | 1,02 | 47,54 | 0,33 | 0,01 | 0,21 | 19,26 | 0,05 | 0,03 | 0,02 | 0,05 | 0,02 | 0,11 | 0,02 | 0,01 | 0,01 |
| Point 29 | 5,22 | 1,64 | 5,73 | 16,80 | 7,35 | 0,00 | 0,78 | 47,35 | 0,28 | 0,01 | 0,15 | 14,37 | 0,04 | 0,04 | 0,02 | 0,02 | 0,00 | 0,07 | 0,03 | 0,00 | 0,10 |
| Point 30 | 0,00 | 1,52 | 7,68 | 19,51 | 0,52 | 0,00 | 0,93 | 44,93 | 0,34 | 0,01 | 0,09 | 24,18 | 0,07 | 0,01 | 0,04 | 0,01 | 0,00 | 0,09 | 0,00 | 0,00 | 0,07 |
| Point 31 | 0,00 | 1,62 | 7,21 | 18,60 | 0,46 | 0,00 | 0,84 | 37,81 | 0,33 | 0,00 | 2,38 | 29,73 | 0,35 | 0,02 | 0,05 | 0,32 | 0,01 | 0,17 | 0,04 | 0,00 | 0,06 |
| Point 32 | 0,00 | 1,74 | 7,21 | 20,09 | 0,51 | 0,00 | 0,99 | 39,87 | 0,39 | 0,01 | 0,24 | 28,57 | 0,09 | 0,02 | 0,04 | 0,01 | 0,00 | 0,13 | 0,03 | 0,00 | 0,06 |
| Point 34 | 0,00 | 1,56 | 2,28 | 7,35 | 0,13 | 0,00 | 0,18 | 20,42 | 0,06 | 0,03 | 0,07 | 67,37 | 0,18 | 0,06 | 0,01 | 0,03 | 0,01 | 0,12 | 0,04 | 0,02 | 0,07 |
| Point 35 | 6,01 | 1,39 | 1,56 | 5,31 | 11,07 | 0,00 | 0,31 | 46,05 | 0,09 | 0,01 | 0,04 | 27,78 | 0,08 | 0,03 | 0,02 | 0,03 | 0,03 | 0,10 | 0,03 | 0,00 | 0,06 |
| Point 36 | 6,61 | 1,63 | 1,88 | 7,21 | 10,35 | 0,00 | 0,24 | 46,36 | 0,10 | 0,01 | 0,04 | 25,22 | 0,06 | 0,11 | 0,01 | 0,03 | 0,00 | 0,08 | 0,00 | 0,00 | 0,07 |
| Point 37 | 0,00 | 1,81 | 2,18 | 7,54 | 0,31 | 0,00 | 0,23 | 53,61 | 0,13 | 0,02 | 0,05 | 33,73 | 0,08 | 0,03 | 0,01 | 0,04 | 0,00 | 0,15 | 0,01 | 0,00 | 0,06 |
| Point 38 | 3,11 | 1,03 | 0,90 | 3,58 | 4,01 | 0,25 | 0,15 | 83,38 | 0,10 | 0,00 | 0,04 | 2,04 | 0,03 | 0,02 | 0,01 | 0,06 | 0,00 | 1,21 | 0,02 | 0,00 | 0,06 |
| Point 39 | 2,70 | 1,48 | 4,01 | 14,65 | 5,12 | 0,06 | 1,04 | 63,55 | 0,14 | 0,00 | 0,03 | 6,00 | 0,04 | 0,31 | 0,01 | 0,04 | 0,00 | 0,71 | 0,02 | 0,01 | 0,09 |
| Point 40 | 0,00 | 1,81 | 2,50 | 9,71 | 0,35 | 0,13 | 0,24 | 80,13 | 0,08 | 0,00 | 0,04 | 3,91 | 0,04 | 0,01 | 0,01 | 0,03 | 0,00 | 0,94 | 0,01 | 0,00 | 0,05 |
| Point 41 | 0,11 | 1,56 | 3,20 | 10,20 | 0,28 | 0,28 | 0,41 | 74,49 | 0,14 | 0,00 | 0,05 | 8,28 | 0,05 | 0,01 | 0,01 | 0,05 | 0,02 | 0,76 | 0,02 | 0,00 | 0,07 |
| Point 42 | 6,61 | 1,90 | 6,47 | 27,89 | 8,66 | 0,00 | 2,87 | 32,26 | 0,15 | 0,00 | 0,08 | 12,62 | 0,05 | 0,19 | 0,01 | 0,05 | 0,00 | 0,09 | 0,01 | 0,03 | 0,04 |
| Point 43 | 3,46 | 1,88 | 3,48 | 18,37 | 5,12 | 0,00 | 2,20 | 43,46 | 0,11 | 0,01 | 0,06 | 20,78 | 0,08 | 0,27 | 0,02 | 0,04 | 0,00 | 0,14 | 0,08 | 0,01 | 0,45 |
| Point 44 | 2,82 | 2,32 | 3,88 | 26,58 | 5,28 | 0,00 | 2,02 | 30,78 | 0,09 | 0,01 | 0,08 | 20,57 | 0,09 | 5,23 | 0,01 | 0,03 | 0,00 | 0,13 | 0,02 | 0,01 | 0,05 |
| Point 45 | 3,72 | 1,35 | 3,61 | 47,07 | 6,99 | 0,00 | 2,76 | 23,93 | 0,16 | 0,00 | 0,04 | 9,79 | 0,05 | 0,28 | 0,03 | 0,05 | 0,00 | 0,07 | 0,02 | 0,00 | 0,05 |
| Point 46 | 1,83 | 2,03 | 7,56 | 36,14 | 3,75 | 0,20 | 2,35 | 33,58 | 0,29 | 0,01 | 0,08 | 11,87 | 0,04 | 0,06 | 0,01 | 0,03 | 0,00 | 0,12 | 0,01 | 0,00 | 0,04 |
| Point 47 | 2,95 | 2,55 | 4,07 | 25,16 | 5,01 | 0,00 | 3,23 | 41,50 | 0,11 | 0,01 | 0,09 | 14,61 | 0,05 | 0,36 | 0,01 | 0,07 | 0,00 | 0,12 | 0,02 | 0,00 | 0,06 |
| Point 48 | 2,40 | 2,21 | 3,79 | 36,82 | 3,84 | 0,00 | 2,10 | 31,05 | 0,09 | 0,01 | 0,03 | 8,12 | 0,04 | 9,29 | 0,02 | 0,03 | 0,00 | 0,12 | 0,02 | 0,00 | 0,03 |
| Point 49 | 3,02 | 1,68 | 2,53 | 40,01 | 4,14 | 0,00 | 2,11 | 27,56 | 0,09 | 0,01 | 0,03 | 6,53 | 0,04 | 12,00 | 0,03 | 0,03 | 0,00 | 0,15 | 0,02 | 0,00 | 0,04 |
| Point 50 | 1,01 | 2,60 | 3,10 | 31,10 | 2,90 | 0,00 | 3,17 | 35,16 | 0,20 | 0,00 | 0,04 | 18,68 | 0,09 | 1,65 | 0,01 | 0,04 | 0,00 | 0,17 | 0,02 | 0,00 | 0,06 |
| Point 51 | 1,13 | 2,23 | 2,71 | 24,98 | 2,98 | 0,00 | 2,45 | 26,80 | 0,08 | 0,01 | 0,02 | 31,69 | 0,09 | 4,43 | 0,04 | 0,03 | 0,00 | 0,19 | 0,05 | 0,01 | 0,07 |
| Point 52 | 1,64 | 2,08 | 2,54 | 34,65 | 2,47 | 0,00 | 2,66 | 34,23 | 0,17 | 0,03 | 0,04 | 13,40 | 0,07 | 5,70 | 0,02 | 0,02 | 0,00 | 0,21 | 0,02 | 0,00 | 0,06 |

Supplementary Table S11. μEDXRF single-spot analyses on fragment 10.

| **Fragment 10** | **Na2O** | **MgO** | **Al2O3** | **SiO2** | **P2O5** | **SO3** | **-Cl** | **K2O** | **CaO** | **TiO2** | **V2O5** | **Cr2O3** | **MnO** | **Fe2O3** | **CoO** | **CuO** | **ZnO** | **As2O3** | **Rb2O** | **Y2O3** | **BaO** | **HgO** | **SrO** | **PbO2** |
| --- | --- | --- | --- | --- | --- | --- | --- | --- | --- | --- | --- | --- | --- | --- | --- | --- | --- | --- | --- | --- | --- | --- | --- | --- |
| Point 1 | 0,32 | 1,03 | 2,35 | 34,69 | 0,20 | 0,35 | 0,04 | 2,32 | 27,52 | 0,09 | 0,02 | 0,00 | 0,04 | 19,80 | 0,13 | 10,78 | 0,02 | 0,02 | 0,00 | 0,00 | 0,04 | 0,00 | 0,15 | 0,07 |
| Point 2 | 0,00 | 2,17 | 3,21 | 21,28 | 0,32 | 0,51 | 0,01 | 3,48 | 41,81 | 0,12 | 0,00 | 0,01 | 0,03 | 26,39 | 0,12 | 0,09 | 0,01 | 0,02 | 0,01 | 0,00 | 0,12 | 0,00 | 0,20 | 0,09 |
| Point 3 | 1,36 | 1,00 | 3,19 | 42,59 | 0,21 | 0,25 | 0,05 | 2,28 | 28,41 | 0,13 | 0,00 | 0,02 | 0,03 | 9,57 | 0,05 | 10,50 | 0,03 | 0,02 | 0,01 | 0,00 | 0,03 | 0,01 | 0,19 | 0,06 |
| Point 4 | 0,00 | 2,50 | 4,79 | 25,92 | 0,45 | 0,45 | 0,03 | 3,98 | 40,53 | 0,15 | 0,00 | 0,01 | 0,06 | 18,90 | 0,08 | 1,82 | 0,01 | 0,02 | 0,00 | 0,00 | 0,07 | 0,00 | 0,14 | 0,09 |
| Point 5 | 0,00 | 1,65 | 6,75 | 26,63 | 0,97 | 0,41 | 0,04 | 2,28 | 48,98 | 0,34 | 0,13 | 0,01 | 0,06 | 10,70 | 0,07 | 0,54 | 0,02 | 0,02 | 0,00 | 0,00 | 0,08 | 0,01 | 0,23 | 0,10 |
| Point 6 | 0,50 | 0,79 | 3,33 | 51,34 | 0,22 | 0,29 | 0,06 | 0,90 | 19,24 | 0,09 | 0,00 | 0,02 | 0,02 | 1,55 | 0,01 | 21,32 | 0,04 | 0,01 | 0,12 | 0,00 | 0,01 | 0,01 | 0,08 | 0,05 |
| Point 7 | 0,00 | 1,76 | 4,10 | 33,74 | 0,40 | 0,38 | 0,03 | 3,15 | 35,46 | 0,09 | 0,25 | 0,00 | 0,07 | 18,22 | 0,09 | 1,79 | 0,01 | 0,02 | 0,05 | 0,00 | 0,19 | 0,01 | 0,12 | 0,08 |
| Point 8 | 0,07 | 0,54 | 1,34 | 6,41 | 0,37 | 0,63 | 0,02 | 0,46 | 85,14 | 0,08 | 0,00 | 0,01 | 0,06 | 3,66 | 0,05 | 0,01 | 0,03 | 0,03 | 0,00 | 0,00 | 0,07 | 0,00 | 0,93 | 0,08 |
| Point 9 | 0,00 | 0,59 | 4,29 | 15,24 | 0,57 | 0,56 | 0,03 | 1,03 | 56,03 | 0,54 | 0,03 | 0,01 | 0,22 | 20,35 | 0,04 | 0,03 | 0,04 | 0,06 | 0,03 | 0,00 | 0,05 | 0,00 | 0,17 | 0,10 |
| Point 10 | 0,00 | 0,94 | 3,46 | 11,92 | 0,18 | 7,27 | 0,00 | 0,71 | 51,95 | 0,20 | 0,05 | 0,02 | 0,04 | 5,12 | 0,05 | 0,05 | 0,07 | 0,06 | 0,00 | 0,03 | 0,09 | 13,49 | 0,92 | 3,39 |
| Point 11 | 0,00 | 1,42 | 5,19 | 22,80 | 0,67 | 0,52 | 0,03 | 1,46 | 51,84 | 0,30 | 0,00 | 0,01 | 0,09 | 14,13 | 0,06 | 0,40 | 0,02 | 0,06 | 0,00 | 0,00 | 0,06 | 0,32 | 0,38 | 0,25 |
| Point 12 | 0,00 | 0,97 | 3,41 | 13,54 | 0,46 | 0,50 | 0,01 | 1,00 | 63,14 | 0,13 | 0,00 | 0,01 | 0,07 | 15,58 | 0,08 | 0,32 | 0,02 | 0,05 | 0,00 | 0,00 | 0,03 | 0,02 | 0,41 | 0,26 |
| Point 13 | 0,15 | 1,01 | 1,84 | 6,84 | 0,56 | 0,56 | 0,05 | 0,40 | 40,44 | 0,13 | 0,00 | 0,02 | 0,10 | 47,31 | 0,12 | 0,06 | 0,02 | 0,03 | 0,00 | 0,00 | 0,03 | 0,02 | 0,15 | 0,17 |
| Point 14 | 0,00 | 0,86 | 1,33 | 6,07 | 0,47 | 0,66 | 0,05 | 0,36 | 40,15 | 0,12 | 0,00 | 0,02 | 0,05 | 49,32 | 0,13 | 0,12 | 0,01 | 0,02 | 0,00 | 0,00 | 0,02 | 0,00 | 0,12 | 0,12 |
| Point 15 | 0,00 | 0,81 | 1,45 | 5,97 | 0,54 | 0,87 | 0,06 | 0,44 | 48,03 | 0,14 | 0,00 | 0,02 | 0,09 | 41,03 | 0,11 | 0,08 | 0,03 | 0,03 | 0,03 | 0,00 | 0,03 | 0,02 | 0,11 | 0,10 |
| Point 16 | 0,00 | 0,70 | 4,39 | 13,40 | 0,52 | 0,37 | 0,02 | 0,91 | 57,41 | 0,31 | 0,01 | 0,01 | 0,22 | 21,28 | 0,06 | 0,02 | 0,04 | 0,03 | 0,00 | 0,00 | 0,05 | 0,00 | 0,18 | 0,08 |
| Point 17 | 0,00 | 0,54 | 3,75 | 14,76 | 0,56 | 0,51 | 0,04 | 1,17 | 51,66 | 0,44 | 0,00 | 0,01 | 0,16 | 25,94 | 0,05 | 0,01 | 0,04 | 0,07 | 0,02 | 0,00 | 0,00 | 0,02 | 0,14 | 0,10 |
| Point 18 | 0,00 | 0,84 | 1,60 | 5,08 | 0,42 | 0,60 | 0,01 | 0,39 | 85,15 | 0,11 | 0,00 | 0,01 | 0,05 | 4,64 | 0,04 | 0,02 | 0,02 | 0,03 | 0,01 | 0,00 | 0,04 | 0,00 | 0,86 | 0,11 |
| Point 19 | 0,00 | 0,79 | 1,61 | 10,83 | 0,35 | 0,58 | 0,02 | 0,36 | 80,53 | 0,11 | 0,00 | 0,00 | 0,06 | 3,68 | 0,04 | 0,04 | 0,02 | 0,03 | 0,00 | 0,00 | 0,01 | 0,00 | 0,86 | 0,09 |
| Point 20 | 0,00 | 1,00 | 2,55 | 10,54 | 0,45 | 0,56 | 0,04 | 0,54 | 71,65 | 0,13 | 0,00 | 0,00 | 0,08 | 11,30 | 0,08 | 0,18 | 0,01 | 0,03 | 0,00 | 0,00 | 0,01 | 0,00 | 0,64 | 0,20 |
| Point 21 | 0,00 | 0,69 | 3,27 | 21,91 | 0,47 | 0,71 | 0,04 | 0,89 | 60,12 | 0,36 | 0,00 | 0,00 | 0,10 | 10,28 | 0,06 | 0,38 | 0,00 | 0,03 | 0,00 | 0,00 | 0,02 | 0,04 | 0,52 | 0,09 |
| Point 22 | 0,00 | 0,85 | 2,80 | 8,37 | 0,27 | 10,07 | 0,00 | 0,54 | 48,92 | 0,14 | 0,03 | 0,03 | 0,34 | 3,13 | 0,04 | 0,06 | 0,06 | 0,04 | 0,00 | 0,04 | 0,07 | 18,53 | 1,08 | 4,60 |
| Point 23 | 0,00 | 1,16 | 1,77 | 6,03 | 0,15 | 7,15 | 0,00 | 0,38 | 62,37 | 0,08 | 0,00 | 0,02 | 0,05 | 4,78 | 0,05 | 0,04 | 0,05 | 0,00 | 0,00 | 0,03 | 0,05 | 10,88 | 1,14 | 3,81 |
| Point 24 | 0,00 | 1,14 | 4,71 | 28,86 | 0,60 | 0,55 | 0,03 | 1,00 | 44,44 | 0,19 | 0,00 | 0,01 | 0,13 | 16,97 | 0,07 | 0,34 | 0,02 | 0,09 | 0,00 | 0,00 | 0,02 | 0,04 | 0,40 | 0,40 |
| Point 25 | 0,00 | 1,08 | 3,75 | 12,65 | 0,52 | 0,63 | 0,01 | 0,65 | 59,70 | 0,11 | 0,00 | 0,00 | 0,03 | 18,90 | 0,10 | 0,41 | 0,02 | 0,10 | 0,00 | 0,00 | 0,01 | 0,14 | 0,46 | 0,72 |
| Point 26 | 0,00 | 0,98 | 2,56 | 9,04 | 0,46 | 0,62 | 0,02 | 0,55 | 72,32 | 0,11 | 0,00 | 0,00 | 0,04 | 11,66 | 0,07 | 0,14 | 0,01 | 0,07 | 0,00 | 0,00 | 0,03 | 0,05 | 0,89 | 0,37 |

Supplementary Table S12. μEDXRF single-spot analyses on fragment 11.

| **Fragment 11** | **Na2O** | **MgO** | **Al2O3** | **SiO2** | **P2O5** | **SO3** | **Cl-** | **K2O** | **CaO** | **TiO2** | **MnO** | **Fe2O3** | **CuO** | **ZnO** | **HgO** | **CoO** | **Rb2O** | **V2O5** | **PbO2** | **As2O3** | **SrO** |
| --- | --- | --- | --- | --- | --- | --- | --- | --- | --- | --- | --- | --- | --- | --- | --- | --- | --- | --- | --- | --- | --- |
| Point 1 | 0,00 | 1,77 | 3,75 | 11,67 | 0,54 | 0,42 | 0,00 | 0,50 | 66,52 | 0,13 | 0,12 | 13,30 | 0,68 | 0,01 | 0,03 | 0,06 | 0,00 | 0,00 | 0,07 | 0,01 | 0,42 |
| Point 2 | 0,12 | 1,10 | 2,55 | 15,47 | 0,40 | 0,38 | 0,02 | 0,50 | 46,69 | 0,11 | 0,06 | 27,28 | 4,77 | 0,01 | 0,00 | 0,13 | 0,00 | 0,04 | 0,02 | 0,02 | 0,31 |
| Point 3 | 0,00 | 2,42 | 3,22 | 10,90 | 0,46 | 0,55 | 0,05 | 0,36 | 43,63 | 0,13 | 0,07 | 37,88 | 0,04 | 0,01 | 0,00 | 0,08 | 0,00 | 0,01 | 0,05 | 0,01 | 0,12 |
| Point 4 | 0,00 | 2,24 | 3,95 | 11,61 | 0,53 | 0,60 | 0,04 | 0,41 | 40,09 | 0,15 | 0,04 | 39,77 | 0,28 | 0,01 | 0,01 | 0,09 | 0,00 | 0,01 | 0,06 | 0,02 | 0,12 |
| Point 5 | 0,00 | 2,06 | 6,74 | 18,89 | 0,49 | 0,44 | 0,02 | 0,75 | 37,03 | 0,20 | 0,08 | 32,79 | 0,18 | 0,01 | 0,00 | 0,10 | 0,00 | 0,00 | 0,07 | 0,02 | 0,13 |
| Point 6 | 0,00 | 1,86 | 4,11 | 12,62 | 0,53 | 0,54 | 0,00 | 0,46 | 61,13 | 0,19 | 0,11 | 17,11 | 0,15 | 0,01 | 0,00 | 0,06 | 0,00 | 0,01 | 0,45 | 0,09 | 0,58 |
| Point 7 | 0,00 | 1,86 | 4,09 | 11,32 | 0,48 | 0,44 | 0,00 | 0,42 | 55,00 | 0,13 | 0,18 | 24,56 | 0,10 | 0,02 | 0,01 | 0,10 | 0,01 | 0,01 | 0,67 | 0,11 | 0,49 |
| Point 8 | 0,00 | 1,87 | 2,81 | 8,61 | 0,48 | 0,47 | 0,05 | 0,36 | 34,45 | 0,12 | 0,04 | 50,18 | 0,11 | 0,01 | 0,00 | 0,13 | 0,00 | 0,01 | 0,10 | 0,02 | 0,20 |
| Point 9 | 0,00 | 2,00 | 5,34 | 16,34 | 0,62 | 0,48 | 0,00 | 0,83 | 49,34 | 0,14 | 0,11 | 24,23 | 0,08 | 0,01 | 0,00 | 0,07 | 0,08 | 0,01 | 0,15 | 0,04 | 0,14 |
| Point 10 | 0,00 | 1,50 | 2,88 | 8,49 | 0,50 | 0,60 | 0,00 | 0,34 | 64,07 | 0,15 | 0,10 | 20,10 | 0,03 | 0,01 | 0,00 | 0,09 | 0,00 | 0,00 | 0,52 | 0,08 | 0,52 |
| Point 11 | 0,00 | 4,01 | 5,60 | 34,47 | 0,25 | 0,40 | 0,01 | 4,80 | 33,54 | 0,17 | 0,06 | 15,64 | 0,05 | 0,01 | 0,00 | 0,06 | 0,02 | 0,00 | 0,49 | 0,08 | 0,34 |
| Point 12 | 0,00 | 1,59 | 3,52 | 10,86 | 0,55 | 0,55 | 0,00 | 0,40 | 67,25 | 0,12 | 0,05 | 13,61 | 0,04 | 0,01 | 0,00 | 0,07 | 0,00 | 0,01 | 0,58 | 0,08 | 0,73 |
| Point 13 | 0,00 | 1,80 | 3,68 | 11,96 | 0,48 | 0,55 | 0,00 | 1,01 | 65,03 | 0,15 | 0,11 | 13,14 | 0,03 | 0,02 | 0,05 | 0,06 | 0,00 | 0,00 | 1,10 | 0,13 | 0,69 |
| Point 14 | 0,00 | 1,96 | 3,92 | 13,25 | 0,54 | 0,38 | 0,00 | 0,54 | 62,29 | 0,18 | 0,07 | 15,07 | 0,03 | 0,01 | 0,00 | 0,07 | 0,00 | 0,00 | 1,00 | 0,09 | 0,59 |
| Point 15 | 0,00 | 2,38 | 6,27 | 18,62 | 0,56 | 0,44 | 0,00 | 0,79 | 56,93 | 0,23 | 0,05 | 12,88 | 0,05 | 0,01 | 0,00 | 0,05 | 0,01 | 0,00 | 0,38 | 0,06 | 0,29 |
| Point 16 | 0,00 | 1,71 | 3,34 | 9,77 | 0,50 | 0,41 | 0,00 | 0,32 | 65,13 | 0,39 | 0,08 | 16,82 | 0,04 | 0,02 | 0,00 | 0,08 | 0,00 | 0,00 | 0,72 | 0,10 | 0,57 |
| Point 17 | 0,00 | 1,91 | 9,28 | 24,84 | 0,61 | 0,35 | 0,01 | 1,57 | 34,64 | 0,53 | 0,61 | 25,23 | 0,02 | 0,04 | 0,00 | 0,06 | 0,01 | 0,04 | 0,11 | 0,03 | 0,12 |
| Point 18 | 0,00 | 2,08 | 3,89 | 11,03 | 0,64 | 0,45 | 0,02 | 0,76 | 50,72 | 0,23 | 0,18 | 29,49 | 0,03 | 0,04 | 0,00 | 0,09 | 0,00 | 0,02 | 0,13 | 0,05 | 0,14 |
| Point 19 | 0,00 | 1,72 | 3,89 | 10,25 | 0,51 | 0,32 | 0,00 | 0,33 | 53,97 | 0,11 | 0,01 | 27,65 | 0,04 | 0,03 | 0,00 | 0,10 | 0,00 | 0,00 | 0,78 | 0,13 | 0,17 |
| Point 20 | 0,00 | 1,85 | 4,74 | 12,44 | 0,49 | 0,35 | 0,00 | 0,36 | 50,92 | 0,08 | 0,03 | 27,76 | 0,03 | 0,03 | 0,01 | 0,10 | 0,00 | 0,00 | 0,59 | 0,12 | 0,09 |
| Point 21 | 0,00 | 2,21 | 5,42 | 15,86 | 0,58 | 0,39 | 0,01 | 0,61 | 60,87 | 0,13 | 0,04 | 13,24 | 0,03 | 0,01 | 0,00 | 0,07 | 0,00 | 0,01 | 0,07 | 0,02 | 0,44 |
| Point 22 | 0,00 | 1,62 | 5,23 | 14,19 | 0,45 | 0,48 | 0,02 | 1,31 | 62,49 | 0,15 | 0,04 | 13,22 | 0,05 | 0,01 | 0,03 | 0,07 | 0,00 | 0,01 | 0,12 | 0,03 | 0,47 |
| Point 23 | 0,00 | 1,77 | 4,38 | 14,17 | 0,68 | 0,54 | 0,00 | 0,53 | 56,23 | 0,16 | 0,10 | 20,65 | 0,05 | 0,01 | 0,00 | 0,09 | 0,00 | 0,01 | 0,24 | 0,05 | 0,34 |
| Point 24 | 0,00 | 2,48 | 3,52 | 10,08 | 0,67 | 0,71 | 0,01 | 0,44 | 59,58 | 0,11 | 0,46 | 21,17 | 0,06 | 0,01 | 0,00 | 0,07 | 0,03 | 0,00 | 0,29 | 0,06 | 0,25 |
| Point 25 | 0,00 | 2,24 | 4,29 | 13,64 | 0,57 | 0,48 | 0,00 | 0,50 | 59,07 | 0,12 | 0,15 | 17,44 | 0,05 | 0,01 | 0,01 | 0,08 | 0,00 | 0,00 | 0,68 | 0,11 | 0,55 |
| Point 26 | 0,00 | 1,77 | 5,23 | 16,37 | 0,51 | 0,46 | 0,00 | 0,67 | 50,00 | 0,14 | 0,12 | 23,51 | 0,09 | 0,01 | 0,00 | 0,09 | 0,00 | 0,00 | 0,46 | 0,06 | 0,49 |
| Point 27 | 0,00 | 1,73 | 3,97 | 15,69 | 0,52 | 0,44 | 0,00 | 0,89 | 61,49 | 0,25 | 0,08 | 13,29 | 0,06 | 0,01 | 0,04 | 0,05 | 0,00 | 0,01 | 0,76 | 0,11 | 0,61 |
| Point 28 | 0,07 | 1,89 | 4,55 | 14,26 | 0,56 | 0,57 | 0,00 | 0,78 | 65,66 | 0,18 | 0,05 | 10,01 | 0,04 | 0,01 | 0,00 | 0,05 | 0,01 | 0,00 | 0,67 | 0,09 | 0,56 |
| Point 29 | 0,00 | 2,21 | 3,37 | 10,92 | 0,57 | 0,50 | 0,01 | 0,39 | 57,99 | 0,13 | 0,06 | 23,11 | 0,04 | 0,01 | 0,00 | 0,04 | 0,00 | 0,02 | 0,48 | 0,05 | 0,12 |
| Point 30 | 0,00 | 3,23 | 4,38 | 13,10 | 0,64 | 0,53 | 0,02 | 0,50 | 57,56 | 0,15 | 0,16 | 17,46 | 0,83 | 0,01 | 0,00 | 0,06 | 0,00 | 0,01 | 0,64 | 0,10 | 0,58 |
| Point 31 | 0,00 | 3,40 | 4,37 | 13,75 | 0,53 | 0,45 | 0,03 | 0,50 | 44,31 | 0,11 | 0,43 | 29,77 | 0,05 | 0,02 | 0,06 | 0,08 | 0,00 | 0,00 | 1,24 | 0,11 | 0,81 |
| Point 32 | 0,00 | 2,06 | 5,36 | 15,89 | 0,52 | 0,38 | 0,00 | 0,56 | 50,54 | 0,28 | 0,16 | 23,15 | 0,15 | 0,01 | 0,00 | 0,10 | 0,00 | 0,00 | 0,41 | 0,07 | 0,37 |
| Point 33 | 0,00 | 1,98 | 4,00 | 12,44 | 0,69 | 0,48 | 0,00 | 0,41 | 62,22 | 0,11 | 0,29 | 14,21 | 0,63 | 0,01 | 0,00 | 0,05 | 0,00 | 0,01 | 1,34 | 0,16 | 0,97 |
| Point 34 | 0,00 | 1,93 | 3,89 | 10,92 | 0,50 | 0,48 | 0,04 | 0,39 | 25,77 | 0,12 | 0,03 | 55,48 | 0,05 | 0,01 | 0,02 | 0,14 | 0,01 | 0,01 | 0,11 | 0,01 | 0,11 |
| Point 35 | 0,00 | 1,72 | 4,54 | 15,81 | 0,51 | 0,58 | 0,00 | 0,52 | 58,02 | 1,64 | 0,03 | 15,66 | 0,06 | 0,01 | 0,00 | 0,07 | 0,01 | 0,00 | 0,36 | 0,06 | 0,40 |
| Point 36 | 0,00 | 2,33 | 4,76 | 13,54 | 0,52 | 0,49 | 0,01 | 0,48 | 44,58 | 0,13 | 0,18 | 32,15 | 0,22 | 0,01 | 0,00 | 0,12 | 0,00 | 0,01 | 0,20 | 0,05 | 0,21 |
| Point 37 | 0,00 | 2,59 | 5,43 | 16,39 | 0,49 | 0,50 | 0,00 | 0,55 | 56,12 | 0,18 | 0,32 | 16,25 | 0,17 | 0,01 | 0,04 | 0,06 | 0,00 | 0,01 | 0,38 | 0,06 | 0,44 |
| Point 38 | 0,00 | 2,56 | 3,97 | 11,99 | 0,48 | 0,52 | 0,00 | 0,43 | 55,27 | 0,12 | 0,13 | 23,82 | 0,16 | 0,01 | 0,02 | 0,06 | 0,00 | 0,01 | 0,17 | 0,03 | 0,26 |
| Point 39 | 0,00 | 1,92 | 3,79 | 12,29 | 0,48 | 0,48 | 0,00 | 0,43 | 50,73 | 0,11 | 0,05 | 28,96 | 0,05 | 0,01 | 0,00 | 0,11 | 0,00 | 0,01 | 0,24 | 0,05 | 0,27 |
| Point 40 | 0,00 | 2,38 | 8,76 | 26,40 | 0,77 | 0,37 | 0,02 | 1,65 | 48,50 | 0,31 | 0,03 | 10,22 | 0,02 | 0,01 | 0,02 | 0,05 | 0,00 | 0,00 | 0,04 | 0,04 | 0,41 |
| Point 41 | 0,00 | 1,97 | 5,79 | 15,96 | 0,42 | 0,30 | 0,00 | 0,59 | 50,30 | 0,10 | 0,02 | 23,71 | 0,03 | 0,02 | 0,00 | 0,08 | 0,00 | 0,00 | 0,47 | 0,10 | 0,13 |
| Point 42 | 0,00 | 2,20 | 4,85 | 14,78 | 0,47 | 0,45 | 0,00 | 0,53 | 63,71 | 0,17 | 0,03 | 11,86 | 0,04 | 0,02 | 0,01 | 0,07 | 0,01 | 0,00 | 0,38 | 0,06 | 0,36 |
| Point 43 | 0,00 | 1,72 | 3,90 | 13,51 | 0,46 | 0,47 | 0,01 | 0,44 | 64,69 | 0,15 | 0,03 | 12,32 | 1,30 | 0,02 | 0,00 | 0,07 | 0,00 | 0,00 | 0,46 | 0,08 | 0,37 |
| Point 44 | 0,00 | 1,75 | 4,31 | 14,48 | 0,44 | 0,35 | 0,01 | 0,46 | 60,15 | 0,11 | 0,04 | 16,92 | 0,02 | 0,02 | 0,00 | 0,08 | 0,00 | 0,00 | 0,48 | 0,08 | 0,28 |
| Point 45 | 0,00 | 1,55 | 4,76 | 28,28 | 0,42 | 0,41 | 0,01 | 0,49 | 42,34 | 0,10 | 0,03 | 13,72 | 7,26 | 0,02 | 0,01 | 0,10 | 0,00 | 0,00 | 0,23 | 0,05 | 0,19 |
| Point 46 | 0,00 | 1,43 | 4,45 | 11,62 | 0,49 | 0,33 | 0,00 | 0,37 | 46,79 | 0,09 | 0,03 | 33,51 | 0,07 | 0,03 | 0,00 | 0,12 | 0,00 | 0,00 | 0,47 | 0,10 | 0,09 |
| Point 47 | 0,00 | 1,26 | 3,06 | 8,28 | 0,40 | 0,52 | 0,01 | 0,35 | 69,57 | 0,09 | 0,02 | 15,24 | 0,03 | 0,02 | 0,00 | 0,08 | 0,00 | 0,00 | 0,57 | 0,09 | 0,41 |
| Point 48 | 0,00 | 1,47 | 3,95 | 15,21 | 0,44 | 0,40 | 0,00 | 0,49 | 62,73 | 0,10 | 0,04 | 12,14 | 1,97 | 0,02 | 0,00 | 0,08 | 0,00 | 0,00 | 0,49 | 0,09 | 0,37 |
| Point 49 | 0,00 | 2,24 | 5,73 | 16,66 | 0,48 | 0,20 | 0,02 | 0,66 | 62,91 | 0,19 | 0,06 | 10,02 | 0,09 | 0,02 | 0,00 | 0,05 | 0,00 | 0,01 | 0,21 | 0,04 | 0,40 |
| Point 50 | 0,37 | 1,28 | 3,33 | 29,14 | 0,26 | 0,37 | 0,01 | 0,48 | 49,93 | 0,10 | 0,03 | 6,31 | 7,83 | 0,03 | 0,01 | 0,04 | 0,00 | 0,00 | 0,13 | 0,05 | 0,29 |
| Point 51 | 0,00 | 1,71 | 3,40 | 12,44 | 0,41 | 0,56 | 0,02 | 0,41 | 65,93 | 0,36 | 0,03 | 13,72 | 0,05 | 0,03 | 0,00 | 0,07 | 0,00 | 0,00 | 0,44 | 0,08 | 0,35 |
| Point 52 | 0,70 | 1,48 | 3,31 | 33,86 | 0,26 | 0,31 | 0,01 | 0,39 | 40,90 | 0,08 | 0,01 | 8,51 | 9,55 | 0,03 | 0,00 | 0,05 | 0,00 | 0,00 | 0,26 | 0,06 | 0,23 |
| Point 53 | 0,00 | 2,21 | 3,06 | 12,31 | 0,41 | 0,37 | 0,05 | 0,40 | 44,38 | 0,12 | 0,11 | 35,27 | 0,98 | 0,01 | 0,00 | 0,15 | 0,00 | 0,02 | 0,04 | 0,02 | 0,10 |
| Point 54 | 0,00 | 2,55 | 5,55 | 16,88 | 0,55 | 0,41 | 0,00 | 0,65 | 58,37 | 0,16 | 0,13 | 13,52 | 0,02 | 0,01 | 0,00 | 0,07 | 0,00 | 0,00 | 0,58 | 0,10 | 0,45 |
| Point 55 | 0,00 | 1,92 | 4,58 | 14,12 | 0,59 | 0,51 | 0,01 | 0,58 | 71,73 | 0,13 | 0,05 | 3,47 | 0,02 | 0,01 | 0,28 | 0,03 | 0,00 | 0,00 | 0,71 | 0,10 | 1,15 |

Supplementary Table S13. MRS analysis. Measurement conditions for the spectra of figure 4 (yellow, red, brown and pink) and figure 5 (blue, green and white).

| Colour | Spectrum | Device | Laser | Laser power | Nº acc. | Exposition time |
| --- | --- | --- | --- | --- | --- | --- |
| Yellow | a1 | Qontor | 785 nm (300 mW) | 0.05 % | 50 | 1 s/acc |
|  | a2 | Reflex | 785 nm (300 mW) | 50 % | 10 | 10 s/acc |
|  | a4 | Qontor | 532 nm (50 mW) | 10 % | 30 | 1 s/acc |
|  | a6 | Reflex | 785 nm (300 mW) | 50 % | 5 | 10 s/acc |
|  | a9 | Qontor | 785 nm (300 mW) | 5 % | 3 | 10 s/acc |
|  | a10 | Reflex | 514 nm (25 mW) | 100 % | 5 | 10 s/acc |
|  | a11 | Qontor | 785 nm (300 mW) | 0.5 % | 30 | 1 s/acc |
| Red | b1 | Qontor | 785 nm (300 mW) | 0.05 % | 50 | 1 s/acc |
|  | b3 | Reflex | 785 nm (300 mW) | 50 % | 3 | 10 s/acc |
|  | b4 | Qontor | 785 nm (300 mW) | 1 % | 30 | 1 s/acc |
|  | b6 | Reflex | 785 nm (300 mW) | 50 % | 5 | 10 s/acc |
|  | b8 | InnoRam | 785 nm (300 mW) | 30 % | 100 | 2 s/acc |
|  | b9 | Qontor | 785 nm (300 mW) | 0.5 % | 3 | 10 s/acc |
|  | b10 | InnoRam | 785 nm (300 mW) | 15 % | 30 | 5 s/acc |
|  | b11 | Qontor | 785 nm (300 mW) | 0.5 % | 30 | 1 s/acc |
| Brown | c4.1 | Qontor | 473 nm (25 mW) | 10 % | 30 | 1 s/acc |
|  | c4.2 | Qontor | 473 nm (25 mW) | 5 % | 30 | 1 s/acc |
|  | c9 | Qontor | 473 nm (25 mW) | 50 % | 10 | 1 s/acc |
| Pink | d1 | Qontor | 785 nm (300 mW) | 0.05 % | 50 | 1 s/acc |
|  | d9 | Reflex | 785 nm (300 mW) | 10 % | 10 | 1 s/acc |
|  | d10 | InnoRam | 785 nm (300 mW) | 10 % | 100 | 0.1 s/acc |
| Blue | a1 | Qontor | 532 nm (50 mW) | 100 % | 50 | 1 s/acc |
|  | a2 | Reflex | 514 nm (25 mW) | 100 % | 5 | 10 s/acc |
|  | a3 | Qontor | 532 nm (50 mW) | 100 % | 50 | 1 s/acc |
|  | a5 | Qontor | 532 nm (50 mW) | 10 % | 50 | 1 s/acc |
|  | a6 | Reflex | 514 nm (25 mW) | 100 % | 5 | 10 s/acc |
|  | a7 | Reflex | 514 nm (25 mW) | 100 % | 5 | 10 s/acc |
|  | a9 | Qontor | 532 nm (50 mW) | 50 % | 20 | 1 s/acc |
|  | a10 | Reflex | 514 nm (25 mW) | 100 % | 10 | 10 s/acc |
| Green | b1 | Qontor | 532 nm (50 mW) | 10 % | 50 | 1 s/acc |
|  | b4 | Qontor | 532 nm (50 mW) | 10 % | 30 | 10 s/acc |
|  | b9 | Qontor | 532 nm (50 mW) | 50 % | 20 | 1 s/acc |
| White | c1 | Qontor | 532 nm (50 mW) | 10 % | 50 | 1 s/acc |
|  | c4 | Qontor | 785 nm (300 mW) | 0.5 % | 30 | 1 s/acc |
|  | c9 | Qontor | 532 nm (50 mW) | 10 % | 20 | 1 s/acc |

Supplementary Table S14. Total spectroscopic analyses by MRS and μEDXRF. Types of colours and decorations.

| Fragment | Colours | | | | | | | Decoration | MRS | XRF | |
| --- | --- | --- | --- | --- | --- | --- | --- | --- | --- | --- | --- |
|  | Yellow | Red | Brown | Pink | Blue | Green | White |  |  | Single spot | Map |
| 1 | x | x | x | x | x | x | x | Quiver | 37 | 37 | 1 |
| 2 | x | x |  |  | x | x | x | No figurative | 14 | 10 | - |
| 3 |  | x |  |  | x | x | x | No figurative | 6 | 11 | 1 |
| 4 | x | x | x |  |  | x | x | Little owl | 38 | 48 | 2 |
| 5 |  |  |  |  | x |  |  | No figurative | 4 | 6 | 1 |
| 6 | x | x |  |  | x | x | x | No figurative | 11 | 18 | 1 |
| 7 |  |  |  |  | x |  |  | No figurative | 3 | 4 | - |
| 8 | x | x |  |  |  | x | x | Human figure | 8 | 21 | 3 |
| 9 | x | x | x | x |  | x | x | Lar | 58 | 51 | 5 |
| 10 | x | x |  | x |  | x | x | No figurative | 19 | 26 | 1 |
| 11 | x | x | x |  |  |  | x | Goat | 12 | 55 | 4 |
| **Total analyses** | | | | | | | | | **208** | **287** | **19** |

Supplementary Table S15. μEDXRF mappings. Experimental conditions for the figures 3, 6 and 7.

| Fragment | Mapped motif | μEDXRF mapping parameters | | | | | | | |
| --- | --- | --- | --- | --- | --- | --- | --- | --- | --- |
|  |  | Voltage (kV) | Current (μA) | Spot size (μm) | Step size (μm) | Dwell time (ms) | Area  (mm^2^) | Mapping time (min) | Pixel Nº |
| 1 | Quiver | 50 | 600 | 25 | 50 | 5 | 132.00 x 85.70 | 377.1 | 4524960 |
| 4 | Left eye | 50 | 600 | 25 | 50 | 2.5 | 28.79 x 48.41 | 23.2 | 557568 |
|  | Body | 50 | 600 | 25 | 50 | 2.5 | 51.70 x 69.32 | 59.7 | 1433124 |
| 6 | Indeterminate | 50 | 600 | 25 | 50 | 2 | 65.51 x 33.27 | 29.1 | 871150 |
| 8 | Body | 50 | 600 | 25 | 50 | 2 | 53.20 x 37.60 | 26.6 | 800128 |
|  | Left arm | 50 | 600 | 25 | 50 | 2 | 35.32 x 61.55 | 28.9 | 869086 |
|  | Head | 50 | 600 | 25 | 50 | 2 | 40.47 x 26.64 | 14.4 | 431197 |
| 9 | Left arm | 50 | 600 | 25 | 50 | 2 | 61.60 x 51.10 | 42.0 | 1259104 |
|  | Clothing | 50 | 600 | 25 | 50 | 2 | 42.07 x 65.30 | 36.6 | 1098346 |
|  | Right arm | 50 | 600 | 25 | 50 | 2 | 55.13 x 85.01 | 62.5 | 1875100 |
|  | Head | 50 | 600 | 25 | 50 | 2 | 66.30 x 41.30 | 36.5 | 1095276 |
| 10 | Indeterminate | 50 | 600 | 25 | 50 | 2 | 129.55 x 38.94 | 67.3 | 2017855 |
| 11 | Legs | 50 | 600 | 25 | 50 | 2 | 28.07 x 43.22 | 16.2 | 485265 |
|  | Back | 50 | 600 | 25 | 50 | 2 | 57.89 x 16.50 | 12.7 | 382140 |
|  | Belly | 50 | 600 | 25 | 50 | 2 | 38.72 x 21.17 | 10.9 | 327402 |
|  | Head | 50 | 600 | 25 | 50 | 2 | 70.43 x 40.95 | 38.5 | 1153971 |

**Supplementary Figures**

Supplementary Figure S1. Location of Cástulo archaeological site at province of Jaén (Spain).


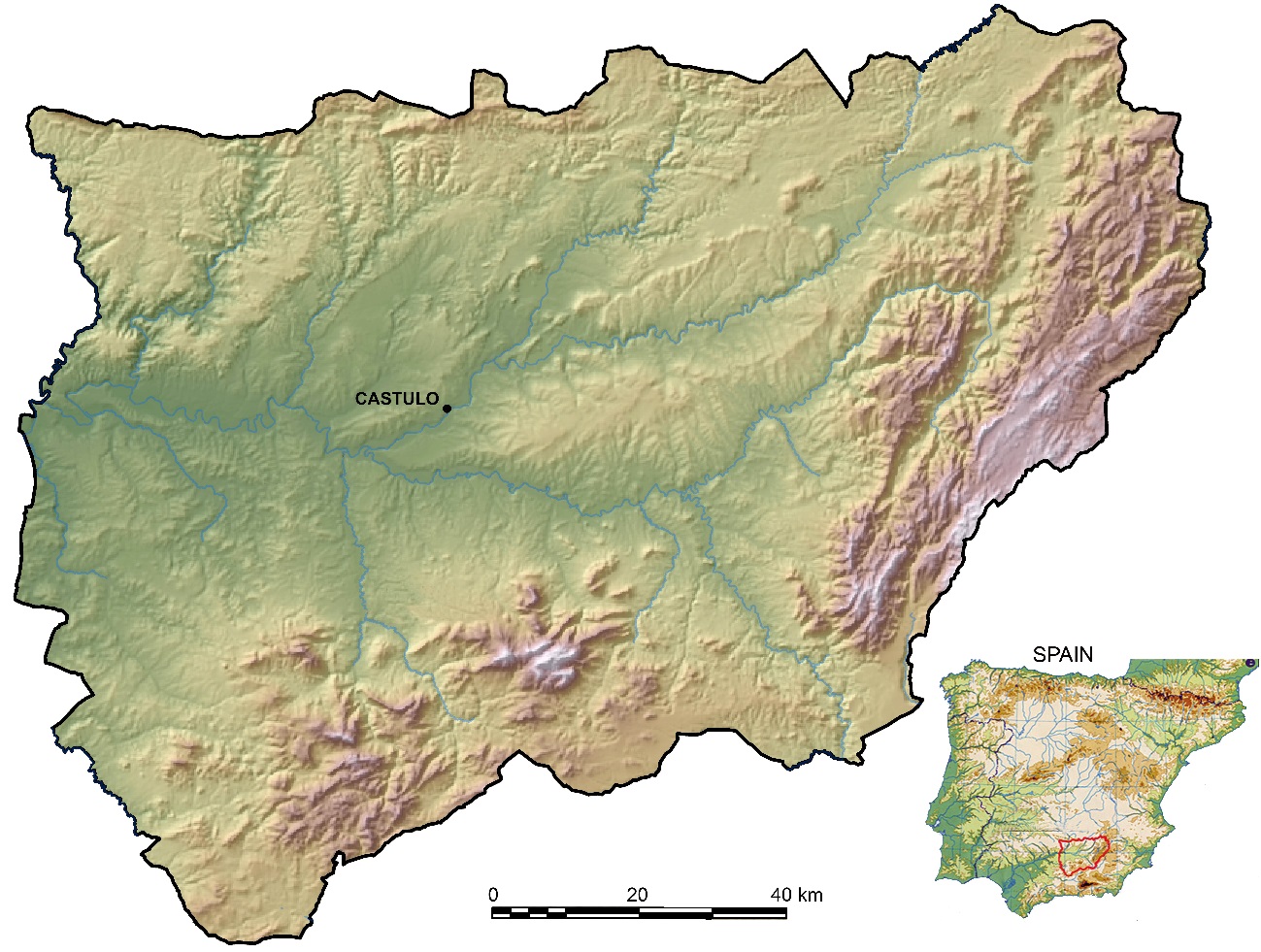


Supplementary Figure S2. Distribution of μEDXRF single-spot analyses on fragment 1.


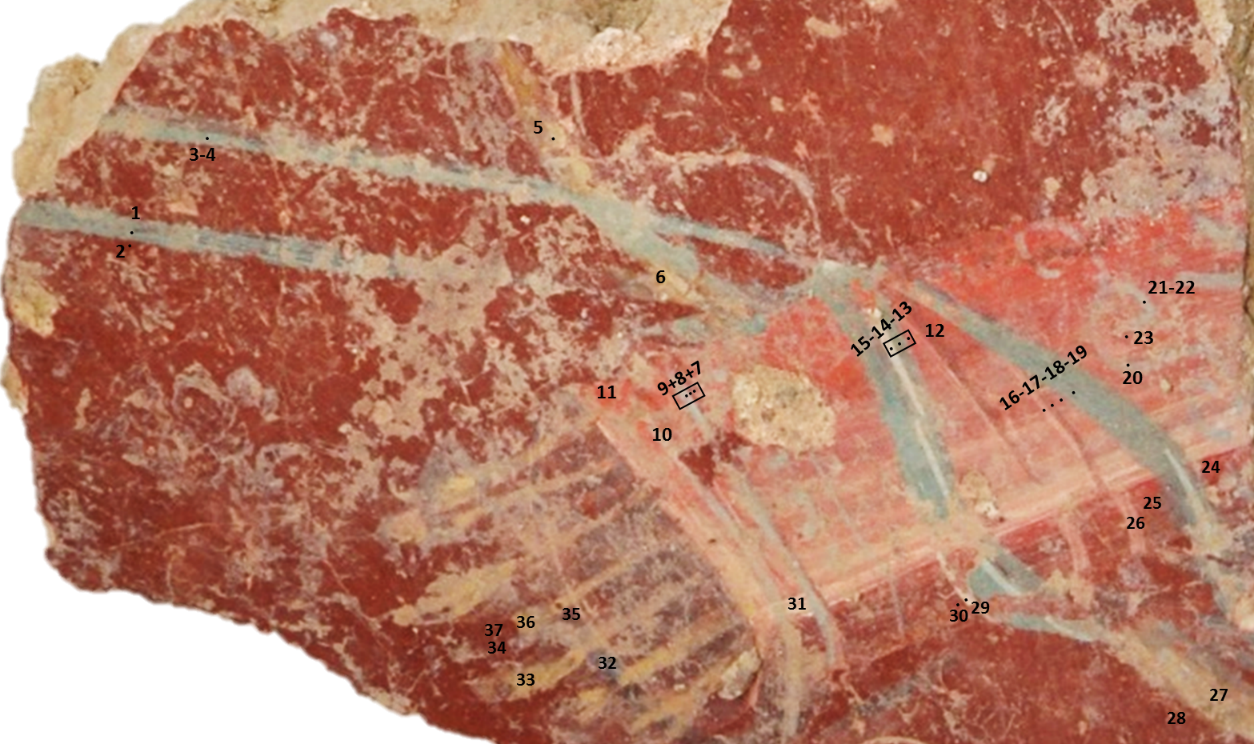


Supplementary Figure S3. Distribution of μEDXRF single-spot analyses on fragment 2.


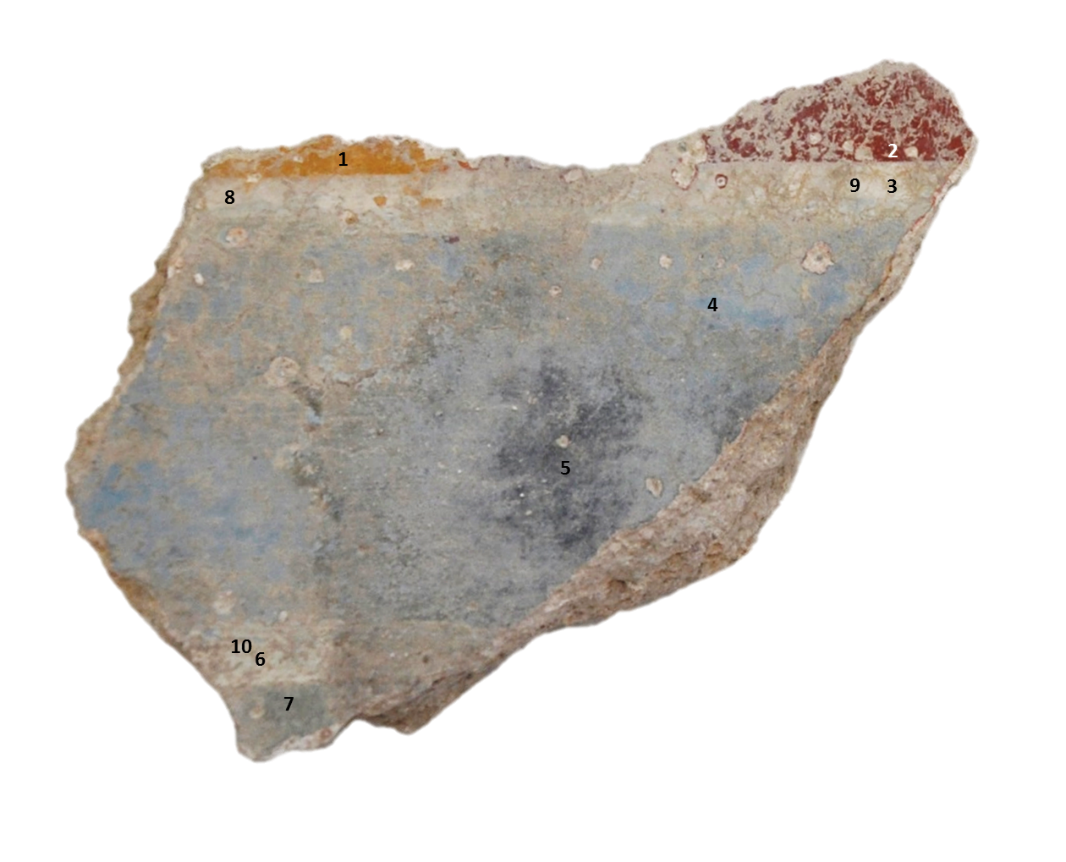


Supplementary Figure S4. Distribution of μEDXRF single-spot analyses on fragment 3.


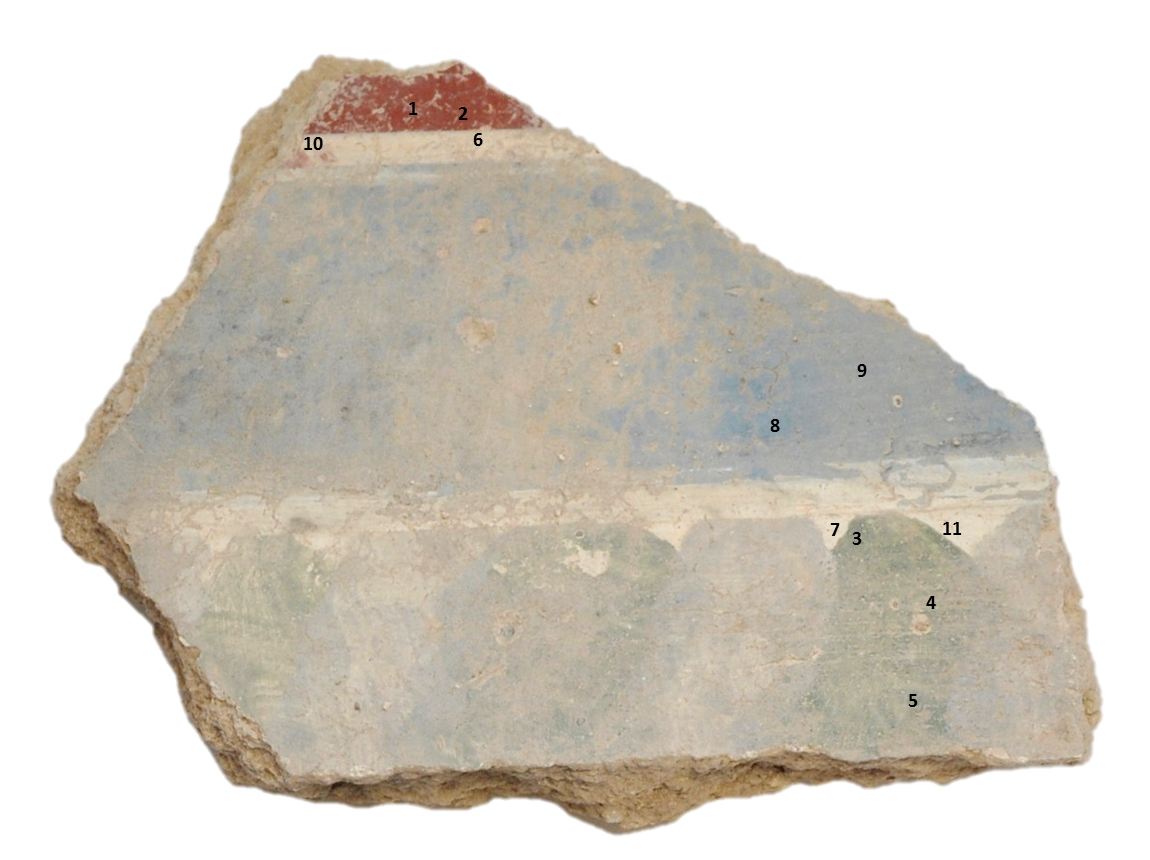


Supplementary Figure S5. Distribution of μEDXRF single-spot analyses on fragment 4.
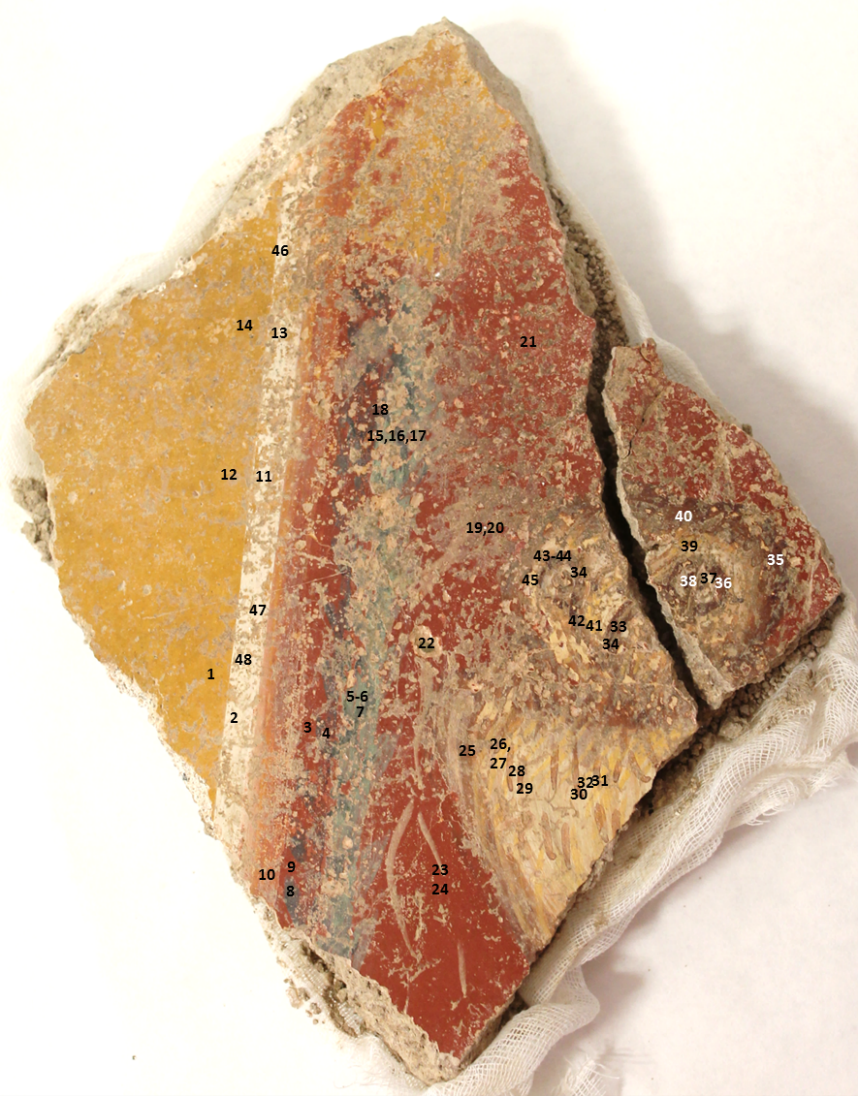


Supplementary Figure S6. Distribution of μEDXRF single-spot analyses on fragment 5.


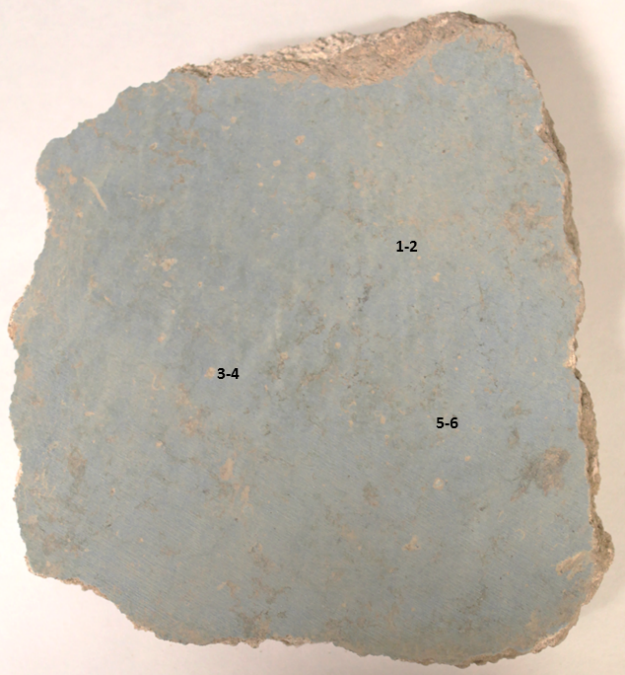


Supplementary Figure S7. Distribution of μEDXRF single-spot analyses on fragment 6.


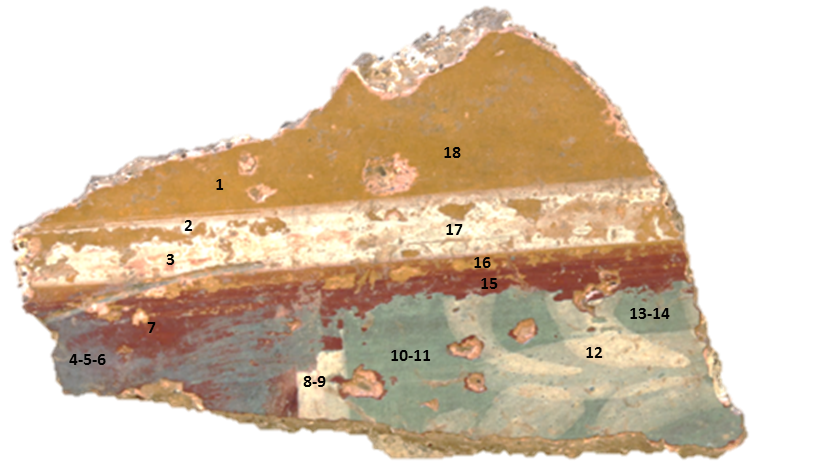


Supplementary Figure S8. Distribution of μEDXRF single-spot analyses on fragment 7.


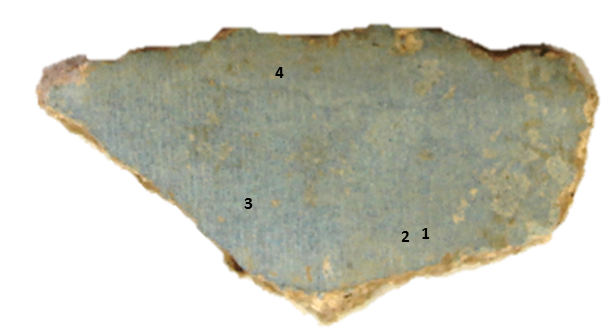


Supplementary Figure S9. Distribution of μEDXRF single-spot analyses on fragment 8.


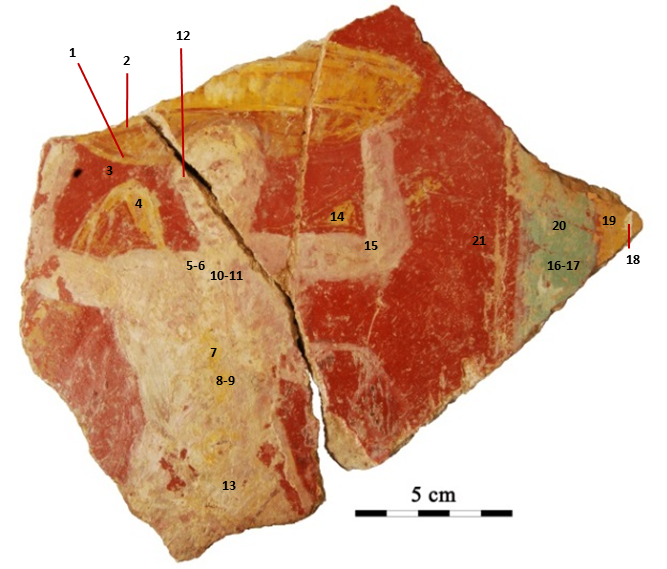


Supplementary Figure S10. Distribution of μEDXRF single-spot analyses on fragment 9.


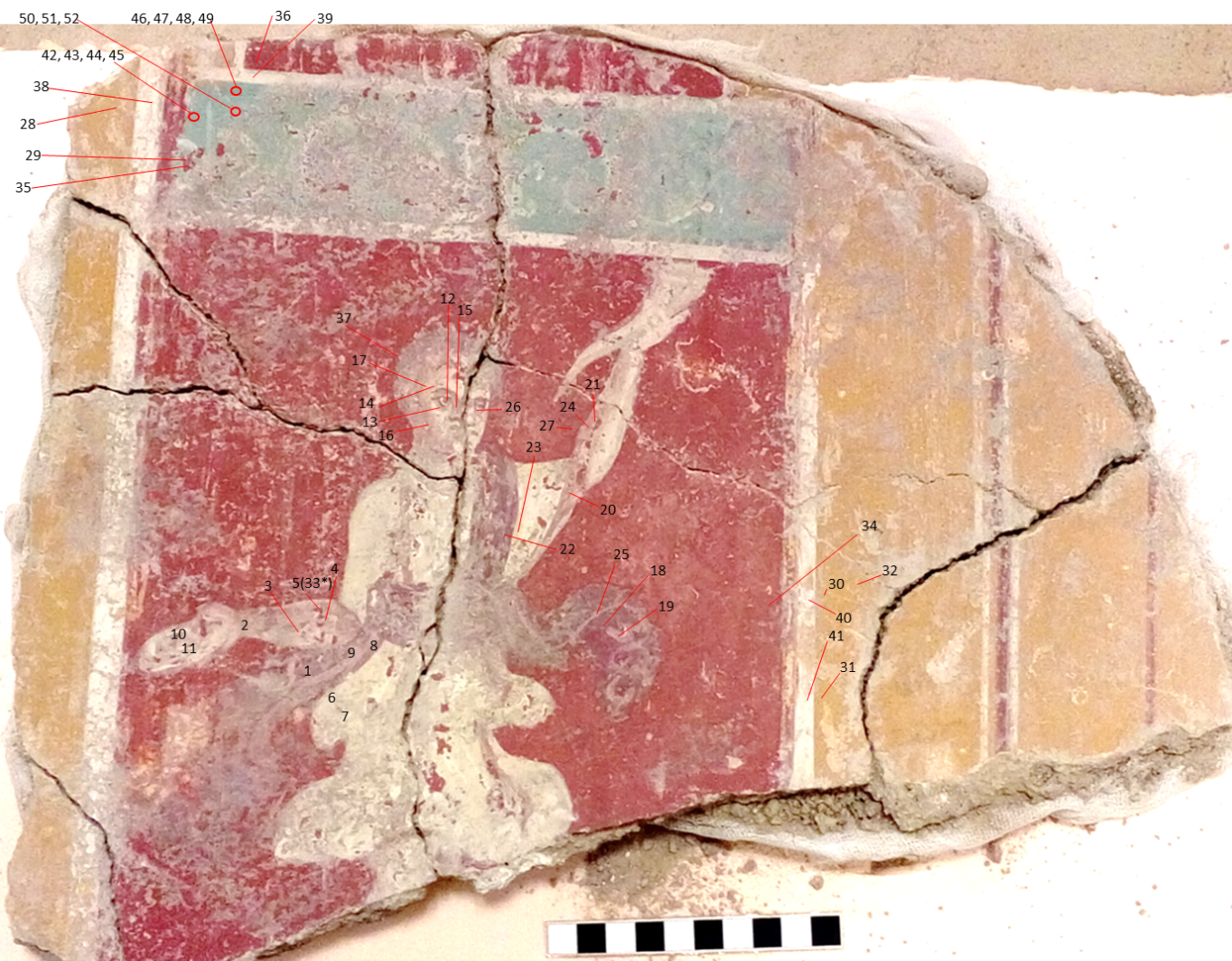


Supplementary Figure S11. Distribution of μEDXRF single-spot analyses on fragment 10.


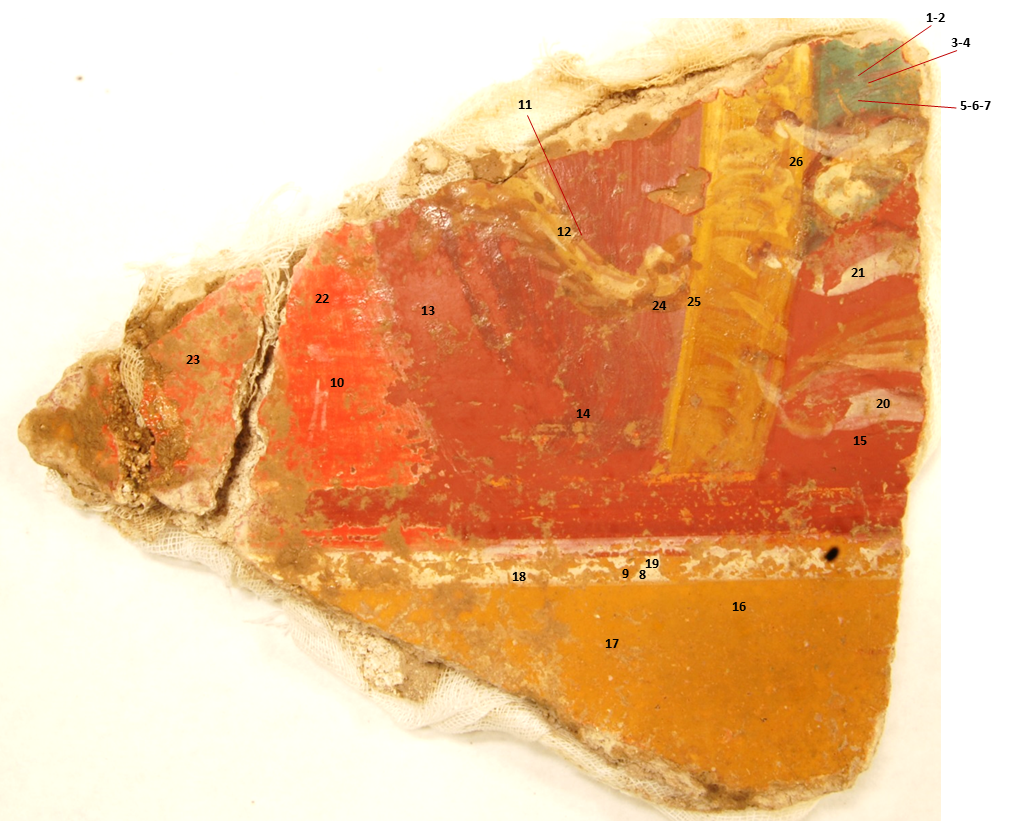


Supplementary Figure S12. Distribution of μEDXRF single-spot analyses on fragment 11.


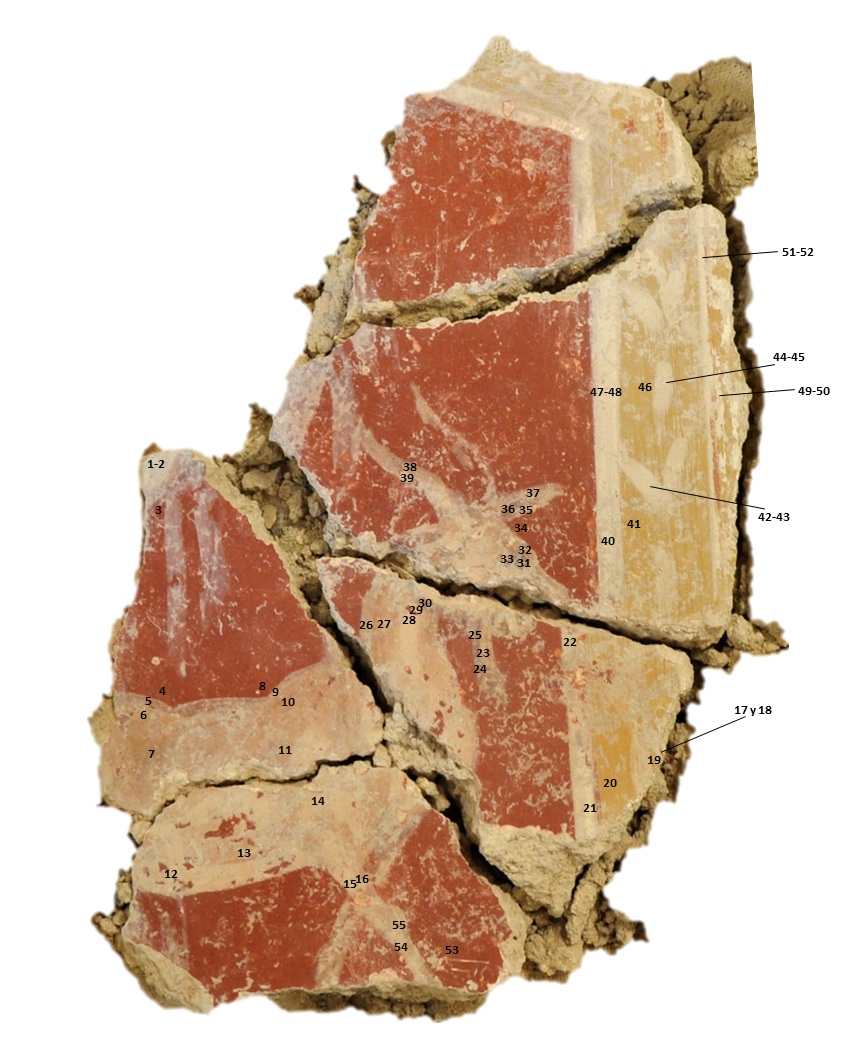


**References**

1. http://[rruff.info/goethite](http://rruff.info/goethite)
2. De Faria, D.L.A. & Lopes F.N. Heated goethite and natural hematite: Can Raman spectroscopy be used to differentiate them? *Vib Spectrosc.* 45, 117-121. <https://doi.org/10.1016/j.vibspec.2007.07.003> (2007).
3. <http://rruff.info/calcite>
4. Germinario, C. *et al*. Multi-analytical and non-invasive characterization of the polychromy of wall paintings at the Domus of Octavius Quartio in Pompeii. *Eur Phys J. Plus.* 133(9), 359. <https://doi.org/10.1140/epjp/i2018-12224-6> (2018).
5. <http://rruff.info/hematite>
6. Parras, D. *et al*. Micro-Raman spectroscopy of decorated pottery from the Iberian archaeological site of Puente Tablas (Jaén, Spain, 7th-4th century BC). *J. Raman Spectrosc*. 41, 68-73. <https://doi.org/10.1002/jrs.2405> (2010).
7. Coccato, A., Jehlick, J., Moens, L. & Vandenabeele, P. Raman spectroscopy for the investigation of carbon-based black pigments. *J. Raman Spectrosc*. 46, 1003–1015. https://doi.org/10.1002/jrs.4715 (2015).
8. <http://rruff.info/cinnabar>
9. Linn, R. Layered pigments and painting technology of the Roman wall paintings of Caesarea Maritima. *J. Archaeol Sci Rep*. 11, 774–781. <https://doi.org/10.1016/j.jasrep.2016.12.018> (2017).
10. Zoppi, A. *et al*. A novel piece of Minoan art in Italy: the ﬁrst spectroscopic study of the wall paintings from Phaistos. *J. Raman Spectrosc*.43, 1663–1670. <https://doi.org/10.1002/jrs.4029> (2012).
11. Mateos, L. D. Raman microspectroscopic analysis of decorative pigments from the Roman villa of El Ruedo (Almedinilla, Spain). *Spectrochim Acta A*. 151, 16–21.<https://doi.org/10.1016/j.saa.2015.06.091> (2015).
12. Ospitali, F., Bersani, D., Di Lonardo, G., & Lottici, P. P. ‘Green earths’: vibrational and elemental characterization of glauconites, celadonites and historical pigments. *J. Raman Spectrosc.* 39, 1066–1073. [https://doi.org/10.1002/jrs.1983 (2008](https://doi.org/10.1002/jrs.1983%20(2008)).
13. Aliatis, I. Green pigments of the Pompeian artists’ palette. *Spectrochim Acta A.* 73, 532–538. <https://doi.org/10.1016/j.saa.2008.11.009> (2009).
14. Moretto, L. M., Orsega, E. F., & Mazzocchin, G. A. Spectroscopic methods for the analysis of celadonite and glauconite in Roman green wall paintings. *J. Cult Herit*. 12, 384–391.<https://doi.org/10.1016/j.culher.2011.04.003> (2011).
15. Pérez-Rodriguez, J. L., de Haro, M. D. C. J., Siguenza, B., & Martinez-Blanes, J. M. Green pigments of Roman mural paintings from Seville Alcazar. *Appl Clay Sci.* 116–117, 211–219.<https://doi.org/10.1016/j.clay.2015.03.016> (2015).
